# Variable-coefficient parabolic theory as a high-dimensional limit of elliptic theory

**DOI:** 10.1007/s00526-023-02644-x

**Published:** 2024-01-28

**Authors:** Blair Davey, Mariana Smit Vega Garcia

**Affiliations:** 1 Department of Mathematical Sciences, Montana State University, Bozeman, MT 59717 USA; 2 Department of Mathematics, Western Washington University, Bellingham, WA 98225 USA

**Keywords:** 35J15, 35K10

## Abstract

This paper continues the study initiated in Davey (Arch Ration Mech Anal 228:159–196, 2018), where a high-dimensional limiting technique was developed and used to prove certain parabolic theorems from their elliptic counterparts. In this article, we extend these ideas to the variable-coefficient setting. This generalized technique is demonstrated through new proofs of three important theorems for variable-coefficient heat operators, one of which establishes a result that is, to the best of our knowledge, also new. Specifically, we give new proofs of $L^2 \rightarrow L^2$ Carleman estimates and the monotonicity of Almgren-type frequency functions, and we prove a new monotonicity of Alt–Caffarelli–Friedman-type functions. The proofs in this article rely only on their related elliptic theorems and a limiting argument. That is, each parabolic theorem is proved by taking a high-dimensional limit of a related elliptic result.

## Introduction

In this paper, we explore the connections between the elliptic and parabolic theory of partial differential equations, generalizing the work done by the first-named author for constant-coefficient equations in [18]. Specifically, we generalize the ideas from [18] and establish a technique that can be used to prove variable-coefficient parabolic theorems from their appropriate elliptic counterparts. The key idea is that certain parabolic estimates may be obtained by taking high-dimensional limits of their corresponding elliptic results. We obtain information about solutions to $\text {div}(A\nabla u)+\partial _t u=0$ on the parabolic side by analyzing the behavior of solutions to non-homogeneous equations of the form $\text {div}(\kappa \nabla v)=\kappa \ell$ on the elliptic side. Here, *A* has a specific structure, *v* and $\kappa$ are defined in terms of *u* and *A* (see Lemmas 2.1 and 2.2), respectively, and $\ell$ depends on both *u* and *A*. Rewriting our elliptic equation as $\displaystyle \kappa ^{-1}\text {div}(\kappa \nabla v)=\ell$, we notice that the associated operator is a special type of Witten Laplacian, or weighted Laplacian (see 2.4 in [34], and also [14, 15, 28, 34, 41–44]). From this perspective, the ideas in this article show how to obtain results for variable-coefficient parabolic operators from those for the Witten Laplacian.

Perelman first considered parabolic theory as a high-dimensional limit of elliptic theory in [47]. This general principle was discussed in the blog of Tao [52], modified in the coursenotes of Sverak [51], then developed and applied in [18]. In our setting, we follow the ideas from [18]; namely, we use classical probabilistic formulae, essentially going back to Wiener [54], with a slight modification used by Sverak in [51]. However, to account for the presence of variable-coefficients, we have modified (and complicated) the change of variables formula from [18]. Once the general framework has been established, we demonstrate the utility of this technique by establishing three new proofs of theorems regarding variable-coefficient parabolic equations. In comparison to the results of [18] for constant-coefficient equations, the techniques here are substantially modified to account for the variable-coefficients.

At the heart of the high-dimensional limiting process is a sequence of maps $F_{d,n}: \mathbb {R}^{d \times n} \rightarrow \mathbb {R}^d \times \mathbb {R}_+$ which take high-dimensional space points $y \in \mathbb {R}^{d \times n}$ on the elliptic side to space–time points $\left( x,t \right) \in \mathbb {R}^d \times \mathbb {R}_+$ on the parabolic side. These maps can be viewed from three perspectives. First, they naturally arise through certain random walk processes. Second, we can use the chain rule to see how these maps connect elliptic and parabolic operators. That is, suppose we are given some parabolic function $u = u\left( x,t \right)$ defined on $\mathbb {R}^d \times \mathbb {R}_+$ and we define $v_n = v_n(y)$ on $\mathbb {R}^{d \times n}$ by $v_n(y) = u\left( F_{d,n}(y) \right)$. A computation involving only the chain rules shows that if ${{\,\textrm{div}\,}}\left( A \nabla u \right) + \partial _t u = 0$, then ${{\,\textrm{div}\,}}(\kappa _n \nabla v_n)=\kappa _n \, \ell _n$, where $\kappa _n$ and $\ell _n$ are defined in terms of *u* and *A*. In other words, the maps $F_{d,n}$ provide a way of constructing a sequence of “elliptic" functions from a given “parabolic" function. Finally, we can see the power of these maps through their pushforward measures. In the constant-coefficient case, the pushforward by $F_{d,n}$ of the Lebesgue measure weighted with the fundamental solution of the Laplacian is a space–time Lebesgue measure that is weighted by a function that approximates the Gaussian. That is, the pushforward connects the elliptic and parabolic fundamental solutions. In our variable-coefficient setting, we do not have explicit descriptions of the fundamental solutions, so this connection is not as precise, but it mimics the constant-coefficient behavior.

Once we have the transformation maps, our general technique is as follows: Given a parabolic function *u*, we use $F_{d, n}$ to construct a sequence of functions $\left\{ v_n\right\}$. We apply an elliptic result to each of the $v_n$ functions, and then use the pushforward relationships to reinterpret this result in terms of *u*. After a limiting process, we arrive at a result for the original parabolic function *u*.

Our first new proof is of an $L^2 \rightarrow L^2$ Carleman estimate for parabolic operators of the form $\text {div}(A\nabla )+\partial _t$. For elliptic operators, the original Carleman estimates are attributed to Carleman [13], with subsequent advances by Cordes [16], Aronszajn [5] and Aronszajn et al [6]. Significant contributions to the theory of elliptic Carleman estimates with applications to strong unique continuation include the work of Jerison and Kenig [37], Sogge [50], Koch and Tataru [38], and the references therein. When $A=I$, the parabolic Carleman estimate was proved by Escauriaza in [21]; see also [24–26, 46, 53] for (variable-coefficient) generalizations that followed. The article [39] of Koch and Tataru provides a nice overview of parabolic Carleman estimates, and the results therein apply to very general parabolic operators.

Our second novel proof shows that Almgren-type frequency functions [1] associated with parabolic operators of type $\text {div}(A\nabla )+\partial _t$ are monotonically non-decreasing. In the variable-coefficient elliptic setting, Almgren-type monotonicity formulas have been used to establish unique continuation results, see [29, 30]. When $A = I$, monotonicity of the parabolic frequency function was originally proved by Poon in [49] and used to establish strong unique continuation results for caloric functions. Since then, the frequency function approach has been used extensively in the study of parabolic unique continuation problems; see for example [12, 22, 23, 40, 55].

Both the Carleman estimates and the monotonicity of the Almgren-type frequency functions, motivated by their elliptic counterparts, allowed the authors of [21, 49] (for example) to use the established techniques for elliptic theory to prove strong unique continuation for solutions to the heat equation. Since then, a wealth of unique continuation results for variable-coefficient heat operators have been established using both Carleman estimates and frequency functions. Recently, Carleman techniques have been used to establish space-like quantitative uniqueness of solutions to variable-coefficient parabolic equations that are averaged in time [56] and at a particular time-slice [7].

Almgren-type monotonicity formulas have also been used extensively in the context of free boundary problems to obtain regularity of solutions and of the free boundary. In the case of the parabolic constant-coefficient Signorini problem, a truncated version of Almgren’s monotonicity formula was proved in [17], leading to the optimal regularity of solutions and analysis of the free boundary. Elliptic variable-coefficient Almgren-type monotonicity formulas have also been widely used to study numerous free boundary problems; see for example [8, 20, 31–33, 35, 36]. We refer the reader to [48] for a beautiful introduction to the use of Almgren-type monotonicity formulas in free boundary problems.

Our third new proof establishes a result that is, to the best of our knowledge, also new. In other words, our technique leads to an original parabolic result: We prove an Alt–Caffarelli–Friedman-type (ACF-type) monotonicity formula for variable-coefficient heat operators. The groundbreaking work of Alt, Caffarelli, and Friedman in [2] introduced the use of one such monotonicity formula to study two-phase free boundary elliptic problems. Another version of this formula was proved in [9] by Caffarelli and extended by Caffarelli and Kenig in [10] to establish the regularity of solutions to parabolic equations and their singular perturbations. Later on, Caffarelli, Jerison, and Kenig [11] considered non-homogeneous elliptic equations in which the right-hand side of the equation need not vanish at the free boundary. Their main result is not a monotonicity result per se, but rather a clever uniform bound on the monotonicity functional, which is just as useful as monotonicity itself. Later on, Matevosyan and Petrosyan [45] further extended that result, proving an almost monotonicity estimate for non-homogeneous elliptic and parabolic operators with variable coefficients. We refer the interested reader to [4, 48] for an introduction to the use of the ACF monotonicity formula; [48] deals with the elliptic setting, while [4] addresses both the parabolic and elliptic settings. In the context of almost minimizers of variable-coefficient Bernoulli-type functionals, ACF-type monotonicity formulas have also been used to study the free boundary, see for example [19].

Given that our transformation maps relate solutions to homogeneous parabolic equations to a sequence of solutions to non-homogeneous elliptic equations, our proofs of the monotonicity results have added complexity. More specifically, given that each $v_n$ solves an elliptic equations with a right-hand side, we require elliptic monotonicity results that apply to solutions to non-homogeneous equations. Therefore, the first step in each parabolic monotonicity proof is to establish the related underlying variable-coefficient elliptic result with a right-hand side. These new elliptic results, which appear at the beginning of Sects. 5 and 6, may be of independent interest. We point out that these elliptic estimates generalize both the constant-coefficient and the homogeneous results.

The monotonicity of frequency functions is often proved by showing that the derivative of the frequency function has a fixed sign. In our proofs of the monotonicity of variable-coefficient Almgren-type and ACF-type frequency functions, we show that each parabolic frequency function may be described as a pointwise limit of a sequence of elliptic frequency functions. While we know that each such elliptic frequency function is differentiable and almost monotonic, that is not sufficient to guarantee the differentiability of the corresponding parabolic frequency function. Therefore, our proofs rely on a more delicate analysis that allows us to conclude directly that our parabolic frequency functions are monotonic. These ideas are described at the ends of the proofs of Theorems 5.3 and 6.4.

We work with time-independent variable-coefficient operators for which the coefficient matrix has a specific structure. That is, we consider symmetric matrices of the form $A = G G^T$, where *G* is the Jacobian of some invertible map $g: \mathbb {R}^d \rightarrow \mathbb {R}^d$. Clearly, there are bounded, elliptic coefficient matrices *A* whose structure is not of this form. However, if $A = I$, then *A* is associated to the identity map $g(z) = z$ with Jacobian $G = I$, showing that this structural condition on *A* is a reasonable generalization to constant-coefficient operators. In subsequent work, we will explore operators with even more general coefficient matrices.

The article is organized as follows. In Sects. 2 and 3, we develop the framework that connects the elliptic and parabolic theory. That is, we introduce and examine the maps $F_{d,n}: \mathbb {R}^{d \times n} \rightarrow \mathbb {R}^d \times \mathbb {R}_+$ that take points in the high-dimensional (elliptic) space to space–time (parabolic) points. Section 2 takes the perspective of random walks to introduce these maps, then uses the chain rule to explore how these maps relate elliptic and parabolic operators. Section 3 examines these maps from a measure theoretic perspective. In particular, we present the pushforward computations and describe how the integrals on the elliptic side are related to those on the parabolic side. These two sections contain a collection of calculations and statements that will be referred to throughout the article.

The $L^2 \rightarrow L^2$ variable-coefficient parabolic Carleman estimate is proved in Sect. 4. The Almgren-type frequency function theorem for variable-coefficient heat operators is presented in Sect. 5. Finally, Sect. 6 contains the monotonicity result for Alt–Caffarelli–Friendman-type energies associated with variable-coefficient heat operators.

## Elliptic-to-parabolic transformations

In this section, we construct the transformations that connect so-called parabolic functions $u = u(x,t)$ defined on $\mathbb {R}^d \times \left( 0, T \right)$ to a sequence of elliptic functions $v_n = v_n(y)$ defined in $\mathbb {R}^{d \times n}$ for all $n \in \mathbb {N}$. More specifically, for each $n \in \mathbb {N}$, we construct a mapping of the form

$$\begin{aligned} F_{d,n} : \mathbb {R}^{d \times n}&\rightarrow \mathbb {R}^d \times \mathbb {R}_+ \\ y&\mapsto (x,t) \end{aligned}$$

that takes element *y* in (high-dimensional) space $\mathbb {R}^{d \times n}$ to elements (*x*, *t*) in space–time $\mathbb {R}^d \times \mathbb {R}_+$. Given a function $u = u(x,t)$ defined on a space–time domain, i.e. a subset of $\mathbb {R}^d \times \mathbb {R}_+$, we use $F_{d,n}$ to define a function $v_n = v_n(y)$ on the space $\mathbb {R}^{d \times n}$ by setting $v_n(y) = u(F_{d,n}(y))$. As we show below via the chain rule, if *u* is a solution to a backward parabolic equation, then each $v_n$ is a solution to some non-homogeneous elliptic equation. This observation explains why we think of *u* as a parabolic function and of each $v_n$ as an elliptic function. Moreover, as *n* becomes large, the function $v_n$ behaves (heuristically) more and more like a solution to a homogeneous elliptic equation. As such, the transformation $F_{d,n}$ becomes more useful to our purposes as $n \rightarrow \infty$, thereby illuminating why the notion of a high-dimensional limit is relevant here. From another perspective, when we use $F_{d,n}$ to pushforward measures on spheres and balls in $\mathbb {R}^{d \times n}$, we produce measures in space–time that are weighted by approximations to generalized Gaussians. This perspective is explored in the next section, Sect. 3.

We can think of the transformations $F_{d,n}: \mathbb {R}^{d \times n} \rightarrow \mathbb {R}^d \times \mathbb {R}_+$ in a number of ways. On one hand, as we show in this section, these maps are constructed so that each $v_n$ solves an elliptic equation whenever *u* solves a parabolic equation. This viewpoint is purely computational as these relationships are illuminated by the chain rule. This perspective is perhaps the most (easily) checkable, but it is somewhat mysterious. Random walks are the underlying mechanism in defining these mappings, so we begin with that perspective.

Let $y = \left( y_{1,1}, y_{1,2}, \ldots , y_{1,n}, \ldots , y_{d,1}, y_{d,2}, \ldots , y_{d,n} \right) \in \mathbb {R}^{d \times n}$ denote the variables that play the role of the “random steps" in our random walk. For some $t > 0$, assume that *y* satisfies

1 $$\begin{aligned} 2dt = \sum _{i = 1}^d \sum _{j = 1}^n y_{i,j}^2. \end{aligned}$$

In this setting, we do not fix the step size but simply assume that *y* is uniformly distributed over the sphere of radius $\sqrt{2dt}$. Define

2 $$\begin{aligned} z_i = y_{i,1} + y_{i,2} + \cdots + y_{i, n} \quad \text { for } i = 1, \ldots , d \end{aligned}$$

so that $z = \left( z_1, \ldots , z_d \right) \in \mathbb {R}^d$. In the notation from [18], we define $f_{d,n}: \mathbb {R}^{d \times n} \rightarrow \mathbb {R}^d$ so that

3 $$\begin{aligned} z = f_{d,n}(y). \end{aligned}$$

Now let

$$\begin{aligned} g: \mathbb {R}^d \rightarrow \mathbb {R}^d \end{aligned}$$

be an invertible function with inverse function

$$\begin{aligned} h: \mathbb {R}^d \rightarrow \mathbb {R}^d. \end{aligned}$$

Set $x = g(z) \in \mathbb {R}^d$ so that

4 $$\begin{aligned} x_i = g_i(z) \quad \text { for } i = 1, \ldots , d \end{aligned}$$

and then since $z = h(x)$ we have

$$\begin{aligned} z_i = h_i(x)\quad \text { for } i = 1, \ldots , d. \end{aligned}$$

For the moment, assume that *g* is sufficiently regular for the computations below to hold in a weak sense. When further regularity is needed, we specify it.

The Jacobian of $g = \left( g_1, \ldots , g_d \right)$ is a $d \times d$ invertible matrix function whose inverse matrix is the Jacobian of $h = \left( h_1, \ldots , h_d \right)$. Let *G* and *H* denote the Jacobian matrices of *g* and *h*, respectively. That is,

5 $$\begin{aligned} G(z) = \begin{bmatrix} \frac{ \partial g_1}{ \partial z_1} &{}\quad \frac{ \partial g_1}{ \partial z_2}&{}\quad \ldots &{}\quad \frac{ \partial g_1}{ \partial z_d} \\ \frac{ \partial g_2}{ \partial z_1} &{}\quad \frac{ \partial g_2}{ \partial z_2}&{}\quad \ldots &{}\quad \frac{ \partial g_2}{ \partial z_d} \\ \vdots &{} \ddots &{} &{}\quad \vdots \\ \frac{ \partial g_d}{ \partial z_1} &{} \quad \frac{ \partial g_d}{ \partial z_2}&{}\quad \ldots &{}\quad \frac{ \partial g_d}{ \partial z_d} \end{bmatrix}, \quad H(x) = \begin{bmatrix} \frac{ \partial h_1}{ \partial x_1} &{}\quad \frac{ \partial h_1}{ \partial x_2} &{}\quad \ldots &{}\quad \frac{ \partial h_1}{ \partial x_d} \\ \frac{ \partial h_2}{ \partial x_1} &{}\quad \frac{ \partial h_2}{ \partial x_2}&{}\quad \ldots &{}\quad \frac{ \partial h_2}{ \partial x_d} \\ \vdots &{}\quad \ddots &{} &{}\quad \vdots \\ \frac{ \partial h_d}{ \partial x_1} &{}\quad \frac{ \partial h_d}{ \partial x_2} &{}\quad \ldots &{}\quad \frac{ \partial h_d}{ \partial x_d} \end{bmatrix}. \end{aligned}$$

Let $\gamma (z) = \det G(z)$ and $\eta (x) = \det H(x)$. From (5), we obtain

$$\begin{aligned} I&= G(z)H(x) = G(z)H(g(z)) = G(h(x))H(x) \\ I&= H(x)G(z)= H(x) G(h(x)) = H(g(z))G(z) \\ 1&= \gamma (z) \eta (x) = \gamma (h(x)) \eta (x) = \gamma (z) \eta (g(z)). \end{aligned}$$

Now we collect some observations that follow from the Chain Rule. For the first lemma, we describe how these determinants and their derivatives transform through the map $g \circ f_{d,n}$.

### Lemma 2.1

(Determinant Chain Rule Lemma) For $\gamma (z) = \det G(z)$ and $\eta (x) = \det H(x)$ as above, define $\kappa _n: \mathbb {R}^{d \times n} \rightarrow \mathbb {R}$ to satisfy

6 $$\begin{aligned} \kappa _n(y) = \gamma (f_{d,n}(y)) = \gamma (z) = \frac{1}{\eta \left( x \right) } = \frac{1}{\eta \left( g(f_{d,n}(y)) \right) }. \end{aligned}$$

Then

$$\begin{aligned} \begin{aligned} \frac{ \partial \log \kappa _n}{ \partial y_{i,j}}&= \text {tr}\left( H \left( g(z) \right) \, \frac{ \partial G(z)}{ \partial z_i} \right) = \text {tr}\left( H \left( x \right) \, \frac{ \partial G}{ \partial z_i}\left( h(x) \right) \right) \\ y \cdot \nabla _y \log \kappa _n&= \text {tr}\left( H(g(z)) \left\langle \nabla G(z), z \right\rangle \right) = \text {tr}\left( H(x) \left\langle \nabla _z G(h(x)), h(x) \right\rangle \right) . \end{aligned} \end{aligned}$$

### Proof

Since $\displaystyle \frac{ \partial z_k}{ \partial y_{i,j}} = \delta _{k, i}$, then an application of Jacobi’s formula shows that

7 $$\begin{aligned} \frac{ \partial \log \kappa _n(y)}{ \partial y_{i,j}}&= \frac{ \partial \log \gamma (z)}{ \partial z_i} = \text {tr}\left( G^{-1}(z) \frac{ \partial G(z)}{ \partial z_i} \right) = \text {tr}\left( H \left( g(z) \right) \, \frac{ \partial G(z)}{ \partial z_i} \right) . \end{aligned}$$

We then get

$$\begin{aligned} y \cdot \nabla _y \log \kappa _n&= \sum _{i=1}^d \sum _{j = 1}^n y_{i, j} \frac{ \partial \log \kappa _n(y)}{ \partial y_{i,j}} = \sum _{i=1}^d z_{i} \frac{ \partial \log \gamma (z)}{ \partial z_{i}} = \left\langle \nabla \log \gamma (z), z \right\rangle \\&= \text {tr}\left[ H(g(z)) \left\langle \nabla G(z) , z \right\rangle \right] . \end{aligned}$$

The next set of observations shows how the derivatives of some parabolic function $u: \mathbb {R}^d \times \left( 0, T \right) \rightarrow \mathbb {R}$ can be related to those of its associated elliptic functions $v_n: B_{\sqrt{2dT}} \subset \mathbb {R}^{d \times n} \rightarrow \mathbb {R}$. That is, given a parabolic function $u = u(x,t)$, we define the elliptic functions $v_n = v_n(y) = u(F_{d,n}(y))$, where

8 $$\begin{aligned} F_{d,n}(y)=(x,t)=\left( \left( g\circ f_{d,n} \right) (y), \frac{|y|^2}{2d} \right) . \end{aligned}$$

The following set of results justifies why we refer to *u* as parabolic and each $v_n$ as elliptic.

### Lemma 2.2

(Solution Chain Rule Lemma) Given $u: \mathbb {R}^d \times \left( 0, T \right) \rightarrow \mathbb {R}$, define $v_n: B_{\sqrt{2dT}} \subset \mathbb {R}^{d \times n} \rightarrow \mathbb {R}$ to satisfy

$$\begin{aligned} v_n(y) = u\left( F_{d,n}(y) \right) . \end{aligned}$$

Define $B = B(z)$ to be a $d \times d$ matrix function with entries

$$\begin{aligned} b_{k, \ell } = \nabla _z g_k \cdot \nabla _z g_\ell = \sum _{i=1}^d \frac{ \partial g_k}{ \partial z_i}\frac{ \partial g_\ell }{ \partial z_i}. \end{aligned}$$

That is, $B =G G^T$. We then set $A(x) = B\left( h(x) \right) = B(z)$ so that $A = H^{-1}\left( H^{-1} \right) ^T$. Then

$$\begin{aligned} \begin{aligned} \frac{ \partial v_n}{ \partial y_{i,j}}&= \left\langle \nabla _x u, \frac{ \partial g}{ \partial z_i} \right\rangle + \frac{ \partial u}{ \partial t} \frac{y_{i, j}}{d} \\ y \cdot \nabla _y v_n&= \left\langle \nabla _x u, G(z) z \right\rangle + 2 t \frac{ \partial u}{ \partial t} = \left\langle A(x)\nabla _x u, {H(x)^T h(x)} \right\rangle + 2 t \frac{ \partial u}{ \partial t} \\ \left| \nabla _y v_n\right| ^2&= n \left| G(z)^T \nabla _x u\right| ^2 + \frac{2}{d} \frac{ \partial u}{ \partial t} \left[ \left\langle \nabla _x u, G(z) \, z \right\rangle + t \frac{ \partial u}{ \partial t} \right] \\&= n \left\langle A(x) \nabla _x u, \nabla _x u \right\rangle + \frac{2}{d} \frac{ \partial u}{ \partial t} \left[ \left\langle A(x)\nabla _x u, H(x)^T h(x) \right\rangle + t \frac{ \partial u}{ \partial t} \right] . \end{aligned} \end{aligned}$$

Moreover, with $\kappa _n$ as in (6),

9 $$\begin{aligned} \frac{{{\,\textrm{div}\,}}_y \left( \kappa _n(y)\nabla _y v_n \right) }{\kappa _n(y)}&= n \left[ {{\,\textrm{div}\,}}_x \left( A \nabla _x u \right) + \frac{ \partial u}{ \partial t}\right] + \frac{2}{d}\left[ \left\langle {A\nabla _x \frac{ \partial u}{ \partial t}, H^T h}\right\rangle \right. \nonumber \\&\left. \quad + \frac{ \partial u}{ \partial t} \frac{\text {tr}\left( H \left\langle \nabla _z G(h), h \right\rangle \right) }{2} + t \frac{ \partial ^2 u}{ \partial t^2} \right] , \end{aligned}$$

where the expression on the right depends on *x* and *t*.

The proof of this result relies on the chain rule and can be found in “Appendix A”. When *u* is a solution to a variable-coefficient backwards heat equation, we immediately reach the following consequence.

### Corollary 2.3

(Chain Rule Corollary) If $u: \mathbb {R}^d \times \mathbb {R}_{+} \rightarrow \mathbb {R}$ is a solution to ${{\,\textrm{div}\,}}_x \left( A \nabla _x u \right) + \partial _t u = 0$ and we define $v_n: \mathbb {R}^{d \times n} \rightarrow \mathbb {R}$ to satisfy

$$\begin{aligned} v_n(y) = u\left( F_{d,n}(y) \right) , \end{aligned}$$

then

$$\begin{aligned} {{\,\textrm{div}\,}}_y \left( \kappa _n(y)\nabla _y v_n \right) = \kappa _n(y) \ell _n(y), \end{aligned}$$

where

$$\begin{aligned} \ell _n = \frac{2}{d} \left[ \left\langle A\nabla _x \frac{ \partial u}{ \partial t}, H^T h \right\rangle + \frac{1}{2} \frac{ \partial u}{ \partial t} \text {tr}\left( H \left\langle \nabla _z G(h), h \right\rangle \right) - t {{\,\textrm{div}\,}}_x \left( A \nabla _x \frac{ \partial u}{ \partial t} \right) \right] . \end{aligned}$$

In summary, we have constructed a sequence of maps $F_{d,n}$, given in (8), that serve as the connection between the elliptic and parabolic settings. The following table describes these relationships and the notation that we use to describe our elliptic and parabolic settings. Note that $F_{d,n}$ is the connection between the elliptic column and the parabolic column.

$$\begin{aligned} \begin{array}{l|ll} &{} \text {Elliptic} &{}\quad \text {Parabolic} \\ \hline \hline \text {Space} &{} \text {high-dimensional space} &{}\quad \text {space--time} \\ \text {Elements} &{} y \in \mathbb {R}^{d \times n} &{}\quad \left( x, t \right) \in \mathbb {R}^d \times \mathbb {R}_+ \\ \text {Functions} &{} v_n = v_n(y) &{}\quad u = u\left( x,t \right) \\ \text {PDEs} &{} {{\,\textrm{div}\,}}_y \left( \kappa _n \nabla _y v_n \right) = \kappa _n \, \ell _n &{}\quad {{\,\textrm{div}\,}}_x \left( A \nabla _x u \right) + \partial _t u = 0 \\ \end{array} \end{aligned}$$

Going forward, we write $\nabla u$ to indicate $\nabla _x u$, unless explicitly indicated otherwise. Similarly, we write $\nabla v_n$ to indicate $\nabla _y v_n$, unless explicitly indicated otherwise. That is, *u* is understood to be a function of *x* and *t*, while $v_n$ is a function of *y*, and all derivatives are interpreted appropriately.

The results above will be used extensively when we prove our parabolic theorems. Therefore, we work with variable-coefficient operators for which the coefficient matrix has the specific structure that is described by the previous two results. That is, we consider $A(x) = B(z)$ where $B = G G^T$ and *G* is the Jacobian of some invertible map $g: \mathbb {R}^d \rightarrow \mathbb {R}^d$. Clearly, there are bounded, elliptic coefficient matrices *A* whose structure is not of this form. However, if $A = I$, then *A* is associated to the identity map $g(z) = z$ with Jacobian $G = I$, showing that this structural condition on *A* is a reasonable generalization to constant-coefficient operators.

Before concluding this section, we examine some examples of non-trivial coefficient matrices that satisfy our structural condition. For simplicity, we assume that $d=1$.

Given a bounded, elliptic function $\lambda \le C(x)\le \Lambda$, we construct a function *g* such that the corresponding function *A*(*x*) coincides with *C*(*x*). Recall that

$$\begin{aligned} A(x)=G(h(x))G^T(h(x))=\left[ g'(h(x))\right] ^2. \end{aligned}$$

For $C(x)=A(x)$ to hold, we need $\sqrt{C(x)}=g'(h(x))$. Since $g = h^{-1}$ and $h = g^{-1}$, then $\displaystyle g'(h(x)) = \frac{1}{h'(x)}$, so we need to solve $h'(x)=\frac{1}{\sqrt{C(x)}}$.

### Example 2.4

Let $C(x)=\left( \frac{1+x^2}{2+x^2}\right) ^2$. Notice that $\frac{1}{4}\le C(x)\le 1$ and $\displaystyle \frac{1}{\sqrt{C(x)}} = \frac{2+x^2}{1+x^2}$ so that

$$\begin{aligned} \int \displaystyle \frac{dx}{\sqrt{C(x)}} =\int \frac{2+x^2}{1+x^2}dx =x+\arctan (x)+c. \end{aligned}$$

If we want $h(0) = 0$, then define $h(x) = x + \arctan (x)$ and take $g(z)=h^{-1}(z)$.

### Example 2.5

Let $C(x)=2+\sin (x)$ and notice that $1\le C(x)\le 3.$ We define

$$\begin{aligned} h(x) =\int \frac{1}{\sqrt{2+\sin (x)}}dx =\frac{-2}{\sqrt{3}} {F\left( \frac{\pi -2x}{4}\Big |\frac{2}{3}\right) }, \end{aligned}$$

where *F*(*x*|*m*) is the elliptic integral of the first kind with parameter $m=k^2$. Again, we define $g(z)=h^{-1}(z)$.

## Integral relationships

In what follows, we examine how integrals of functions $\phi (x,t)$ defined in space–time $\mathbb {R}^d \times \mathbb {R}_+$ can be related to integrals of $\phi (F_{d,n}(y))$ in high-dimensional space $\mathbb {R}^{d \times n}$. We start from a probabilistic viewpoint. That is if $y \in \mathbb {R}^{d \times n}$ is uniformly distributed over a fixed sphere, we seek to determine the probability distribution of $x \in \mathbb {R}^d$, where $(x,t) = F_{d,n}(y)$. As we show below, understanding this probability distribution on the parabolic side reduces to a pushforward computation which we carry out explicitly. We then collect the consequences that will be used in our elliptic-to-parabolic proofs.

Let $S_t^n$ denote the sphere of radius $\sqrt{2dt}$ in $\mathbb {R}^{d\times n}$. That is,

10 $$\begin{aligned} S_t^n=\left\{ y\in \mathbb {R}^{d\times n} \, \ |y|^2 = 2dt\right\} . \end{aligned}$$

Let $\sigma _{d n-1}^{t}$ denote the canonical surface measure on this sphere $S_t^n$. We make the assumption that the vectors $y = (y_{1,1},y_{1,2},\ldots ,y_{d,1},\ldots ,y_{d,n}) \in \mathbb {R}^{d \times n}$ are uniformly distributed over $S_t^n$ with respect to $\sigma _{d n-1}^{t}$. Set $\mu _{d,n}^{t}$ to be the normalized surface measure on the sphere $S^n_t$; that is,

$$\begin{aligned} \mu _{d, n}^{t}=\frac{1}{|S^{d n-1}|(2dt)^{\frac{d n-1}{2}}} \sigma _{d n-1}^{t}. \end{aligned}$$

Our goal is to find the probability distribution of *x* in the limit as $n\rightarrow \infty$, where $x=g(z) = g(f_{d,n}(y))$ and $z=f_{d,n}(y)$ is as in (3).

We first define an intermediate measure on $z \in \mathbb {R}^d$ for each fixed *t* via the pushforward as $\omega _{d,n}^t = f_{d, n} \# \mu _{d, n}^t$. This is the pushforward from [18] and it is shown there that

$$\begin{aligned} d\omega _{d,n}^t&= \frac{\left| S^{d n-1 - d}\right| }{\left| S^{d n-1}\right| \left( 2 d n t \right) ^{\frac{d}{2}}} \left( 1 - \frac{z_1^2 + \cdots + z_{d}^2}{2 d n t} \right) ^{\frac{d n - d - 2}{2}} \chi _{B_{nt}}(z) \, dz_1 \ldots dz_{d} \\&= \frac{\left| S^{d n -1 - d}\right| }{\left| S^{d n-1}\right| \left( 2d n t \right) ^{\frac{d}{2}}} \left( 1 - \frac{\left| z\right| ^2}{2 d n t} \right) ^{\frac{d n - d - 2}{2}} \chi _{B_{nt}}(z) \, dz, \end{aligned}$$

where we have introduced the notation

11 $$\begin{aligned} B_{nt}=\{z\in \mathbb {R}^d \, \ |z|^2 < 2dnt\} \end{aligned}$$

for the ball of radius $\sqrt{2dnt}$ in $\mathbb {R}^d$.

The probability distribution of *x* is the push-forward of $\mu _{d,n}^{t}$ by $g \circ f_{d, n}$:

$$\begin{aligned} \nu _{d,n}^{t}:=(g\circ f_{d,n})\#\big (\mu _{d,n}^{t}\big ). \end{aligned}$$

This implies, in particular, that for all $\varphi \in C_0(\mathbb {R}^d)$,

$$\begin{aligned} \int _{\mathbb {R}^d}\varphi (x) \, d\nu _{d,n}^{t}(x)=\int _{S_t^n}\varphi (g(f_{d,n}(y))) \, d\mu _{d,n}^{t}(y). \end{aligned}$$

Now observe that

$$\begin{aligned} \nu _{d,n}^t = \left( g \circ f_{d,n} \right) \# \mu _{d,n}^t = g \# \left( f_{d,n} \# \mu _{d,n}^t \right) = g \# \omega _{d,n}^t. \end{aligned}$$

Since $g: \mathbb {R}^d \rightarrow \mathbb {R}^d$ is invertible, computing the pushforward here is the same as carrying out a change of variables. By definition, we have that

$$\begin{aligned}&\frac{\left| S^{d n-1 - d}\right| }{\left| S^{d n-1}\right| \left( 2 d n t \right) ^{\frac{d}{2}}} \int _{\mathbb {R}^d} \varphi \left( g(z) \right) \left( 1 - \frac{\left| z\right| ^2}{2 d n t} \right) ^{\frac{d n - d - 2}{2}} \chi _{B_{nt}}(z) \, dz \\&\quad = \int _{\mathbb {R}^d} \varphi \left( g(z) \right) d \omega _{d, n}^t = \int _{\mathbb {R}^d} \varphi \left( x \right) d\nu _{d, n}^t. \end{aligned}$$

Since $dx = \gamma (z) \, dz$, $\eta (g(z)) \gamma (z) =1$, and $z = h(g(z))$, then

$$\begin{aligned}&\int _{\mathbb {R}^d} \varphi \left( g(z) \right) \left( 1 - \frac{\left| z\right| ^2}{2 d n t} \right) ^{\frac{d n - d - 2}{2}} \chi _{B_{nt}}(z) \, dz\\&\quad = \int _{\mathbb {R}^d} \varphi \left( g\left( z \right) \right) \left( 1 - \frac{\left| z\right| ^2}{2 d n t} \right) ^{\frac{d n - d - 2}{2}} \eta \left( g\left( z \right) \right) \chi _{B_{nt}(z)}\, \gamma (z) \, dz \\&\quad = \int _{\mathbb {R}^d} \varphi \left( x \right) \eta \left( x \right) \left( 1 - \frac{\left| h\left( x \right) \right| ^2}{2 d n t} \right) ^{\frac{d n - d - 2}{2}} \chi _{B_{nt}}(h(x))\, dx. \end{aligned}$$

It follows that $\displaystyle \nu _{d,n}^t = \eta (x) \, K_{t,n}(h(x)) \, dx$, where we introduce

12 $$\begin{aligned} K_{t,n}(x) = K_{n}(x, t) =\frac{\left| S^{d n -1 - d}\right| }{\left| S^{d n -1}\right| \left( 2 d n t \right) ^{\frac{d}{2}}} \left( 1 - \frac{\left| x\right| ^2}{2 d n t} \right) ^{\frac{d n - d - 2}{2}}\chi _{B_{nt}}(x). \end{aligned}$$

As shown in [18, Lemma 6],

13 $$\begin{aligned} K_t(x) = K(x,t) := \lim _{n \rightarrow \infty } K_{t,n}(x)&= \left( \frac{1}{4 \pi t} \right) ^{\frac{d}{2}} \exp \left( -\frac{\left| x\right| ^2}{4t} \right) , \end{aligned}$$

where the limit is pointwise. A much stronger version of convergence holds for this sequence, stated as follows.

### Lemma 3.1

(Uniform convergence of $\left\{ K_n\right\}$) Given any $t_0 >0$, the sequence $\left\{ K_n(x,t)\right\} _{n=1}^\infty$ converges uniformly to *K*(*x*, *t*) in $\mathbb {R}^d\times \{t\ge t_0\}$.

The proof of this result can be found in “Appendix A”. In fact, the arguments there, in combination the proof of [18, Lemma 1], show that there exists ${\mathcal {C}}_d$ so that for every $n \in \mathbb {N}$ and every $\left( x,t \right) \in \mathbb {R}^d \times \mathbb {R}_+$,

14 $$\begin{aligned} K_{t,n}(x) \le \mathcal {C}_d K_{t}(x). \end{aligned}$$

This bound will be used in many of our subsequent arguments.

One might wonder how our transformed heat kernel relates to the standard one. The following lemma, proved in “Appendix A”, shows that *K*(*h*(*x*), *t*) is not a solution to the associated homogeneous variable-coefficient heat equation.

### Lemma 3.2

(Kernel solution) With $u_K(x,t) = K\left( h(x), t \right)$, it holds that

$$\begin{aligned} {{\,\textrm{div}\,}}\left( A \nabla u_K \right) - \partial _t u_K = - \frac{1}{2t} \sum _{ j, k, \ell =1}^d \frac{ \partial ^2 g_k}{ \partial {z_j} \partial z_\ell }(h(x)) \frac{ \partial h_\ell }{ \partial x_k} h_j\, u_K \ne 0. \end{aligned}$$

Summarizing our pushforward computations, we have established the following result:

### Lemma 3.3

(cf. Lemma 2 and Lemma 3 in [18]) Let $S_{t}^n$, $K_{t,n}$ and $K_t$ be as given in (10), (12) and (13), respectively. If $\varphi : \mathbb {R}^d \rightarrow \mathbb {R}$ is integrable with respect to $K_{t}\left( h(x) \right) d{x}$, then for every $n \in \mathbb {N}$, $\varphi$ is integrable with respect to $K_{t,n}\left( h(x) \right) d{x}$ and it holds thatwhere $\kappa _n$ is as defined in (6).

### Remark 3.4

Going forward, we often use the notation $d \sigma$ or $d \sigma (y)$ in place of $d \sigma _{d n -1}^t$.

It is also helpful to interpret the measures $\nu _{d,n}^t$ as time slices of a space–time object, which comes from the projection of some global measure in the space $y\in \mathbb {R}^{d\times n}$ onto the space–time. To do so, we project a measure $\mu _{d,n}$ on $\mathbb {R}^{d\times n}$ to the space–time $\mathbb {R}^d\times \mathbb {R}_+$ by the map $F_{d,n}$ as given in (8). With *m* denoting Lebesgue measure, define

$$\begin{aligned} \mu _{d,n}=\frac{d}{|S^{d n-1}||y|^{d n -2}}m. \end{aligned}$$

The “slices” of the measure $\mu _{d,n}$ by the spheres $S^t_n$ project by $F_{d,n}$ onto the measures $\nu _{d,n}^t$; that is,

$$\begin{aligned} F_{d,n}\# \mu _{d,n}=\int _0^{\infty }(g\circ f_{d,n})\#\big (\mu _{d,n}^t\big )dt=\int _0^{\infty }\nu _{d,n}^tdt. \end{aligned}$$

Let $B_t^n$ denote the open ball in $\mathbb {R}^{d \times n}$ for which $\partial B_n^t = S_n^t$, i.e.

15 $$\begin{aligned} B_t^n=\left\{ y\in \mathbb {R}^{d\times n} \, \ |y|^2 < 2dt\right\} . \end{aligned}$$

Integrating the equality from Lemma 3.3 leads to the following result. 455

### Lemma 3.5

(cf. Lemma 4 in [18]) Let $B_t^n$, $K_{n}$ and *K* be as given in (15), (12) and (13), respectively. If $\phi : \mathbb {R}^d \times \left( 0, T \right) \rightarrow \mathbb {R}$ is integrable with respect to $K\left( h(x) \right) d{x} \, dt$, then for every $n \in \mathbb {N}$, $\phi$ is integrable with respect to $K_{n}\left( h(x) \right) d{x} \, dt$ and for every $t \in \left( 0, T \right)$, it holds that

$$\begin{aligned}&\frac{1}{d \left| S^{d n -1}\right| } \int _{B^n_t} \kappa _n(y) \phi \left( F_{d,n}\left( y \right) \right) \left| y\right| ^{2 - d n} d{y} = \int _0^t \int _{\mathbb {R}^d} \phi \left( x,s \right) K_n\left( h(x),s \right) d{x} \, d{s}, \end{aligned}$$

where $\kappa _n$ is as defined in (6). In fact, if $T = \infty$, then

$$\begin{aligned}&\frac{1}{d \left| S^{d n -1}\right| } \int _{\mathbb {R}^{d \times n}} \kappa _n(y) \phi \left( F_{d,n}\left( y \right) \right) \left| y\right| ^{2 - d n } d{y} = \int _0^\infty \int _{\mathbb {R}^d} \phi \left( x,s \right) \, K_n\left( h(x),s \right) \, d{x} \, d{s}. \end{aligned}$$

Lemmas 3.3 and 3.5 will be used many times in our proofs below. Lemma 3.3 transforms a $\kappa _n$-weighted integral over a sphere in $\mathbb {R}^{d \times n}$ to a $K_{t,n}$-weighted integral at a particular slice in space–time. Similarly, Lemma 3.5 transforms a weighted integral over a ball in $\mathbb {R}^{d \times n}$ to a $K_{t,n}$-weighted integral in space–time. Of note, the elliptic weight in Lemma 3.5 is $\kappa _n$ times the fundamental solutions of the Laplacian, while by (13), the parabolic weight is an approximation to a transformed heat kernel. As dimension-limiting arguments will be crucial to our proofs below, we need to understand what happens when $n \rightarrow \infty$. By (13) and 14 in combination with the Dominated Convergence Theorem, the parabolic integrals in both of these lemmas converge to *K*(*h*(*x*), *t*)-weighted integrals.

For the remainder of this section, we introduce some definitions and draw some conclusions based on Lemmas 3.3 and 3.5.

### Definition 3.6

(Weighted $L^1$ spaces) Given $\phi : \mathbb {R}^d \times \left( 0, T \right) \rightarrow \mathbb {R}$ and $h: \mathbb {R}^d \rightarrow \mathbb {R}^d$ as in Sect. 2, define $\mathcal {P}\left( \cdot ; \phi , h \right) : \left( 0, T \right) \rightarrow \mathbb {R}$ as

$$\begin{aligned} \mathcal {P}\left( t; \phi , h \right)&= \int _{\mathbb {R}^d} \left| \phi (x,t)\right| \exp \left( -\frac{\left| h(x)\right| ^2}{4t} \right) dx. \end{aligned}$$

We say that $\mathcal {P}\left( \cdot ; \phi , h \right) \in L^1\left( \left[ 0, t\right] , s^{- \frac{d}{2}}ds \right)$ if

$$\begin{aligned} \int _0^t s^{- \frac{d}{2}} \int _{\mathbb {R}^d} \left| \phi (x,s)\right| \exp \left( -\frac{\left| h(x)\right| ^2}{4s} \right) dx \, ds < \infty . \end{aligned}$$

With this definition and Lemma 3.5, we reach the following result.

### Corollary 3.7

(Weighed integrability on the elliptic side) Let $\phi : \mathbb {R}^d \times \left( 0, T \right) \rightarrow \mathbb {R}$, $h: \mathbb {R}^d \rightarrow \mathbb {R}^d$ and assume that $\mathcal {P}\left( \cdot ; \phi , h \right) \in L^1\left( \left[ 0, t\right] , s^{- \frac{d}{2}}ds \right)$. For each $\psi _n: B_T^n \subset \mathbb {R}^{d \times n} \rightarrow \mathbb {R}$ defined by $\psi _n(y) = \phi \left( F_{d,n}(y) \right)$, $\psi _n$ is integrable with respect to $\kappa _n(y) \, dy$ in $B_t^n$ for every $n \ge 2$.

### Proof

An application of Lemma 3.5 shows that

$$\begin{aligned} \int _{B_t^n} \kappa _n(y) \left| \psi _n(y)\right| dy&= \int _{B_t^n} \kappa _n(y) \left| \phi \left( F_{d,n}(y) \right) \right| \left| y\right| ^{dn-2} \left| y\right| ^{2-dn} dy \\&= d \left| S^{d n -1}\right| \int _{0}^t \int _{\mathbb {R}^d} \left| \phi \left( x,s \right) \right| \left( 2ds \right) ^{\frac{dn-2}{2}} K_n\left( (h(x), s \right) dx ds \\&\le d \left| S^{d n -1}\right| \mathcal {C}_d \int _{0}^t \int _{\mathbb {R}^d} \left| \phi (x,s)\right| \left( 2ds \right) ^{\frac{dn-2}{2}} K\left( (h(x), s \right) dx ds \\&\le \frac{\left( 2dt \right) ^{\frac{dn}{2}} \mathcal {C}_d \left| S^{d n -1}\right| }{2t\left( 4 \pi \right) ^{\frac{d}{2}}} \int _{0}^t s^{-\frac{d}{2}} \mathcal {P}\left( s; \phi , h \right) ds, \end{aligned}$$

where we have applied (14) and that $dn \ge 2d \ge d$. $\square$

Now we introduce some function classes for parabolic and elliptic functions that will be used below.

### Definition 3.8

(Moderate *h*-growth at infinity) Let $u: \mathbb {R}^d \times \left( 0, T \right) \rightarrow \mathbb {R}$ be a continuous function with locally integrable weak first order derivatives. With $h: \mathbb {R}^d \rightarrow \mathbb {R}^d$ as in Sect. 2 and $A = H^{-1} \left( H^{-1} \right) ^T$, define

16 $$\begin{aligned} \begin{aligned} \mathcal {H}\left( t \right) = \mathcal {H}\left( t; u,h \right)&= \int _{\mathbb {R}^d} \left| u(x,t)\right| ^2 \exp \left( -\frac{\left| h(x)\right| ^2}{4t} \right) dx \\ \mathcal {D}\left( t \right) = \mathcal {D}\left( t; u,h \right)&= \int _{\mathbb {R}^d} \left\langle A(x) \nabla u(x,t), \nabla u(x,t) \right\rangle \exp \left( -\frac{\left| h(x)\right| ^2}{4t} \right) dx \\ \mathcal {T}\left( t \right) = \mathcal {T}\left( t; u,h \right)&= \int _{\mathbb {R}^d} \left| \frac{ \partial u}{ \partial t}(x,t)\right| ^2 \exp \left( - \frac{\left| h(x)\right| ^2}{4t} \right) dx. \end{aligned} \end{aligned}$$

We say that such a function *u* has *moderate h-growth at infinity* if $\mathcal {H}$, $\mathcal {D}$, and $\mathcal {T}$ belong to $L^1\left( \left[ 0, T\right] , t^{- \frac{d}{2}}dt \right)$.

### Definition

($\kappa$-weighted Sobolev space) For $B_R \subset \mathbb {R}^N$, we say that a function $v: B_R \rightarrow \mathbb {R}$ belongs to $L^{p}\left( B_R, \kappa (y) \, dy \right)$, the space of $\kappa$-*weighted p-integrable functions*, if

$$\begin{aligned}&\int _{B_R} \kappa (y) \left| v(y)\right| ^p dy < \infty . \end{aligned}$$

Moreover, if both *v* and $\nabla v \in L^2\left( B_R, \kappa (y) \, dy \right)$, then we say that *v* belongs to the $\kappa$*-weighted Sobolev space* and write $v \in W^{1,2}\left( B_R, \kappa (y) \, dy \right)$.

Now we may connect these two function classes.

### Lemma 3.9

(Function class relationship) If $u: \mathbb {R}^d \times \left( 0, T \right) \rightarrow \mathbb {R}$ has moderate *h*-growth at infinity, then for $v_n: B_T^n \subset \mathbb {R}^{d \times n} \rightarrow \mathbb {R}$ defined by $v_n(y) = u\left( F_{d,n}(y) \right)$ and $\kappa _n$ defined by (6), it holds that $v_n \in W^{1,2}\left( B_T^n, \kappa _n(y) dy \right)$, whenever $n \ge 2$.

### Proof

An application of Lemma 3.5 shows that

$$\begin{aligned} \int _{B_T^n} \kappa _n(y) \left| v_n(y)\right| ^2 dy&= \int _{B_T^n} \kappa _n(y) \left| u\left( F_{d,n}(y) \right) \right| ^2 \left| y\right| ^{dn-2} \left| y\right| ^{2-dn} dy \\&= d \left| S^{d n -1}\right| \int _{0}^T \int _{\mathbb {R}^d} \left| u(x,s)\right| ^2 \left( 2ds \right) ^{\frac{dn-2}{2}} K_n\left( (h(x), s \right) dx ds \\&\le \frac{\left( 2d \right) ^{\frac{dn}{2}} \mathcal {C}_d \left| S^{d n -1}\right| }{2\left( 4 \pi \right) ^{\frac{d}{2}}} \int _{0}^T s^{\frac{dn-d-2}{2}} \mathcal {H}\left( s \right) ds, \end{aligned}$$

where we have applied (14). Since *u* has moderate *h*-growth at infinity, then

$$\begin{aligned} \int _{B_T^n} \kappa _n(y) \left| v_n(y)\right| ^2 dy \le \frac{\left( 2dT \right) ^{\frac{dn}{2}} \mathcal {C}_d \left| S^{d n -1}\right| }{2T\left( 4 \pi \right) ^{\frac{d}{2}}} \left\| \mathcal {H}\right\| _{L^1\left( \left[ 0,T\right] , t^{- \frac{d}{2}} dt \right) } < \infty . \end{aligned}$$

For the gradient term, we use Lemma 3.5 in combination with Lemma 2.2 to get

$$\begin{aligned}&\int _{B_T^n} \kappa _n(y) \left| \nabla v_n(y)\right| ^2 dy\\&\quad = d n \left| S^{d n -1}\right| \int _{0}^T \int _{\mathbb {R}^d} \left\langle A\nabla u, \nabla u \right\rangle \left( 2ds \right) ^{\frac{dn-2}{2}} K_n\left( (h(x), s \right) dx ds \\&\qquad + \left| S^{d n -1}\right| \int _{0}^T \int _{\mathbb {R}^d} 2s \left( \frac{ \partial u}{ \partial s} \right) ^2\left( 2ds \right) ^{\frac{dn-2}{2}} K_n\left( (h(x), s \right) dx ds \\&\qquad + \left| S^{d n -1}\right| \int _{0}^T \int _{\mathbb {R}^d} 2 \frac{ \partial u}{ \partial s} \left\langle \left( H^{-1} \right) ^T\nabla u, h \right\rangle \left( 2ds \right) ^{\frac{dn-2}{2}} K_n\left( (h(x), s \right) dx ds \\&\quad \le 2 d n \left| S^{d n -1}\right| \int _{0}^T \int _{\mathbb {R}^d} \left\langle A\nabla u, \nabla u \right\rangle \left( 2ds \right) ^{\frac{dn-2}{2}} K_n\left( (h(x), s \right) dx ds \\&\qquad + 2\left| S^{d n -1}\right| \int _{0}^T \int _{\mathbb {R}^d} s \left( 1 + \frac{\left| h(x)\right| ^2}{2dns} \right) \left( \frac{ \partial u}{ \partial s} \right) ^2\left( 2ds \right) ^{\frac{dn-2}{2}} K_n\left( (h(x), s \right) dx ds, \end{aligned}$$

where the last step uses Cauchy–Schwarz. Since $\left| h(x)\right| ^2 \le 2 d n s$ on the support of $K_n(h(x))$, then

$$\begin{aligned}&\int _{B_T^n} \kappa _n(y) \left| \nabla v_n(y)\right| ^2 dy\\&\quad \le \frac{\left( 2d \right) ^{\frac{dn}{2}} \mathcal {C}_d n \left| S^{d n -1}\right| }{\left( 4\pi \right) ^{\frac{d}{2}}} \left( \int _{0}^T s^{\frac{dn-d-2}{2}} \mathcal {D}\left( s \right) ds + \frac{2}{dn} \int _{0}^T s^{\frac{dn-d}{2}} \mathcal {T}\left( s \right) ds \right) \\&\quad \le \frac{\left( 2dT \right) ^{\frac{dn}{2}} \mathcal {C}_d n \left| S^{d n -1}\right| }{T\left( 4\pi \right) ^{\frac{d}{2}}} \left( \left\| \mathcal {D}\right\| _{L^1\left( \left[ 0,T\right] , t^{- \frac{d}{2}} dt \right) } +\quad \frac{2T}{dn} \left\| \mathcal {T}\right\| _{L^1\left( \left[ 0,T\right] , t^{- \frac{d}{2}} dt \right) } \right) < \infty , \end{aligned}$$

where we have again used that *u* has moderate *h*-growth at infinity. $\square$

## Carleman estimates

Carleman estimates have played a significant role in the development of the theory of unique continuation for both elliptic and parabolic equations. The original idea (used in the elliptic setting) is attributed to Carleman [13], with subsequent work accomplished by Cordes [16], Aronszajn [5] and Aronszajn et al [6]. Since then, a wealth of results have been produced for both elliptic and parabolic operators; see for example [21, 24–26, 37–39, 46, 50, 53]. Additional applications of Carleman estimates include geometry, inverse problems, and control theory.

The following elliptic Carleman estimate is the $L^2 \rightarrow L^2$ case of Theorem 1 from [3]. The original theorem was used to establish unique continuation properties of functions that satisfy partial differential inequalities of the form $\left| \Delta v\right| \le \left| V\right| \left| v\right|$, for $v \in W^{2,q}_{loc}\left( \Omega \right)$, $V \in L^w_{loc}\left( \Omega \right)$, where $w > \frac{N}{2}$, and $\Omega \subset \mathbb {R}^N$ is open and connected.

### Proposition 4.1

([3], Theorem 1) For any $\tau \in \mathbb {R}$ and all $v \in W^{2,2}_0\left( \mathbb {R}^N {\setminus } \left\{ 0\right\} \right)$, the following inequality holds

17 $$\begin{aligned} c\left( \tau , N \right) \left\| \left| y\right| ^{-\tau } v\right\| _{L^2\left( \mathbb {R}^N \right) } \le \left\| \left| y\right| ^{-\tau +2} \Delta v\right\| _{L^2\left( \mathbb {R}^N \right) }, \end{aligned}$$

where

$$\begin{aligned} c\left( \tau , N \right) = \inf _{\ell \in \mathbb {Z}_{\ge 0}} \left| \left( \frac{N}{2} + \ell + \tau - 2 \right) \left( \frac{N}{2} + \ell - \tau \right) \right| . \end{aligned}$$

### Remark 4.2

In order for this theorem to be meaningful, we choose $\tau \in \mathbb {R}$ so that $\tau - \frac{N}{2} \not \in \mathbb {Z}_{\ge 0}$.

### Remark 4.3

Other versions of this theorem hold with more general norms. More specifically, [3, Theorem 1] establishes that for any $1 \le q \le 2 \le p < \infty$ and $\mu := 2 - \frac{N}{w} = 2 - N \left( \frac{1}{q} - \frac{1}{p} \right) > 0$,

$$\begin{aligned} \left\| \left| y\right| ^{-\tau } v\right\| _{L^p\left( \mathbb {R}^N \right) } \lesssim \left\| \left| y\right| ^{-\tau +\mu } \Delta v\right\| _{L^q\left( \mathbb {R}^N \right) }. \end{aligned}$$

However, since the condition that $\mu > 0$ is equivalent to $N < \frac{2 p q}{p-q}$, we must have that *p* and *q* are both very close to 2 for large *N*. In particular, when $N \rightarrow \infty$, $p, q \rightarrow 2$. Since we use the high-dimensional limit of this elliptic Carleman estimate to establish its parabolic counterpart, this explains why we restrict to the $L^2 \rightarrow L^2$ Carleman estimate.

The following parabolic Carleman estimate is a variable-coefficient $L^2 \rightarrow L^2$ version of Theorem 1 from [21], and it resembles [24, Theorem 4]. For a much more general result, we refer the reader to [39, Theorem 3]. The original theorem was used to prove strong unique continuation of solutions to the heat equation. As such, this version could be used to establish unique continuation results for solutions to variable-coefficient heat equations.

### Theorem 4.4

(c.f. [21], Theorem 1) For *H* is as in (5) and $\eta = \det H$, assume that $\left( H^{-1} \right) ^T \nabla \log \eta \in L^\infty \left( \mathbb {R}^d \right)$ and ${{\,\textrm{div}\,}}\left[ H^{-1} \left( H^{-1} \right) ^T\nabla \log \eta \right] \in L^\infty \left( \mathbb {R}^d \right)$. Let $d \ge 1$ and take $\alpha \in \mathbb {R}$ so that $2\alpha - \frac{d}{2} - 3 \in \left( 0, \infty \right) {\setminus } \mathbb {Z}$. Define $\varepsilon := {{\,\textrm{dist}\,}}\left( 2\alpha - \frac{d}{2} - 1, \mathbb {Z}_{\ge 0} \right)$ and for some $\delta \in \left( 0,1 \right)$, set

$$\begin{aligned} T_0 = \varepsilon \sqrt{1-\delta } \left\| 2 {{\,\textrm{div}\,}}\left[ H^{-1} \left( H^{-1} \right) ^T\nabla \log \eta \right] + \left| \left( H^{-1} \right) ^T \nabla \log \eta \right| ^2\right\| _{L^\infty \left( \mathbb {R}^d \right) }^{-1}. \end{aligned}$$

Then there is a constant *C*, depending only on $\varepsilon$ and $\delta$, such that for every $u \in C^\infty _0\left( \mathbb {R}^{d} \times \left( 0, T_0 \right) {\setminus } \left\{ \left( 0,0 \right) \right\} \right)$, it holds that

$$\begin{aligned}&\int _0^{T_0} \int _{\mathbb {R}^d} \left| u\left( x,t \right) \right| ^2 t^{-2\alpha } e^{-\frac{\left| h(x)\right| ^2}{4t}} dx \, dt \\&\quad \le C\left( \varepsilon , \delta \right) \int _0^{T_0} \int _{\mathbb {R}^d} \left| {{\,\textrm{div}\,}}\left( A \nabla u \right) + \partial _t u\right| ^2 t^{-2\alpha + 2} e^{-\frac{\left| h(x)\right| ^2}{4t}} dx \, dt, \end{aligned}$$

where *h* is some invertible function and $A = H^{-1} \left( H^{-1} \right) ^T$.

We show that Theorem 4.4 follows from the elliptic result, Proposition 4.1, Lemma 3.5, and the results of Sect. 2. More specifically, given *u*, we define a sequence of functions $\left\{ v_n\right\}$ and we then apply Proposition 4.1 to each one. Applications of Lemma 3.5 allow us to transform the integral inequalities for $v_n$ into integral inequalities for *u*. By taking a limit of this sequence of inequalities and using (13), we arrive at our conclusion.

### Proof

Let $u \in C^\infty _0\left( \mathbb {R}^{d} \times \left( 0, T_0 \right) {\setminus } \left\{ \left( 0,0 \right) \right\} \right)$. For every $n \in \mathbb {N}$, let $v_{n}: \mathbb {R}^{d \times n} \rightarrow \mathbb {R}$ satisfy

$$\begin{aligned} v_{n}\left( y \right) = u\left( F_{d, n}\left( y \right) \right) , \end{aligned}$$

where $F_{d,n}$ is as defined in (8). Note that each $v_n$ is compactly supported in $B_{T_0}^n = B_{\sqrt{2 d T_0}}$. With $\gamma (z) = \frac{1}{\eta (x)}$, define $\kappa _n: \mathbb {R}^{d \times n} \rightarrow \mathbb {R}$ to satisfy

$$\begin{aligned} \kappa _{n}\left( y \right) = \frac{1}{\eta \left( g(f_{d, n}(y)) \right) }. \end{aligned}$$

For $\alpha \in \mathbb {R}$ so that $2\alpha - \frac{d}{2} - 3 \in \left( 0, \infty \right) {\setminus } \mathbb {Z}$, set $\tau _n = 2\alpha + \frac{d n - d - 2}{2}$. Since $\tau _n - \frac{d n}{2} = 2\alpha - \frac{d}{2} - 1 \in \left( 2, \infty \right) {\setminus } \mathbb {Z}$, then $\tau _n - \frac{d n}{2} \not \in \mathbb {Z}_{\ge 0}$.

Since

$$\begin{aligned} \Delta \left( \sqrt{\kappa }_n v_n \right)&= {{\,\textrm{div}\,}}\left( \sqrt{\kappa }_n \nabla v_n + \frac{\nabla \kappa _n}{2 \sqrt{\kappa }_n} v_n \right) = \sqrt{\kappa }_n \Delta v_n + \frac{\nabla \kappa _n}{\sqrt{\kappa }_n} \cdot \nabla v_n + {{\,\textrm{div}\,}}\left( \frac{\nabla \kappa _n}{2 \sqrt{\kappa }_n} \right) v_n \\&= \frac{1}{\sqrt{\kappa }_n} {{\,\textrm{div}\,}}\left( \kappa _n \nabla v_n \right) - {{\,\textrm{div}\,}}\left( \kappa _n \nabla \left( \kappa _n^{-1/2} \right) \right) v_n, \end{aligned}$$

then for $\delta > 0$ as given,

$$\begin{aligned} \left| \Delta \left( \sqrt{\kappa }_n v_n \right) \right| ^2&\le C_\delta \kappa _n \left[ \frac{1}{\kappa _n} {{\,\textrm{div}\,}}\left( \kappa _n \nabla v_n \right) \right] ^2 + \frac{c_\delta }{\kappa _n} \left[ {{\,\textrm{div}\,}}\left( \kappa _n \nabla \left( \kappa _n^{-1/2} \right) \right) \right] ^2 \left| \sqrt{\kappa }_n v_n\right| ^2, \end{aligned}$$

where $C_\delta = {1 + \delta ^{-1}}$ and $c_\delta = 1 + \delta$. An application of Theorem 4.1 with $v = \sqrt{\kappa }_n v_n$ and $\tau = \tau _n$ shows that

18 $$\begin{aligned} \begin{aligned}&c\left( \tau _n, d n \right) ^2 \int _{B_{T_0}^n}\left| \sqrt{\kappa }_n v_n\right| ^2 \left| y\right| ^{-2\tau _n} dy\\&\quad \le \int _{B_{T_0}^n} \left| \Delta \left( \sqrt{\kappa }_n v_n \right) \right| ^2 \left| y\right| ^{-2\tau _n +4} dy \\&\quad \le C_\delta \int _{B_{T_0}^n} \kappa _n \left[ \frac{1}{\kappa _n} {{\,\textrm{div}\,}}\left( \kappa _n \nabla v_n \right) \right] ^2 \left| y\right| ^{-2\tau _n +4} dy \\&\qquad + c_\delta \int _{B_{T_0}^n} \left[ \frac{\left| y\right| ^2}{\sqrt{\kappa _n}} {{\,\textrm{div}\,}}\left( \kappa _n \nabla \left( \kappa _n^{-1/2} \right) \right) \right] ^2 \left| \sqrt{\kappa }_n v_n\right| ^2 \left| y\right| ^{-2\tau _n } dy. \end{aligned} \end{aligned}$$

Since $u \in C^\infty _0\left( \mathbb {R}^{d} \times \left( 0, T_0 \right) {\setminus } \left\{ \left( 0,0 \right) \right\} \right)$, then $\left| u\right| ^2 \left( 2dt \right) ^{\frac{d n}{2} -1 -\tau _n}$ satisfies the hypotheses of Lemma 3.5 and it follows that

19 $$\begin{aligned} \begin{aligned}&\int _{B_{T_0}^n} \left| \sqrt{\kappa _n(y)} v_{n}\left( y \right) \right| ^2 \left| y\right| ^{-2 \tau _n} dy\\&\quad =\int _{B_{T_0}^n} \kappa _n(y) \left| v_{n}\left( y \right) \right| ^2 \left| y\right| ^{-2 \tau _n} dy \\&\quad = \int _{B_{T_0}^n} \kappa _n(y) \left| u\left( g \circ f_{d,n}(y), \frac{\left| y\right| ^2}{2d} \right) \right| ^2 \left| y\right| ^{d n - 2 -2 \tau _n} \left| y\right| ^{2 - d n} dy \\&\quad = d \left| S^{d n-1}\right| \int _0^{T_0} \int _{\mathbb {R}^d} \left| u\left( x, t \right) \right| ^2 \left( 2dt \right) ^{\frac{d n}{2} -1 -\tau _n} K_n\left( h(x),t \right) dx \, dt. \end{aligned} \end{aligned}$$

Because $\left\{ {{\,\textrm{div}\,}}\left( A \nabla u \right) + \frac{ \partial u}{ \partial t}+ \frac{2}{dn}\left[ \left\langle A\nabla \frac{ \partial u}{ \partial t}, H^T h \right\rangle + \frac{1}{2} \frac{ \partial u}{ \partial t} \text {tr}\left( H \left\langle \nabla _z G(h), h \right\rangle \right) + t \frac{ \partial ^2 u}{ \partial t^2} \right] \right\} ^2 \left( 2dt \right) ^{\frac{d n}{2} + 1- \tau _n}$ also satisfies the hypotheses of Lemma 3.5, then another application of Lemma 3.5 shows that

20 $$\begin{aligned} \begin{aligned}&\frac{1}{\left| S^{d n-1}\right| }\int _{B_{T_0}^n} \kappa _n \left[ \frac{1}{\kappa _n} {{\,\textrm{div}\,}}\left( \kappa _n \nabla v_n \right) \right] ^2 \left| y\right| ^{-2\tau _n +4} dy \\&\quad = \frac{1}{\left| S^{d n-1}\right| }\int _{B_{T_0}^n} \kappa _n\left( y \right) \left[ \frac{1}{\kappa _n} {{\,\textrm{div}\,}}\left( \kappa _n \nabla v_n \right) \right] ^2 \left| y\right| ^{d n + 2-2 \tau _n} \left| y\right| ^{2 - d n} dy \\&\quad \le 2 d n ^2 \int _0^{T_0} \int _{\mathbb {R}^d} \left| {{\,\textrm{div}\,}}\left( A \nabla u \right) + \frac{ \partial u}{ \partial t}\right| ^2 \left( 2dt \right) ^{\frac{d n}{2} + 1- \tau _n} K_n\left( h(x),t \right) dx \, dt \\&\qquad + \frac{8}{d} \int _0^{T_0} \int _{\mathbb {R}^d} \left| \left\langle A\nabla \frac{ \partial u}{ \partial t}, H^T h \right\rangle + \frac{1}{2} \frac{ \partial u}{ \partial t} \text {tr}\left( H \left\langle \nabla _z G(h), h \right\rangle \right) + t \frac{ \partial ^2 u}{ \partial t^2} \right| ^2\\&\qquad \times \left( 2dt \right) ^{\frac{d n}{2} + 1- \tau _n} K_n\left( h(x),t \right) dx \, dt, \end{aligned} \end{aligned}$$

where we have used (9) from Lemma 2.2 and the triangle inequality to reach the last line.

Finally, because $\left[ {2 d n t} \frac{{{\,\textrm{div}\,}}\left( A \nabla \sqrt{\eta } \right) }{\sqrt{\eta }}\right] ^2 \left| u\right| ^2 \left( 2dt \right) ^{\frac{d n}{2} -1 -\tau _n}$ satisfies the hypotheses of Lemma 3.5, we see that

21 $$\begin{aligned} \begin{aligned}&\int _{B_{T_0}^n} \left[ \frac{\left| y\right| ^2}{\sqrt{\kappa _n}} {{\,\textrm{div}\,}}\left( \kappa _n \nabla \left( \kappa _n^{-1/2} \right) \right) \right] ^2 \left| \sqrt{\kappa }_n v_n\right| ^2 \left| y\right| ^{-2\tau _n } dy \\&\quad = d \left| S^{d n-1}\right| \int _0^{T_0} \int _{\mathbb {R}^d} \left[ {2 d n t} \frac{{{\,\textrm{div}\,}}\left( A \nabla \sqrt{\eta } \right) }{\sqrt{\eta }}\right] ^2 \left| u\left( x, t \right) \right| ^2 \left( 2dt \right) ^{\frac{d n}{2} -1 -\tau _n} K_n\left( h(x),t \right) dx \, dt \\&\quad \le d n^2 \left| S^{d n-1}\right| \left\| \frac{{{\,\textrm{div}\,}}\left( A \nabla \sqrt{\eta } \right) }{\sqrt{\eta }}\right\| _{L^\infty \left( \mathbb {R}^d \right) }^2 \int _0^{T_0} \int _{\mathbb {R}^d} \left| u\left( x, t \right) \right| ^2 \left( 2dt \right) ^{\frac{d n}{2} +1 -\tau _n} K_n\left( h(x),t \right) dx \, dt, \end{aligned}\nonumber \\ \end{aligned}$$

since (9) with $v_n = \kappa _n^{-1/2}$ gives

$$\begin{aligned} \frac{\left| y\right| ^2}{\sqrt{\kappa _n(y)}} {{\,\textrm{div}\,}}_y \left( \kappa _n(y) \nabla _y \left( \kappa _n(y)^{-1/2} \right) \right)&= {2 d n t} \frac{{{\,\textrm{div}\,}}_x \left( A(x) \nabla _x \sqrt{\eta }(x) \right) }{\sqrt{\eta (x)}}. \end{aligned}$$

A computation shows that

$$\begin{aligned} \frac{{{\,\textrm{div}\,}}\left( A \nabla \sqrt{\eta } \right) }{\sqrt{\eta }}&= \frac{{{\,\textrm{div}\,}}\left[ H^{-1} \left( H^{-1} \right) ^T \sqrt{\eta }\, \nabla \log \eta \right] }{2 \sqrt{\eta }} \\&= \frac{1}{2} {{\,\textrm{div}\,}}\left[ H^{-1} \left( H^{-1} \right) ^T\nabla \log \eta \right] + \frac{1}{4} \left| \left( H^{-1} \right) ^T \nabla \log \eta \right| ^2, \end{aligned}$$

which belongs to $L^\infty \left( \mathbb {R}^d \right)$ by assumption. Substituting (19), (20), and (21) into (18) and simplifying shows that

22 $$\begin{aligned} \begin{aligned}&c\left( \tau _n, dn \right) ^2 \int _0^{T_0} \int _{\mathbb {R}^d} \left| u\left( x, t \right) \right| ^2 t^{\frac{d n}{2} -1 -\tau _n} K_n\left( h(x),t \right) dx \, dt \\&\quad \le 2 C_\delta \left( 2 d n \right) ^{2} \int _0^{T_0} \int _{\mathbb {R}^d} \left| {{\,\textrm{div}\,}}\left( A \nabla u \right) + \frac{ \partial u}{ \partial t}\right| ^2 t^{\frac{d n}{2} + 1- \tau _n} K_n\left( h(x),t \right) dx \, dt \\&\qquad + 32 C_\delta \int _0^{T_0} \int _{\mathbb {R}^d} \left[ \left\langle A\nabla \tfrac{ \partial u}{ \partial t}, H^T h \right\rangle + \tfrac{1}{2}\tfrac{ \partial u}{ \partial t} \text {tr}\left( H \left\langle \nabla _z G(h), h \right\rangle \right) + t \tfrac{ \partial ^2 u}{ \partial t^2} \right] ^2 t^{\frac{d n}{2} + 1- \tau _n} K_n\left( h(x),t \right) dx \, dt \\&\qquad + \left( d n \varepsilon \right) ^2 \left( 1 - \delta ^2 \right) \int _0^{T_0} \int _{\mathbb {R}^d} \left| u\left( x, t \right) \right| ^2 t^{\frac{d n}{2} -1 -\tau _n} K_n\left( h(x),t \right) dx \, dt, \end{aligned} \end{aligned}$$

where we have used the definition of $c_\delta$ and that $\displaystyle 4 T_0 \left\| \frac{{{\,\textrm{div}\,}}\left( A \nabla \sqrt{\eta } \right) }{\sqrt{\eta }}\right\| _{L^\infty \left( \mathbb {R}^d \right) } = \varepsilon \sqrt{1 - \delta }$ to reach the last line. Observe that

$$\begin{aligned} c\left( \tau _n, dn \right)&= \inf _{\ell \in \mathbb {Z}_{\ge 0}} \left| \left[ \frac{d n}{2} + \ell + \left( 2\alpha + \frac{d n - d - 2}{2} \right) - 2\right] \left[ \frac{d n}{2} + \ell - \left( 2\alpha + \frac{d n - d - 2}{2} \right) \right] \right| \\&= \inf _{\ell \in \mathbb {Z}_{\ge 0}} \left| \left[ d n -2 + \ell + \left( 2\alpha - \frac{ d }{2} - 1 \right) \right] \left[ \ell - \left( 2\alpha - \frac{ d }{2} - 1 \right) \right] \right| \ge d n \varepsilon , \end{aligned}$$

since we assumed that $2 \alpha - \frac{d}{2} - 3 > 0$. Returning to (22), the last term on the right may be absorbed into the left to get

$$\begin{aligned} \begin{aligned}&\int _0^{T_0} \int _{\mathbb {R}^d} \left| u\left( x, t \right) \right| ^2 t^{ \frac{d}{2} - 2\alpha } K_n\left( h(x),t \right) dx \, dt \\&\quad \le 2 C_\delta \left( \frac{2}{\varepsilon \delta } \right) ^{2} \int _0^{T_0} \int _{\mathbb {R}^d} \left| {{\,\textrm{div}\,}}\left( A \nabla u \right) + \frac{ \partial u}{ \partial t}\right| ^2 t^{\frac{d}{2} - 2\alpha + 2} K_n\left( h(x),t \right) dx \, dt \\&\qquad + 2 C_\delta \left( \frac{4}{dn \varepsilon \delta } \right) ^2 \int _0^{T_0} \int _{\mathbb {R}^d} \left[ \left\langle A\nabla \tfrac{ \partial u}{ \partial t}, H^T h \right\rangle + \tfrac{1}{2}\tfrac{ \partial u}{ \partial t} \text {tr}\left( H \left\langle \nabla _z G(h), h \right\rangle \right) + t \tfrac{ \partial ^2 u}{ \partial t^2} \right] ^2 t^{\frac{d}{2} - 2\alpha + 2} K_n\left( h(x),t \right) dx \, dt, \end{aligned} \end{aligned}$$

with $2\alpha = \tau _n - \frac{d n - d - 2}{2}$. We now take the limit as $n \rightarrow \infty$. An application of Lemma 3.1 shows that

$$\begin{aligned} \begin{aligned}&\int _0^{T_0} \int _{\mathbb {R}^d} \left| u\left( x, t \right) \right| ^2 t^{ - 2\alpha } \exp \left( - \frac{\left| h(x)\right| ^2}{4t} \right) dx \, dt \\&\quad \le \frac{8 \left( 1 + \delta \right) }{\varepsilon ^2 \delta ^2} \int _0^{T_0} \int _{\mathbb {R}^d} \left| {{\,\textrm{div}\,}}\left( A \nabla u \right) + \frac{ \partial u}{ \partial t}\right| ^2 t^{- 2\alpha + 2} \exp \left( - \frac{\left| h(x)\right| ^2}{4t} \right) dx \, dt, \end{aligned} \end{aligned}$$

as required. $$\square $$

## Almgren monotonicity formula

In this section, we show that the Almgren-type frequency function associated with the parabolic operator $\text {div}(A\nabla )+\partial _t$ is monotonically non-decreasing. When $A = I$, a corresponding result goes back to Poon [49]. In that paper, the monotonicity was key in the proof of unique continuation results for caloric functions. A version of this result was later proved in the context of the parabolic, constant-coefficient Signorini problem in [17], where the authors used the monotonicity to establish the optimal regularity of solutions and study the free boundary.

To establish our parabolic result through the high-dimensional limiting technique, we require a similar result for solutions to non-homogeneous variable-coefficient elliptic equations. Many similar results for the homogenous setting have been previously established. For example, Almgren-type monotonicity formulas for variable-coefficient operators have also been extensively used to study a wide variety of free boundary problems, as in [8, 20, 31–33, 35, 36]. The following result is crucial to our upcoming parabolic proof, but it may also be of independent interest.

### Proposition 5.1

For some $R > 0$, let $B_R \subset \mathbb {R}^N$. Assume that for $\kappa : B_R \rightarrow \mathbb {R}_{+}$ it holds that $\nabla \log \kappa \cdot y \in L^{\infty }\left( B_R \right)$. Let $v \in W^{1,2}\left( B_R, \kappa dy \right)$ be a weak solution to ${{\,\textrm{div}\,}}\left( \kappa \nabla v \right) = \kappa \ell$ in $B_R$, where $\ell$ is integrable with respect to both $\kappa \, v$ and $\kappa \nabla v \cdot y$ on each $B_r$, for $r \in \left( 0, R \right)$. For every $r \in \left( 0, R \right)$, assuming that each $v |_{ \partial B_r}$ is non-trivial, define

$$\begin{aligned} H(r)&= H(r; v, \kappa ) = \int _{ \partial B_r} \kappa \left( y \right) \left| v(y)\right| ^2 d\sigma (y) \\ D(r)&= D(r; v, \kappa ) = \int _{B_r} \kappa (y) \left| \nabla v(y)\right| ^2 dy \\ L(r)&= L(r; v, \kappa ) = \frac{r D(r; v, \kappa )}{H(r; v, \kappa )}. \end{aligned}$$

Set $\widetilde{L}(r) = r^{2\Upsilon } L(r)$, where $\Upsilon \ge \left\| \nabla \log \kappa \cdot y\right\| _{L^\infty (B_R)}$. Then for all $r \in \left( 0, R \right)$, it holds that

$$\begin{aligned} \widetilde{L}'(r)&\ge 2 r^{2\Upsilon } \left[ \frac{\left( \int _{B_r} \kappa \, \ell v \, dy \right) \left( \int _{ \partial B_r} \kappa \, v \, \nabla v \cdot y \, d\sigma (y) \right) }{ \left( \int _{ \partial B_r} \kappa \left| v\right| ^2 \, d\sigma (y) \right) ^2} - \frac{\left( \int _{B_r} \kappa \, \ell \, \nabla v \cdot y \, dy \right) }{\left( \int _{ \partial B_r} \kappa \left| v\right| ^2\, d\sigma (y) \right) }\right] . \end{aligned}$$

### Remark 5.2

Notice that if *v* is a solution to the homogeneous equation ${{\,\textrm{div}\,}}\left( \kappa \nabla v \right) = 0$ in $B_R$, i.e. $\ell = 0$, then $\widetilde{L}(r)$ is non-decreasing in *r*. Moreover, if $\kappa = 1$, we recover the non-homogenous elliptic result from [18, Corollary 1], which is the non-homogeneous version of Poon’s result [49]. In particular, we recover the expected monotonicity formula for solutions to elliptic equations.

### Proof

Observe first that since

$$\begin{aligned} H(r) = \int _{ \partial B_r} \kappa \left( y \right) \left| v(y)\right| ^2 d\sigma (y) = r^{N-1} \int _{ \partial B_1} \kappa \left( r\zeta \right) \left| v(r\zeta )\right| ^2 d\sigma (\zeta ), \end{aligned}$$

then

$$\begin{aligned} H'(r)&= \frac{N-1}{r} H(r) + 2 r^{N-1} \int _{ \partial B_1} \kappa \left( r\zeta \right) v(r\zeta ) \nabla v(r\zeta ) \cdot \zeta d\sigma (\zeta ) \\&\quad + r^{N-1}\int _{ \partial B_1} \nabla \kappa \left( r \zeta \right) \cdot \zeta \left| v(r\zeta )\right| ^2 d\sigma (\zeta ) \\&= \frac{N-1}{r} H(r) + 2 \int _{ \partial B_r} \kappa \, v \, \nabla v \cdot \hat{n} + \int _{ \partial B_r} \nabla \kappa \cdot \hat{n} \, \left| v\right| ^2 , \end{aligned}$$

where $\hat{n}$ indicates the outer unit normal. Moreover, integration by parts and the equation for *v* shows that

23 $$\begin{aligned} D(r) = \int _{B_r} \kappa \left| \nabla v\right| ^2 = \int _{B_r} \frac{1}{2} {{\,\textrm{div}\,}}\left[ \kappa \nabla \left( v^2 \right) \right] - {{\,\textrm{div}\,}}\left( \kappa \nabla v \right) v = \int _{ \partial B_r} \kappa v \nabla v \cdot \hat{n} - \int _{B_r} \kappa \ell v. \end{aligned}$$

Now differentiating and integrating by parts shows that

$$\begin{aligned} D'(r)&= \int _{ \partial B_r} \kappa \left| \nabla v\right| ^2 = \frac{1}{r} \int _{ \partial B_r} \kappa \left| \nabla v\right| ^2 y \cdot {\hat{n}} \, = \frac{1}{r} \int _{B_r} {{\,\textrm{div}\,}}\left( \kappa \left| \nabla v\right| ^2 y \right) \\&= \frac{N}{r} \int _{B_r} \kappa \left| \nabla v\right| ^2 + \frac{2}{r} \int _{B_r} \kappa \nabla v \cdot D^2 v \, y + \frac{1}{r} \int _{B_r} \nabla \kappa \cdot y \left| \nabla v\right| ^2 \\&= \frac{N}{r} \int _{B_r} \kappa \left| \nabla v\right| ^2 - \frac{2}{r} \int _{B_r} \kappa \left| \nabla v\right| ^2 \\&\quad - \frac{2}{r} \int _{B_r} {{\,\textrm{div}\,}}\left( \kappa \nabla v \right) \nabla v \cdot y + \frac{2}{r^2} \int _{ \partial B_r} \kappa \left( \nabla v \cdot y \right) ^2 + \frac{1}{r} \int _{B_r} \nabla \kappa \cdot y \left| \nabla v\right| ^2 \\&= \frac{N-2}{r} D(r) + 2 \int _{ \partial B_r} \kappa \left( \nabla v \cdot \hat{n} \right) ^2 + \int _{B_r} \nabla \kappa \cdot \frac{y}{r} \left| \nabla v\right| ^2 - 2 \int _{B_r} \kappa \, \ell \, \nabla v \cdot \frac{y}{r}. \end{aligned}$$

Therefore, by putting it all together, we see that

$$\begin{aligned} H(r)^2 L'(r)&= D(r)H(r) + r D'(r) H(r) - r D(r) H'(r) \\&= D(r)H(r) \\&\quad + \left[ \left( N-2 \right) D(r)+ 2r \int _{ \partial B_r} \kappa \left( \nabla v \cdot \hat{n} \right) ^2 +r \int _{B_r} \nabla \kappa \cdot \frac{y}{r} \left| \nabla v\right| ^2 - 2r \int _{B_r} \kappa \, \ell \, \nabla v \cdot \frac{y}{r}\right] H(r) \\&\quad - D(r) \left[ \left( N-1 \right) H(r) + 2r \int _{ \partial B_r} \kappa v \nabla v \cdot \hat{n} + r \int _{ \partial B_r} \nabla \kappa \cdot \hat{n} \left| v\right| ^2 \right] \\&= r \left( 2 \int _{ \partial B_r} \kappa \left( \nabla v \cdot \hat{n} \right) ^2 + \int _{B_r} \nabla \kappa \cdot \frac{y}{r} \left| \nabla v\right| ^2 - 2 \int _{B_r} \kappa \, \ell \, \nabla v \cdot \frac{y}{r} \right) \left( \int _{ \partial B_r} \kappa \left| v\right| ^2 \right) \\&\quad - 2r \left( \int _{ \partial B_r} \kappa v \nabla v \cdot \hat{n} - \int _{B_r}\kappa \, \ell v \right) \left( \int _{ \partial B_r} \kappa v \nabla v \cdot \hat{n} \right) \\&\quad - r \left( \int _{B_r} \kappa \left| \nabla v\right| ^2 \right) \left( \int _{ \partial B_r} \nabla \kappa \cdot \hat{n} \left| v\right| ^2 \right) \\&= -\frac{2}{r} \left[ \left( \int _{ \partial B_r} \kappa \, v \nabla v \cdot y \right) ^2 - \left( \int _{ \partial B_r} \kappa \left| v\right| ^2 \right) \left( \int _{ \partial B_r} \kappa \left( \nabla v \cdot y \right) ^2 \right) \right] \\&\quad - \left[ \left( \int _{B_r} \kappa \left| \nabla v\right| ^2 \right) \left( \int _{ \partial B_r} \frac{\nabla \kappa \cdot y}{\kappa } \kappa \left| v\right| ^2 \right) - \left( \int _{B_r} \frac{\nabla \kappa \cdot y}{\kappa } \kappa \left| \nabla v\right| ^2 \right) \left( \int _{ \partial B_r} \kappa \left| v\right| ^2 \right) \right] \\&\quad + 2 \left[ \left( \int _{B_r} \kappa \, \ell v \right) \left( \int _{ \partial B_r} \kappa \, v \, \nabla v \cdot y \right) - \left( \int _{B_r} \kappa \, \ell \, \nabla v \cdot y \right) \left( \int _{ \partial B_r} \kappa \left| v\right| ^2 \right) \right] . \end{aligned}$$

An application of Cauchy–Schwartz shows that

$$\begin{aligned} \left( \int _{ \partial B_r} \kappa v \nabla v \cdot y \right) ^2&\le \left( \int _{ \partial B_r} \kappa \left| v\right| ^2 \right) \left( \int _{ \partial B_r} \kappa \left( \nabla v \cdot y \right) ^2 \right) \end{aligned}$$

while with $\Upsilon \ge \left\| \nabla \log \kappa \cdot y\right\| _{L^\infty (B_R)}$, we get that

$$\begin{aligned}&\left| \left( \int _{B_r} \kappa \left| \nabla v\right| ^2 \right) \left( \int _{ \partial B_r} \frac{\nabla \kappa \cdot y}{\kappa } \kappa \left| v\right| ^2 \right) - \left( \int _{B_r} \frac{\nabla \kappa \cdot y}{\kappa } \kappa \left| \nabla v\right| ^2 \right) \left( \int _{ \partial B_r} \kappa \left| v\right| ^2 \right) \right| \le 2 \Upsilon D\left( r \right) H\left( r \right) . \end{aligned}$$

It follows that

$$\begin{aligned} L'(r)&\ge - \frac{2\Upsilon }{r} \frac{r D\left( r \right) }{ H\left( r \right) } + 2 \left[ \frac{\left( \int _{B_r} \kappa \, \ell v \right) \left( \int _{ \partial B_r} \kappa v \nabla v \cdot y \right) }{ \left( \int _{ \partial B_r} \kappa \left| v\right| ^2 \right) ^2} - \frac{\left( \int _{B_r} \kappa \, \ell \, \nabla v \cdot y \right) }{\left( \int _{ \partial B_r} \kappa \left| v\right| ^2 \right) }\right] , \end{aligned}$$

or rather,

$$\begin{aligned}&\left( r^{2\Upsilon } L(r) \right) ' = r^{2\Upsilon } L'(r) + \frac{2\Upsilon }{r} r^{2\Upsilon } L(r) \\&\quad \ge 2r^{2\Upsilon } \left[ \frac{\left( \int _{B_r} \kappa \, \ell v \right) \left( \int _{ \partial B_r} \kappa v \nabla v \cdot y \right) }{ \left( \int _{ \partial B_r} \kappa \left| v\right| ^2 \right) ^2} - \frac{\left( \int _{B_r} \kappa \, \ell \, \nabla v \cdot y \right) }{\left( \int _{ \partial B_r} \kappa \left| v\right| ^2 \right) }\right] . \end{aligned}$$

$$\square $$

Now we use Lemma 3.3 in combination with Proposition 5.1 to establish its parabolic counterpart. Before stating the result, we discuss the kinds of solutions that we work with.

Let $u: \mathbb {R}^d \times \left( 0, T \right) \rightarrow \mathbb {R}$ have moderate *h*-growth at infinity, as described in Definition 3.8. For every $t \in \left( 0, T \right)$, assume first that *u* is sufficiently regular to define the functionals

24 $$\begin{aligned} \begin{aligned} \mathcal {I}(t)&= \mathcal {I}(t; u,h) = \int _{\mathbb {R}^d} \left| u\left( x,t \right) \right| \left| \left\langle A\nabla u, {H^T h} \right\rangle + 2 t \, \partial _t u\right| \exp \left( -\frac{\left| h(x)\right| ^2}{4t} \right) dx \\ \mathcal {J}(t)&= \mathcal {J}(t; u,h) = \int _{\mathbb {R}^d} \left| J(x,t)\right| \left| u\left( x,t \right) \right| \exp \left( -\frac{\left| h(x)\right| ^2}{4t} \right) dx \\ \mathcal {K}(t)&= \mathcal {K}(t; u,h) = \int _{\mathbb {R}^d} \left| J(x,t)\right| \left| \left\langle A\nabla u, {H^T h} \right\rangle + 2 t \, \partial _t u\right| \exp \left( -\frac{\left| h(x)\right| ^2}{4t} \right) dx, \end{aligned} \end{aligned}$$

where

25 $$\begin{aligned} J(x,t) = J(x, t; u,h) = \frac{1}{d} \left[ 2 \left\langle A\nabla \frac{ \partial u}{ \partial t}, H^T h \right\rangle + \frac{ \partial u}{ \partial t} \text {tr}\left( H \left\langle \nabla _z G(h), h \right\rangle \right) + 2t \frac{ \partial ^2 u}{ \partial t^2} \right] \nonumber \\ \end{aligned}$$

and all derivatives are interpreted in the weak sense. Then we say that such a function *u* belongs to the function class $\mathfrak {A}\left( \mathbb {R}^d \times \left( 0, T \right) , h \right)$ if *u* has moderate *h*-growth at infinity (so is consequently continuous), and for every $t_0 \in \left( 0, T \right)$, there exists $\varepsilon \in \left( 0, t \right)$ so that

26 $$\begin{aligned} \mathcal {I} \in L^\infty \left[ t_0 - \varepsilon , t_0\right] \end{aligned}$$

and there exists $p > 1$ so that

27 $$\begin{aligned} \mathcal {J} \in L^{p}\left( \left[ 0, t_0\right] , t^{- \frac{d}{2}} dt \right) , \quad \mathcal {K} \in L^{p}\left( \left[ 0, t_0\right] , t^{- \frac{d}{2}} dt \right) . \end{aligned}$$

This is the class of functions that we consider in our result. We remark that this may not be the weakest set of conditions under which our proof holds, but it is far less restrictive than assuming that our solutions are smooth and compactly supported, for example.

### Theorem 5.3

Assume that $\text {tr}\left( H \left\langle \nabla _z G(h),h \right\rangle \right) \in L^\infty (\mathbb {R}^d)$, where *h*, *H*, and *G* are described by (4), (2), and (5). Define $A = H^{-1}\left( H^{-1} \right) ^T: \mathbb {R}^d \rightarrow \mathbb {R}^{d \times d}$ and let $u \in \mathfrak {A}\left( \mathbb {R}^d \times \left( 0, T \right) , h \right)$ be a non-trivial solution to ${{\,\textrm{div}\,}}\left( A \nabla u \right) + \partial _t u = 0$ in $\mathbb {R}^d \times \left( 0, T \right)$. For every $t \in \left( 0, T \right)$, define

$$\begin{aligned} \mathcal {H}\left( t \right) = \mathcal {H}\left( t; u,h \right)&= \int _{\mathbb {R}^d} \left| u(x,t)\right| ^2 e^{-\frac{\left| h(x)\right| ^2}{4t}} dx \\ \mathcal {D}\left( t \right) = \mathcal {D}\left( t; u,h \right)&= \int _{\mathbb {R}^d} \left\langle A(x) \nabla u(x,t), \nabla u(x,t) \right\rangle e^{-\frac{\left| h(x)\right| ^2}{4t}} dx \\ \mathcal {L}\left( t \right) = \mathcal {L}\left( t; u,h \right)&= \frac{t \mathcal {D}\left( t; u, h \right) }{\mathcal {H}\left( t; u, h \right) }. \end{aligned}$$

Set $\widetilde{\mathcal {L}}\left( t \right) = t^{\Upsilon } \mathcal {L}\left( t \right)$, where $\Upsilon \ge \left\| \text {tr}\left( H \left\langle \nabla _z G(h), h \right\rangle \right) \right\| _{L^\infty ( \mathbb {R}^d)}$. Then $\widetilde{\mathcal {L}}\left( t \right)$ is monotonically non-decreasing in *t*.

### Proof

We first check that with $\kappa _n$ and $v_n$ defined through the transformation maps $F_{d,n}$, the hypothesis of Lemma 5.1 are satisfied. Recall that $B_T^n \subset \mathbb {R}^{d \times n}$ is given by (15). Let $\kappa _n$ be as in Lemma 2.1, which shows that

$$\begin{aligned} \left\| \nabla \log \kappa _n \cdot y\right\| _{L^\infty (B_T^n)} \le \left\| \text {tr}\left( H \left\langle \nabla _z G(h),h \right\rangle \right) \right\| _{L^\infty ({\mathbb {R}^d})} < \infty . \end{aligned}$$

In particular, $\nabla \log \kappa _n \cdot y \in L^\infty (B_T^n)$ for each *n*.

Let $u \in \mathfrak {A}\left( \mathbb {R}^{d} \times \left( 0,T \right) , h \right)$ be a non-trivial solution to $\displaystyle {{\,\textrm{div}\,}}\left( A \nabla u \right) + \partial _t u = 0$ in $\mathbb {R}^d \times \left( 0,T \right)$. For every $n \in \mathbb {N}_{\ge 2}$, let $v_n: B_{T}^n \rightarrow \mathbb {R}$ satisfy

$$\begin{aligned} v_n\left( y \right) = u\left( F_{d,n}\left( y \right) \right) . \end{aligned}$$

Since *u* has moderate *h*-growth at infinity, then Lemma 3.9 shows that each $v_n$ belongs to $W^{1,2}\left( B_T^n, \kappa _n(y) \, dy \right)$.

An application of Corollary 2.3 shows that

$$\begin{aligned} \frac{1}{\kappa _n(y)} {{\,\textrm{div}\,}}\left( \kappa _n(y)\nabla v_n \right)&= J\left( x,t \right) , \end{aligned}$$

where *J* is defined in (25) and does not depend on *n*. For every *n*, define $\ell _{n}: B_{T}^n \rightarrow \mathbb {R}$ so that

$$\begin{aligned} \ell _n\left( y \right) = J\left( F_{d,n}\left( y \right) \right) \end{aligned}$$

and then

$$\begin{aligned} {{\,\textrm{div}\,}}\left( \kappa _n \nabla v_n \right) = \kappa _n \ell _n. \end{aligned}$$

Since $u \in \mathfrak {A}\left( \mathbb {R}^{d} \times \left( 0,T \right) , h \right)$, then (27) implies that for every $t \in \left( 0, T \right)$, $\mathcal {J}$ belongs to $L^{1}\left( \left[ 0, t\right] , s^{- \frac{d}{2}} ds \right)$. Corollary 3.7 then shows that $\ell _{n} v_n$ is integrable with respect to $\kappa _n \, dy$ in each $B_t^n$. Similarly, because $\mathcal {K}$ belongs to $L^{1}\left( \left[ 0, t\right] , s^{- \frac{d}{2}} ds \right)$, then Lemma 2.2 and Corollary 3.7 show that $\ell _{n} \nabla v_n \cdot y$ is integrable with respect to $\kappa _n \, dy$ in each $B_t^n$. Rephrased, this means that $\ell _n$ is integrable with respect to both $\kappa _n \, v_n$ and $\kappa _n \, \nabla v_n \cdot y$ on each $B_t^n$.

By backward uniqueness of heat equations, (as in [46], for example), $u\left( \cdot , t \right)$ is a non-trivial function of *x* for each $t \in \left( 0, T \right)$.

Since $v_n |_{ \partial B_r} = u \left( \cdot , \frac{r^2}{2d} \right)$ is non-trivial for each $r \in \left( 0, \sqrt{2dT} \right)$, then all of the assumptions from Proposition 5.1 hold. Therefore, we may apply Proposition 5.1 to each $v_n$ on any ball of radius $\sqrt{2 d t}$ for $t < T$.

First we compute the frequency function associated to $v_n$ on the ball of radius $\sqrt{2 d t}$. By Lemma 3.3,

28 $$\begin{aligned} H\left( \sqrt{2 d t}; v_n, \kappa _n \right)&= \int _{S_t^n} \kappa _n(y) \left| v_n\left( y \right) \right| ^2 d\sigma (y) \nonumber \\&\quad = \left( 2 d t \right) ^{\frac{d n -1}{2}} \left| S^{d n -1}\right| \int _{\mathbb {R}^d} \left| u\left( x,t \right) \right| ^2 K_{t,n}\left( h\left( x \right) \right) d{x}. \end{aligned}$$

Lemmas 2.2 and 3.3 imply that

29 $$\begin{aligned} \begin{aligned}&\frac{\left( 2 d t \right) ^{ -\frac{d n -1}{2}}}{\left| S^{d n -1}\right| }\int _{S_t^n} \kappa _n(y) v_n\left( y \right) \left( y \cdot \nabla v_n\left( y \right) \right) d\sigma (y) \\ \\&\quad = \int _{\mathbb {R}^d} \left[ \left\langle A\nabla u, {H^T h} \right\rangle + 2 t \partial _t u\right] u\left( x,t \right) K_{t,n}\left( h\left( x \right) \right) d{x}, \end{aligned} \end{aligned}$$

while Lemma 3.5 implies that

30 $$\begin{aligned} \begin{aligned}&\frac{1}{\left| S^{d n -1}\right| }\int _{B_t^n} \kappa _n(y) \ell _n\left( y \right) v_n\left( y \right) \left| y\right| ^{dn -2} \left| y\right| ^{2- d n } d{y} \\&\quad = d \int _0^t \int _{\mathbb {R}^d} J(x,s) u\left( x,s \right) \left( 2 d s \right) ^{\frac{d n -2}{2}} K_{n}\left( h\left( x \right) , s \right) dx \, ds. \end{aligned} \end{aligned}$$

Using the expression (23) along with (29) and (30), we see that

$$\begin{aligned} \begin{aligned} \frac{1}{\left| S^{d n -1}\right| } D\left( \sqrt{2 d t}; v_n, \kappa _n \right)&= \left( 2 d t \right) ^{ \frac{d n -2}{2}}\int _{\mathbb {R}^d} \left[ \left\langle A\nabla u, {H^T h} \right\rangle + 2 t \partial _t u\right] u\left( x,t \right) K_{t,n}\left( h\left( x \right) \right) d{x} \\&\quad - d \int _0^t \int _{\mathbb {R}^d} J(x,s) u\left( x,s \right) \left( 2 d s \right) ^{\frac{d n -2}{2}} K_{n}\left( h\left( x \right) , s \right) dx \, ds. \end{aligned} \end{aligned}$$

We remark that since $u \in \mathfrak {A}\left( \mathbb {R}^d \times \left( 0, T \right) \right)$, then Lemmas 3.3 and 3.5 guarantee that the integrals in (28), (29), and (30) are all well-defined. Therefore,

$$\begin{aligned} L_n(t)&:=\frac{1}{2} L\left( \sqrt{2 d t}; v_n, \kappa _n \right) = \frac{\sqrt{2 d t} D\left( \sqrt{2 d t}; v_n, \kappa _n \right) }{2 H\left( \sqrt{2 d t}; v_n, \kappa _n \right) } \\&= \frac{ \int _{\mathbb {R}^d} u\left( x,t \right) \left[ \left\langle A\nabla u, {H^T h} \right\rangle + 2 t \partial _t u\right] K_{t,n}\left( h\left( x \right) \right) d{x} - d\int _0^t \int _{\mathbb {R}^d} J(x,s) u\left( x,s \right) \left( \frac{s}{t} \right) ^{\frac{d n -2}{2}} K_{n}\left( h\left( x \right) , s \right) dx \, ds}{ 2\int _{\mathbb {R}^d} \left| u\left( x,t \right) \right| ^2 K_{t,n}\left( h\left( x \right) \right) d{x}} \\&= \frac{ \int _{\mathbb {R}^d} u\left( x,t \right) \left[ \left\langle A\nabla u, {H^T h} \right\rangle + 2 t \partial _t u\right] K_{t,n}(h(x)) d{x} }{ 2\int _{\mathbb {R}^d} \left| u\left( x,t \right) \right| ^2 K_{t,n}(h(x)) d{x}} \\&\quad - \frac{ d\int _0^t \int _{\mathbb {R}^d} J(x,s) u\left( x,s \right) \left( \frac{s}{t} - \frac{\left| h(x)\right| ^2}{2 d n t} \right) ^{\frac{d n - d - 2}{2}}\chi _{B_{ns}}(h(x)) dx \, ds}{2\int _{\mathbb {R}^d} \left| u\left( x,t \right) \right| ^2 \left( 1 - \frac{\left| h(x)\right| ^2}{2 d n t} \right) ^{\frac{d n - d - 2}{2}}\chi _{B_{nt}}(h(x)) d{x}}. \end{aligned}$$

Thus,

$$\begin{aligned} \lim _{n \rightarrow \infty } L_n(t)&= \frac{ \int _{\mathbb {R}^d} u\left( x,t \right) \left[ \left\langle A\nabla u, {H^T h} \right\rangle + 2 t \partial _t u\right] K_{t}\left( h\left( x \right) \right) d{x} }{ 2\int _{\mathbb {R}^d} \left| u\left( x,t \right) \right| ^2 K_{t}\left( h\left( x \right) \right) d{x}} , \end{aligned}$$

where we use $\displaystyle \lim _{n \rightarrow \infty } K_{t,n}(x)= K_t(x)$ from (13), the bound (14), and that

$$\begin{aligned} \lim _{n \rightarrow \infty } \left| \left( \frac{s}{t} - \frac{\left| h(x)\right| ^2}{2 d n t} \right) ^{\frac{d n - d - 2}{2}}\chi _{B_{ns}}(h(x))\right| \le \lim _{n \rightarrow \infty }\left( \frac{s}{t} \right) ^{\frac{d n - d - 2}{2}} = 0 \end{aligned}$$

for every $s \in \left( 0, t \right)$ along with the Dominated Convergence Theorem.

Since $\partial _t u = - {{\,\textrm{div}\,}}\left( A \nabla u \right)$ and $\nabla \left( K_t \circ h \right) = - \frac{1}{2t} H^T h \, K_t\left( h \right)$, then

$$\begin{aligned}&\frac{1}{2} \int _{\mathbb {R}^d} u\left( x,t \right) \left[ \left\langle A\nabla u, {H^T h} \right\rangle + 2 t \partial _t u\right] K_{t}\left( h\left( x \right) \right) d{x} \\&\quad = -t \int _{\mathbb {R}^d} u\left( x,t \right) \left[ \left\langle A\nabla u, \nabla K_{t}\left( h(x) \right) \right\rangle + {{\,\textrm{div}\,}}\left( A \nabla u \right) K_{t}\left( h(x) \right) \right] d{x} \\&\quad = -t \int _{\mathbb {R}^d} u\left( x,t \right) {{\,\textrm{div}\,}}\left[ A\nabla u \, K_{t}\left( h(x) \right) \right] d{x} = t \int _{\mathbb {R}^d} \left\langle A\nabla u, \nabla u \right\rangle K_{t}\left( h(x) \right) d{x} \end{aligned}$$

and we deduce that

31 $$\begin{aligned} \lim _{n \rightarrow \infty } L_n(t)&= \mathcal {L}\left( t; u, h \right) . \end{aligned}$$

Define $\widetilde{L}_n(t) = t^{\Upsilon _n} L_n(t)$ for some $\Upsilon _n \ge \left\| \nabla \log \kappa _n \cdot y\right\| _{L^\infty (B_{T}^n)}$. Lemma 2.1 shows that

$$\begin{aligned} \left\| \nabla \log \kappa _n \cdot y\right\| _{L^\infty (B_T^n)} \le \left\| \text {tr}\left( H \left\langle \nabla _z G(h), h \right\rangle \right) \right\| _{L^\infty ({\mathbb {R}^d})} =: \Upsilon , \end{aligned}$$

so we may choose $\Upsilon _n = \Upsilon$ for all $n \in \mathbb {N}$. Observe that

$$\begin{aligned} \widetilde{L}_n(t)&= t^{\Upsilon } L_n(t) = \frac{1}{2} t^{\Upsilon } L\left( \sqrt{2 d t}; v_n, \kappa _n \right) \\&= \frac{1}{2 \left( 2d \right) ^{\Upsilon }} \left( 2dt \right) ^{\Upsilon } L\left( \sqrt{2 d t}; v_n, \kappa _n \right) = \frac{1}{2 \left( 2d \right) ^{\Upsilon }} \widetilde{L}\left( \sqrt{2 d t}; v_n, \kappa _n \right) , \end{aligned}$$

where ${\widetilde{L}}$ is as given in Proposition 5.1. An application of the chain rule shows that

$$\begin{aligned} \frac{d}{dt}\widetilde{L}_n(t)&= \frac{1}{2 \left( 2d \right) ^{\Upsilon }} \widetilde{L}'\left( \sqrt{2 d t}; v_n, \kappa _n \right) \frac{\sqrt{2d}}{2 \sqrt{t}}. \end{aligned}$$

By Proposition 5.1, it follows that

$$\begin{aligned} \frac{d}{dt}\widetilde{L}_n(t)&\ge t^{\Upsilon } \sqrt{\frac{d}{2t}} \left[ \frac{\left( \int _{S_t^n} \kappa _n \, v_n \,{ y \cdot \nabla v_n} \right) \left( \int _{B_t^n} \kappa _n \, \ell _n \, v_n \right) }{ \left( \int _{S_t^n} \kappa _n \, \left| v_n\right| ^2 \right) ^2 } - \frac{ \int _{B_t^n} \kappa _n \, \ell _n \, { y \cdot \nabla v_n }}{ \int _{S_t^n} \kappa _n \, \left| v_n\right| ^2}\right] . \end{aligned}$$

By Lemmas 2.2 and 3.5

$$\begin{aligned} \begin{aligned}&\frac{1}{d \left| S^{d n -1}\right| } \int _{B_t^n} \kappa _n(y) \, \ell _n\left( y \right) \left( y \cdot \nabla v_n\left( y \right) \right) \left| y\right| ^{d n -2} \left| y\right| ^{2 - d n} d{y} \\&\quad = \int _0^t \int _{\mathbb {R}^d} J(x,s) \left[ \left\langle A\nabla u, {H^T h} \right\rangle + 2 s \, \partial _s u\right] \left( 2 d s \right) ^{\frac{d n -2}{2}} K_{n}\left( h(x),s \right) d{x} \, d{s}. \end{aligned} \end{aligned}$$

Therefore, substituting this along with the expressions from (28), (29) and (30) into the previous inequality shows that

$$\begin{aligned}&\frac{d}{dt}\widetilde{L}_n(t) \ge - d t^{\Upsilon -1} \frac{ \int _0^t \left( \frac{s}{t} \right) ^{\frac{d n -2}{2}} \int _{\mathbb {R}^d} J(x,s) \left[ \left\langle A\nabla u, {H^T h} \right\rangle + 2 s \, \partial _s u\right] K_{n}\left( h(x),s \right) d{x} \, d{s} }{2 \int _{\mathbb {R}^d} \left| u\left( x,t \right) \right| ^2 K_{t,n}\left( h\left( x \right) \right) d{x}} \\&\quad + d t^{\Upsilon -1} \frac{\left( \int _{\mathbb {R}^d} u\left( x,t \right) \left[ \left\langle A\nabla u, {H^T h} \right\rangle + 2 t \, \partial _t u\right] K_{t,n}\left( h\left( x \right) \right) d{x} \right) \left( \int _0^t \left( \frac{s}{t} \right) ^{\frac{d n -2}{2}} \int _{\mathbb {R}^d}J(x,s) u\left( x,s \right) K_{n}\left( h\left( x \right) , s \right) dx \, ds \right) }{2 \left( \int _{\mathbb {R}^d} \left| u\left( x,t \right) \right| ^2 K_{t,n}\left( h\left( x \right) \right) d{x} \right) ^2 }. \end{aligned}$$

Estimate (14) along with (51) and (52) show that

32 $$\begin{aligned} \frac{d}{dt}\widetilde{L}_n(t)&\ge - \frac{d \mathcal {C}_{d}t^{\Upsilon -1}}{2 \alpha _{d} \mathcal {H}_n\left( t \right) } \left[ \int _0^t \left( \frac{s}{t} \right) ^{\frac{d n -2}{2}} \mathcal {K}(s) \, d{s} + \frac{\mathcal {C}_d \mathcal {I}(t)}{\alpha _d \mathcal {H}_n(t)} \int _0^t \left( \frac{s}{t} \right) ^{\frac{d n -2}{2}} \mathcal {J}(s) \, d{s}\right] =: {\widetilde{F}}_n(t) , \end{aligned}$$

where $\mathcal {I}$, $\mathcal {J}$, and $\mathcal {K}$ are defined in (24) and we have introduced

33 $$\begin{aligned} \mathcal {H}_n\left( t \right) = \mathcal {H}_n\left( t; u, h \right)&:= \int _{\mathbb {R}^d} \left| u(x,t)\right| ^2 \left( 1 - \frac{\left| h(x)\right| ^2}{2 d n t} \right) ^{\frac{d n - d - 2}{2}}\chi _{B_{nt}}(h(x)) dx. \end{aligned}$$

To show that $\widetilde{\mathcal {L}}$ is monotone non-decreasing, it suffices to show that given any $t_0 \in (0, T]$, there exists $\delta \in \left( 0, t_0 \right)$ so that $\widetilde{F}_n$ converges uniformly to 0 on $\left[ t_0 - \delta , t_0\right]$. Indeed, since $\frac{d}{dt}\widetilde{L}_n(t) \ge \widetilde{F}_n(t)$, then for any $t \in \left[ t_0 - \delta , t_0\right]$, it holds that

$$\begin{aligned} \widetilde{L}_n(t_0)-\widetilde{L}_n(t)\ge \int _{t}^{t_0}\widetilde{F}_n(s)ds. \end{aligned}$$

By definition and (31), $\widetilde{L}_n(t) = t^\Upsilon L_n(t)$ converges pointwise to $\widetilde{\mathcal {L}}(t)=t^{\Upsilon }\mathcal {L}(t;u, h)$, from which it follows that

$$\begin{aligned} \widetilde{\mathcal {L}}(t_0) - \widetilde{\mathcal {L}}(t) = \lim _{n \rightarrow \infty } \left[ \widetilde{L}_n(t_0)-\widetilde{L}_n(t)\right] \ge \lim _{n \rightarrow \infty } \int _{t}^{t_0}\widetilde{F}_n(s)ds. \end{aligned}$$

Assuming the local uniform convergence of ${\widetilde{F}}_n$ to 0 on $\left[ t_0 - \delta , t_0\right] \supset \left[ t, t_0\right]$, we see that

$$\begin{aligned} \lim _{n \rightarrow \infty } \int _{t}^{t_0}\widetilde{F}_n(s)ds = \int _{t}^{t_0} \lim _{n \rightarrow \infty } \widetilde{F}_n(s)ds = 0 \end{aligned}$$

and we may conclude that $\widetilde{\mathcal {L}}(t_0)-\widetilde{\mathcal {L}}(t) \ge 0$, as desired.

It remains to justify the local uniform convergence of $\widetilde{F}_n$ to 0, as described above. Let $t_0 \in (0, T]$ and recall that since *u* is non-trivial, then backward uniqueness ensures that $\displaystyle \int _{\mathbb {R}^d} \left| u\left( x,t_0 \right) \right| ^2 dx > 0$ so that $\mathcal {L}\left( t_0 \right)$ is well-defined.

We first consider the terms in the denominator of ${\widetilde{F}}_n$, defined as $\mathcal {H}_n\left( t \right)$ above. Observe that for any $n \in \mathbb {N}$, we have from (33) that

34 $$\begin{aligned} \begin{aligned} \mathcal {H}_n\left( t \right)&\ge \int _{\left\{ \left| h(x)\right| ^2 \le dnt\right\} } \left| u\left( x,t \right) \right| ^2 \left( 1 - \frac{\left| h(x)\right| ^2}{2 d n t} \right) ^{\frac{d n - d - 2}{2}} d{x} \\&\ge \int _{\left\{ \left| h(x)\right| ^2 \le dnt\right\} } \left| u\left( x,t \right) \right| ^2 \exp \left( - \frac{\ln 2\left| h(x)\right| ^2}{2 t} \right) d{x}, \end{aligned} \end{aligned}$$

where have used a Taylor expansion to show that if $\left| h(x)\right| ^2 \le dnt$, then

$$\begin{aligned}&\log \left[ \left( 1 - \frac{\left| h(x)\right| ^2}{2 d n t} \right) ^{\frac{d n - d - 2}{2}}\right] \\&\quad = - \frac{d n - d - 2}{dn} \frac{\left| h(x)\right| ^2}{4 t} \left[ 1 + \frac{1}{2} \left( \frac{\left| h(x)\right| ^2}{2 d n t} \right) + \frac{1}{3} \left( \frac{\left| h(x)\right| ^2}{2 d n t} \right) ^2 + \ldots \right] \\&\quad \ge - \frac{\left| h(x)\right| ^2}{4 t} \left[ 1 + \frac{1}{2} \left( \frac{1}{2} \right) + \frac{1}{3} \left( \frac{1}{2} \right) ^2 + \ldots \right] = - \frac{\ln 2 \left| h(x)\right| ^2}{2 t} . \end{aligned}$$

Set $K_m = \left\{ x: \left| h(x)\right| \le m\right\}$ and note that each $K_m$ is compact, the sets are nested, and $\mathbb {R}^d = \bigcup _{m \in \mathbb {N}} K_m$. Thus, for any positive real number $\displaystyle 2\,H \le \int _{\mathbb {R}^d} \left| u\left( x,t_0 \right) \right| ^2 dx$, there exists $M \in \mathbb {N}$ so that

$$\begin{aligned} \int _{K_M} \left| u\left( t_0 \right) \right| ^2 d{x} \ge \frac{3}{2} H. \end{aligned}$$

Fix some $\mu < \min \left\{ \frac{t_0}{2}, T - t_0\right\}$. Since *u* is continuous and $K_M \times \left[ t_1 - \mu , t_1 + \mu \right]$ is compact, then there exists $\delta \in (0, \mu ]$ so that whenever $x \in K_M$ and $\left| t - t_0\right| \le \delta$, it holds that

$$\begin{aligned} \left| u(x, t) - u(x, t_0)\right| < \sqrt{\frac{H}{2\left| K_M\right| }}. \end{aligned}$$

In particular, if $t \in \left[ t_0 - \delta , t_0\right]$, then

$$\begin{aligned} \int _{K_M} \left| u\left( x,t \right) \right| ^2 d{x}&\ge \int _{K_M} \left| u\left( x,t_0 \right) \right| ^2 d{x} - \int _{K_M} \left| u\left( x,t \right) - u\left( x,t_0 \right) \right| ^2 d{x} \ge H. \end{aligned}$$

If $N \in \mathbb {N}$ is large enough so that $d N \left( t_0 - \delta \right) \ge M^2$, then $\left\{ \left| h(x)\right| ^2 \le d N \left( t_0 - \delta \right) \right\} \supset K_M$. It follows that for any $n \ge N$ and any $t \in \left[ t_0 - \delta , t_0\right]$, we have from (34) that

35 $$\begin{aligned} \begin{aligned} \mathcal {H}_n\left( t \right)&\ge \int _{\left\{ \left| h(x)\right| ^2 \le dNt\right\} } \left| u\left( x,t \right) \right| ^2 \exp \left( - \frac{\ln 2\left| h(x)\right| ^2}{2 t} \right) d{x} \ge e^{- \frac{dN \ln 2 }{2} } \int _{K_M} \left| u\left( x,t \right) \right| ^2 d{x} \\&\ge H e^{- \frac{dN \ln 2 }{2} } > 0. \end{aligned} \end{aligned}$$

In particular, we have a uniform lower bound on all $\mathcal {H}_n\left( t \right)$ for $n \ge N$ and all $t \in \left[ t_0 - \delta , t_0\right]$.

Since $u \in \mathfrak {A}\left( \mathbb {R}^d \times \left( 0, T \right) , h \right)$, then there exists $p > 1$ so that (27) holds. Returning to the expression (32), an application of Hölder’s inequality shows that

$$\begin{aligned} \begin{aligned} \int _0^t \left( \frac{s}{t} \right) ^{\frac{d n -2}{2}} \mathcal {J}(s) \, d{s}&= \int _0^t \left( \frac{s}{t} \right) ^{\frac{d n + d -2}{2}} \left( \frac{s}{t} \right) ^{-\frac{d}{2}} \mathcal {J}(s) \, d{s}\\&\le \left[ \int _0^t \left( \frac{s}{t} \right) ^{\frac{d n + d -2}{2}\frac{p}{p-1}} d{s}\right] ^{\frac{p-1}{p}} \left\| \mathcal {J}\right\| _{L^{p}\left( \left[ 0, t\right] , s^{- \frac{d}{2}} ds \right) } \\&= t^{\frac{p-1}{p}} \left( \int _0^1 \tau ^{\frac{p\left( d n + d -2 \right) }{2\left( p-1 \right) }} d \tau \right) ^{\frac{p-1}{p}} \left\| \mathcal {J}\right\| _{L^{p}\left( \left[ 0, t\right] , s^{- \frac{d}{2}} ds \right) } \\&= \left[ \tfrac{2t\left( p-1 \right) }{n \left( d p + \frac{dp-2}{n} \right) } \right] ^{\frac{p-1}{p}} \left\| \mathcal {J}\right\| _{L^{p}\left( \left[ 0, t\right] , s^{- \frac{d}{2}} ds \right) }. \end{aligned} \end{aligned}$$

In particular,

36 $$\begin{aligned} \sup _{t \in \left[ t_0 - \delta , t_0\right] } \int _0^t \left( \frac{s}{t} \right) ^{\frac{d n -2}{2}} \mathcal {J}(s) \, d{s} \le c_{p,d} \left( \frac{t_0}{n} \right) ^{1 -\frac{1}{p}} \left\| \mathcal {J}\right\| _{L^{p}\left( \left[ 0, t\right] , s^{- \frac{d}{2}} ds \right) } \end{aligned}$$

and an identical argument holds with $\mathcal {K}$ in place of $\mathcal {J}$. Assuming that $\delta \le \varepsilon$ from (26) (which holds by possibly redefining $\delta$), we put (26), (35) and (36) together in (32) to see that whenever $n \ge N$,

$$\begin{aligned}&\inf _{t \in \left[ t_0 - \delta , t_0\right] } {\widetilde{F}}_n(t) \ge - \frac{ \mathcal {C}_{d,p}t_0^{\Upsilon -\frac{1}{p}}e^{ \frac{dN \ln 2 }{2} } }{2 \alpha _d H n^{1 - \frac{1}{p}}} \\&\quad \left[ \left\| \mathcal {K}\right\| _{L^{p}\left( \left[ 0, t_0\right] , t^{- \frac{d}{2}} dt \right) } + \frac{\mathcal {C}_d e^{\frac{dN \ln 2}{2}}}{H} \left\| \mathcal {I}\right\| _{L^\infty \left( \left[ t_0 - \delta ,t_0\right] \right) } \left\| \mathcal {J}\right\| _{L^{p}\left( \left[ 0, t_0\right] , t^{- \frac{d}{2}} dt \right) }\right. . \end{aligned}$$

The required version of uniform convergence follows from this bound and completes the proof. $\square$

## Alt–Caffarelli–Friedman monotonicity formula

In the groundbreaking work of Alt–Caffarelli–Friedman [2], the authors study two-phase elliptic free boundary problems. The monotonicity formula described in Proposition 6.1, which we refer to as ACF, is a crucial tool in their work since it is used to establish Lipschitz continuity of minimizers, and study the regularity of the free boundary.

### Proposition 6.1

([2], Lemma 5.1) For some $R > 0$, let $B_R \subset \mathbb {R}^N$. Let $v_1, v_2$ be two non-negative functions that belong to $C^0\left( B_R \right) \cap W^{1,2}\left( B_R \right)$. Assume that $\Delta v_1 \ge 0$ and $\Delta v_2 \ge 0$ in the sense of distributions, $v_1 v_2 \equiv 0$ and $v_1\left( 0 \right) = v_2\left( 0 \right) = 0$. Then for all $r < R$,

37 $$\begin{aligned} \phi \left( r; v \right) = \frac{1}{r^4} \left( \int _{B_r} \left| \nabla v_1\left( y \right) \right| ^2 \left| y\right| ^{2-N} d{y} \right) \left( \int _{B_r} \left| \nabla v_2\left( y \right) \right| ^2 \left| y\right| ^{2-N} d{y} \right) \end{aligned}$$

is monotonically non-decreasing in *r*.

Different versions of this formula were proved in [9] by Caffarelli, and by Caffarelli and Kenig in [10] to show the regularity of solutions to parabolic equations. Caffarelli, Jerison, and Kenig in [11] further extended these ideas, proving a powerful uniform bound on the monotonicity functional, instead of a monotonicity result. Later on, Matevosyan and Petrosyan [45] proved another such uniform bound for non-homogeneous elliptic and parabolic operators with variable-coefficients. ACF-type monotonicity formulas have also been used to study almost minimizers of variable-coefficient Bernoulli-type functionals, see for example [19].

In this section, we prove a parabolic version of theorem 6.1, given below in Theorem 6.4. To the best of our knowledge, this result is also new.

As in the previous section, to employ the high-dimensional limiting technique to prove this parabolic result, we first need a version of it for solutions to non-homogeneous variable-coefficient elliptic equations. As similar results for homogeneous variable-coefficient elliptic equations have found numerous applications, this monotonicity result could be interesting in its own right. Once we have the suitable elliptic result in hand, we employ techniques similar to those in the previous section to establish our parabolic ACF result.

### Corollary 6.2

For some $R > 0$, let $B_R \subset \mathbb {R}^N$. For each $i = 1,2$, we make the following assumptions: Let $\kappa _i: B_R \rightarrow \mathbb {R}_+$ be bounded, elliptic, and regular in the sense that $0< \lambda \le \kappa _i \le \Lambda < \infty$ in $B_R$ and $\nabla \log \kappa _i \cdot y\in L^\infty (B_R)$. Define $\Upsilon _i \ge \left\| \nabla \log \kappa _i \cdot y\right\| _{L^\infty (B_R)}$. Let $v_i \in C^0\left( B_R \right) \cap W^{1,2}\left( B_R, \kappa _i \, dy \right)$ be a non-negative function for which $v_i\left( 0 \right) = 0$, and $\displaystyle {{\,\textrm{div}\,}}(\kappa _i \nabla v_i) \ge \kappa _i \, \ell _i$ in $B_R$ in the sense of distributions, where $\ell _i^-$ is integrable with respect to $\kappa _i \, v_i \left| y\right| ^{2 + \Upsilon _i - N} dy$ on each $B_r \subset B_R$. Assume further that for every $r < R$, $\Gamma _{i, r}:= \text {supp}v_i \cap S_r:= \text {supp}v_i \cap \partial B_r$ has non-zero measure. Finally, assume that $N \ge \max \left\{ \Upsilon _1, \Upsilon _2\right\} + 2$ and that $v_1 v_2 \equiv 0$. Then for all $r < R$, we define $\phi \left( r \right) = \phi \left( r; v_1, v_2, \kappa _1, \kappa _2 \right)$ as

$$\begin{aligned} \phi (r)=\frac{1}{r^{4+ \Upsilon _1 + \Upsilon _2}}\left( \int _{B_r} \kappa _1(y) |\nabla v_1(y)|^2 |y|^{2+\Upsilon _1-N} dy \right) \left( \int _{B_r} \kappa _2(y) |\nabla v_2(y)|^2 |y|^{2+\Upsilon _2-N} dy \right) . \end{aligned}$$

If we set ${\widetilde{\phi }}\left( r \right) = r^{\mu } \phi \left( r \right)$, where $\mu = 4\left( \frac{\Lambda - \lambda }{\Lambda } \right) + \Upsilon _1 + \Upsilon _2$, then it holds that

38 $$\begin{aligned} \begin{aligned} \frac{d}{dr} {\widetilde{\phi }}\left( r \right)&\ge - \frac{2r^{4 \left( \frac{\Lambda - \lambda }{\Lambda } \right) -3}}{ \left( N-2 \right) } \frac{\left( \int _{B_r} \kappa _{1} \, \ell _{1}^- v_{1} \left| y\right| ^{2+ \Upsilon _1-N} dy \right) \left( \int _{B_r} \kappa _{2} \left| \nabla v_{2}\right| ^2 \left| y\right| ^{2+ \Upsilon _2-N} d{y} \right) \left( \int _{S_r} \kappa _{1} \left| \nabla v_{1}\right| ^2 d\sigma \right) }{\left( \int _{S_r}\kappa _{1} \left| v_{1}\right| ^2 d\sigma \right) } \\&\quad - \frac{2r^{4 \left( \frac{\Lambda - \lambda }{\Lambda } \right) -3}}{\left( N-2 \right) } \frac{ \left( \int _{B_r} \kappa _{1} \left| \nabla v_{1}\right| ^2 \left| y\right| ^{2+ \Upsilon _1-N} d{y} \right) \left( \int _{B_r} \kappa _{2} \, \ell _{2}^- v_{2} \left| y\right| ^{2+ \Upsilon _2-N} dy \right) \left( \int _{S_r} \kappa _{2} \left| \nabla v_{2}\right| ^2 d\sigma \right) }{\left( \int _{S_r}\kappa _{2} \left| v_{2}\right| ^2 d\sigma \right) }. \end{aligned} \end{aligned}$$

### Remark 6.3

Notice that the main difference between our $\phi (r)$ and the one defined in (37) is the introduction of $\Upsilon _1$ and $\Upsilon _2$, which depend on $\nabla \kappa _1$ and $\nabla \kappa _2$, respectively, appearing in the powers on $\left| y\right|$. In fact, if we set $\kappa _i = 1$, we recover a monotonicity formula very similar to [18, Corollary 2], which generalizes Proposition 6.1 to the non-homogeneous setting. If each $\ell _i = 0$, the right-hand side of (38) vanishes and we reach a true monotonicity result.

### Proof

Observe that for a.e. *r*,

$$\begin{aligned} \frac{d}{dr} \int _{B_r} \kappa _i \left| \nabla v_i\right| ^2 \left| y\right| ^{2 + \Upsilon _i - N} d{y} = r^{2 + \Upsilon _i - N} \int _{S_r} \kappa _i \left| \nabla v_i\right| ^2 d\sigma (y). \end{aligned}$$

Therefore, for a.e. *r*,

39 $$\begin{aligned} \begin{aligned} \phi ^\prime \left( r \right) =&- \frac{4+ \Upsilon _1 + \Upsilon _2}{r^{5+ \Upsilon _1 + \Upsilon _2}} \left( \int _{B_r} \kappa _1 \left| \nabla v_1\right| ^2 \left| y\right| ^{2+ \Upsilon _1-N} d{y} \right) \left( \int _{B_r} \kappa _2 \left| \nabla v_2\right| ^2 \left| y\right| ^{2+ \Upsilon _2-N} d{y} \right) \\&+ \frac{r^{2-N}}{r^{4 + \Upsilon _2}} \left( \int _{S_r} \kappa _1 \left| \nabla v_1\right| ^2 d\sigma (y) \right) \left( \int _{B_r} \kappa _2 \left| \nabla v_2\right| ^2 \left| y\right| ^{2+ \Upsilon _2-N} d{y} \right) \\&+ \frac{r^{2-N}}{r^{4+ \Upsilon _1}} \left( \int _{B_r} \kappa _1 \left| \nabla v_1\right| ^2 \left| y\right| ^{2+ \Upsilon _1-N} d{y} \right) \left( \int _{S_r} \kappa _2 \left| \nabla v_2\right| ^2 d\sigma (y) \right) . \end{aligned} \end{aligned}$$

We want to estimate this derivative.

Throughout this part of the proof, we suppress subscripts for $i = 1,2$ on all functions and exponents. That is, in place of $v_i$, $\kappa _i$, $\ell _i$, $\Upsilon _i$, we write *v*, $\kappa$, $\ell$, and $\Upsilon$. Since $\displaystyle {{\,\textrm{div}\,}}(\kappa \nabla v)\ge \kappa \, \ell$ in $B_R$, then $\Delta v\ge l$, where we define $l:= -\left( \ell ^- + \nabla \log \kappa \cdot \nabla v \right)$. Define $v_{m}$ and $l_{m}$ to be the mollifications of *v* and *l*, respectively, at scale $m^{-1}$. Let $A_{r, \varepsilon } = B_r {\setminus } B_\varepsilon$ for some $\varepsilon \in \left( 0, r \right)$. Integration by parts shows that

$$\begin{aligned}&r^{1 + \Upsilon - N} \int _{S_r} \kappa \left| v_m\right| ^2 d\sigma \\&\quad = \int _{S_r} \kappa \left| v_m\right| ^2 \left| y\right| ^{\Upsilon -N} y \cdot {\hat{n}} \, d\sigma (y) \\&\quad = \int _{A_{r, \varepsilon }} {{\,\textrm{div}\,}}\left( \kappa \left| v_m\right| ^2 \left| y\right| ^{\Upsilon -N} y \right) dy + \int _{S_\varepsilon } \kappa \left| v_m\right| ^2 \left| y\right| ^{\Upsilon -N} y \cdot {\hat{n}} \, d\sigma (y)\\&\quad = \int _{A_{r, \varepsilon }} \left( \Upsilon \kappa + \nabla \kappa \cdot y \right) \left| v_m\right| ^2 \left| y\right| ^{\Upsilon -N} dy + 2 \int _{A_{r, \varepsilon }} \kappa \, v_m \nabla v_m \cdot y \left| y\right| ^{\Upsilon -N} dy \\&\qquad + \varepsilon ^{1+ \Upsilon -N } \int _{S_\varepsilon } \kappa \left| v_m\right| ^2 \, d\sigma (y). \end{aligned}$$

Moreover,

$$\begin{aligned}&r^{1 + \Upsilon - N } \int _{S_r} \kappa \, v_m\nabla v_m\cdot y \, d\sigma (y) = \int _{S_r} \kappa \, v_m \left| y\right| ^{2+ \Upsilon -N} \nabla v_m\cdot {\hat{n}} \, d\sigma (y) \\&\quad = \int _{A_{r, \varepsilon }} {{\,\textrm{div}\,}}\left( \kappa \nabla v_m \, v_m \left| y\right| ^{2+ \Upsilon -N} \right) dy + \int _{S_\varepsilon } \kappa \, v_m \left| y\right| ^{2+ \Upsilon -N} \nabla v_m\cdot {\hat{n}} \, d\sigma (y) \\&\quad = \int _{A_{r, \varepsilon }} \kappa \left| \nabla v_m\right| ^2 \left| y\right| ^{2+ \Upsilon -N} dy + \int _{A_{r, \varepsilon }} \kappa \left[ \Delta v_m + \frac{\nabla \kappa }{\kappa } \cdot \nabla v_m\right] v_m \left| y\right| ^{2+ \Upsilon -N} dy \\&\qquad + \left( 2 + \Upsilon - N \right) \int _{A_{r, \varepsilon }} \kappa \, v_m \nabla v_m \cdot y \left| y\right| ^{\Upsilon -N} dy + \varepsilon ^{2+ \Upsilon -N} \int _{S_\varepsilon } \kappa \, v_m\nabla v_m\cdot {\hat{n}} \, d\sigma (y). \end{aligned}$$

Since $\displaystyle \Delta v_{m} \ge l_{m} = - \left( \ell ^- \right) _{m} - \left( \nabla \log \kappa \cdot \nabla v \right) _{m}$ and $\Upsilon + \nabla \log \kappa \cdot y \ge 0$ in $B_R$, then

$$\begin{aligned}&\frac{N - \Upsilon - 2}{2} r^{1 + \Upsilon - N} \int _{S_r} \kappa \left| v_m\right| ^2 \, d\sigma (y) + r^{1 + \Upsilon - N } \int _{S_r} \kappa \, v_m\nabla v_m\cdot y \, d\sigma (y) \\&\quad \ge \int _{A_{r, \varepsilon }} \kappa \left| \nabla v_m\right| ^2 \left| y\right| ^{2+ \Upsilon -N} dy \\&\quad - \int _{A_{r, \varepsilon }} \kappa \left[ \left( \ell ^- \right) _{m} + \left( \nabla \log \kappa \cdot \nabla v \right) _{m} - \nabla \log \kappa \cdot \nabla v_m\right] v_m \left| y\right| ^{2+ \Upsilon -N} dy \\&\qquad + \frac{N - \Upsilon - 2}{2}\int _{A_{r, \varepsilon }} \left( \Upsilon + \nabla \log \kappa \cdot y \right) \kappa \left| v_m\right| ^2 \left| y\right| ^{\Upsilon -N} dy + I_\varepsilon \\&\ge \int _{A_{r, \varepsilon }} \kappa \left| \nabla v_m\right| ^2 \left| y\right| ^{2+ \Upsilon -N} dy - \int _{A_{r, \varepsilon }} \kappa \, \left( \ell ^- \right) _{m} \, v_m \left| y\right| ^{2+ \Upsilon -N} dy \\&\quad - \int _{A_{r, \varepsilon }} \kappa \left| \left( \nabla \log \kappa \cdot \nabla v \right) _{m} - \nabla \log \kappa \cdot \nabla v_m\right| v_m \left| y\right| ^{2+ \Upsilon -N} dy + I_\varepsilon , \end{aligned}$$

where

$$\begin{aligned} I_{\varepsilon }&= \frac{N - \Upsilon - 2}{2}\varepsilon ^{1+\Upsilon -N} \int _{S_\varepsilon } \kappa \left| v_m\right| ^2 \, d\sigma (y) + \varepsilon ^{2+ \Upsilon -N} \int _{S_\varepsilon } \kappa \, v_m\nabla v_m\cdot {\hat{n}} \, d\sigma (y). \end{aligned}$$

By rearrangement, that $A_{r, \varepsilon } \subset B_r$ and $\Upsilon \ge 0$, it follows that

$$\begin{aligned} \int _{A_{r,\varepsilon }}&\kappa \left| \nabla v_m\right| ^2 \left| y\right| ^{2+ \Upsilon -N} dy \le \frac{N - 2}{2} r^{1 + \Upsilon - N} \int _{S_r} \kappa \left| v_m\right| ^2 \, d\sigma (y) \\&\quad + r^{1 + \Upsilon - N } \int _{S_r} \kappa \, v_m\nabla v_m\cdot y \, d\sigma (y) \\&\quad + \int _{B_{r}} \kappa \, \left( \ell ^- \right) _{m} v_m \left| y\right| ^{2+ \Upsilon -N} dy \\&\quad + \int _{B_{r}} \kappa \left| \left( \nabla \log \kappa \cdot \nabla v \right) _{m} - \nabla \log \kappa \cdot \nabla v_m\right| v_m \left| y\right| ^{2+ \Upsilon -N} dy - I_\varepsilon . \end{aligned}$$

Since $\nabla v_m$ is bounded and $\Upsilon \ge 0$, then $I_{\varepsilon } \rightarrow 0$ as $\varepsilon \rightarrow 0$. In particular, the right-hand side of the previous inequality is bounded independent of $\varepsilon$. Accordingly, we may take $\varepsilon \rightarrow 0$, eliminate $I_\varepsilon$ on the right-hand side, and replace $A_{r, \varepsilon }$ with $B_r$ on the left.

Now we integrate with respect to *r*, $r_0< r < r_0 + \delta$, divide through by $\delta$, then take $m \rightarrow \infty$ to getBy taking $\delta \rightarrow 0$, it follows that for a.e. $r_0 > 0$

$$\begin{aligned}&\int _{B_{r_0}} \kappa \left| \nabla v\right| ^2 \left| y\right| ^{2+ \Upsilon -N} dy \le \frac{N - 2}{2} r_0^{1 + \Upsilon - N} \int _{S_{r_0}} \kappa \, \left| v\right| ^2 \, d\sigma (y)\\&\quad + r_0^{1 + \Upsilon - N }\int _{S_{r_0}} \kappa \, v\nabla v\cdot y \, d\sigma (y) + \int _{B_{r_0}} \kappa \, \ell ^- v \left| y\right| ^{2+ \Upsilon -N} dy. \end{aligned}$$

After reintroducing the subscripts, we see that for a.e. $r > 0$,

40 $$\begin{aligned} \int _{B_{r}} \kappa _i\left| \nabla v_{i}\right| ^2 \left| y\right| ^{2+ \Upsilon _i-N} dy&{-} \int _{B_{r}} \kappa _i \, \ell _{i}^- v_{i} \left| y\right| ^{2+ \Upsilon _i-N} dy \le \frac{N - 2}{2} r^{1 + \Upsilon _i - N} \int _{S_{r}} \kappa _i \left| v_{i}\right| ^2 \, d\sigma (y) \nonumber \\&+ r^{1 + \Upsilon _i - N } \int _{S_{r}} \kappa _i \, v_{i}\nabla v_{i}\cdot y \, d\sigma (y). \end{aligned}$$

By rescaling, we may assume that $r = 1$.

Let $$\nabla _\theta w$$ denote the gradient of function *w* on $$S^{N-1}$$, the unit sphere. Let $$\Gamma _i$$ denote the support of $$v_i$$ on $$S^{N-1}$$ for $$i = 1, 2$$. By assumption, the measures of $${\Gamma _1}$$ and $${\Gamma _2}$$ are non-zero. For $$i = 1,2 $$, define

$$\begin{aligned} \frac{1}{\alpha _i} = \inf \left\{ \frac{\int _{\Gamma _i} \left| \nabla _\theta w\right| ^2 }{\int _{\Gamma _i} w^2} : w \in H^{1,2}_0\left( \Gamma _i \right) \right\} . \end{aligned}$$

Observe that for any $\beta _i \in \left( 0,1 \right)$,

$$\begin{aligned} \frac{1 - \beta _i^2}{\alpha _i} \int _{S_1} \left| v_i\right| ^2 d\sigma (y)&\le \left( 1 - \beta _i^2 \right) \int _{S_1} \left| \nabla _\theta v_i\right| ^2 d\sigma (y) \end{aligned}$$

and

$$\begin{aligned} \frac{2 \beta _i}{\sqrt{\alpha _i}} \int _{S_1} \left| v_i\right| \left| \nabla v_i \cdot y\right| d\sigma (y)&\le 2 \left( \frac{\beta _i^2}{ \alpha _i} \int _{S_1} \left| v_i\right| ^2 d\sigma (y) \right) ^{\frac{1}{2}} \left( \int _{S_1} \left( \nabla v_i \cdot y \right) ^2 d\sigma (y) \right) ^{\frac{1}{2}} \\&\le \frac{\beta _i^2}{\alpha _i} \int _{S_1} \left| v_i\right| ^2 d\sigma (y) + \int _{S_1} \left| \nabla _r v_i\right| ^2 d\sigma (y) \\&\le \int _{S_1} \left[ \beta _i^2\left| \nabla _\theta v_i\right| ^2 + \left| \nabla _r v_i\right| ^2\right] d\sigma (y) . \end{aligned}$$

If we set

41 $$\begin{aligned} \frac{1 - \beta _i^2}{\alpha _i} = \frac{\beta _i \left( N-2 \right) }{\sqrt{\alpha _i}} \end{aligned}$$

for $i = 1,2$, then by combining (40) with the last two inequalities and using that $\lambda \le \kappa _i(y) \le \Lambda$, we have

$$\begin{aligned}&\int _{B_1} \kappa _i \left| \nabla v_i\right| ^2 \left| y\right| ^{2+\Upsilon _i-N} d{y} - \int _{B_1} \kappa _i \, \ell ^-_{i} v_{i} \left| y\right| ^{2+ \Upsilon _i-N} dy \le \frac{N - 2}{2} \int _{S_{1}} \kappa _i \left| v_{i}\right| ^2 \, d\sigma (y) \\&\qquad + \int _{S_1} \kappa _i \, v_{i}\nabla v_{i}\cdot y \, d\sigma (y) \\&\quad \le \frac{\Lambda \left( N - 2 \right) }{2} \int _{S_{1}} \left| v_{i}\right| ^2\, d\sigma (y) + \Lambda \int _{S_1} \left| v_{i}\right| \left| \nabla v_{i}\cdot y\right| \, d\sigma (y) \\&\quad \le \frac{\Lambda \sqrt{\alpha }_i}{2\beta _i} \int _{S_{1}} \left| \nabla v_{i}\right| ^2\, d\sigma (y) \\&\quad \le \frac{\Lambda \sqrt{\alpha }_i}{2 \lambda \beta _i} \int _{S_{1}} \kappa _i \left| \nabla v_{i}\right| ^2\, d\sigma (y). \end{aligned}$$

Substituting these inequalities into (39) with $r = 1$ gives

$$\begin{aligned} \phi ^\prime \left( 1 \right)&\ge - \left( 4+ \Upsilon _1 + \Upsilon _2 \right) \left( \int _{B_1} \kappa _1 \left| \nabla v_1\right| ^2 \left| y\right| ^{2+ \Upsilon _1-N} d{y} \right) \left( \int _{B_1} \kappa _2 \left| \nabla v_2\right| ^2 \left| y\right| ^{2+ \Upsilon _2-N} d{y} \right) \\&\quad + \frac{2 \lambda \beta _1}{\Lambda \sqrt{\alpha }_1} \left( \int _{B_1} \kappa _1 \left| \nabla v_1\right| ^2 \left| y\right| ^{2+\Upsilon _1-N} d{y} - \int _{B_1} \kappa _1 \, \ell ^-_{1} v_{1} \left| y\right| ^{2+ \Upsilon _1-N} dy \right) \left( \int _{B_1} \kappa _2 \left| \nabla v_2\right| ^2 \left| y\right| ^{2+ \Upsilon _2-N} d{y} \right) \\&\quad + \frac{2 \lambda \beta _2}{\Lambda \sqrt{\alpha }_2} \left( \int _{B_1} \kappa _1 \left| \nabla v_1\right| ^2 \left| y\right| ^{2+ \Upsilon _1-N} d{y} \right) \left( \int _{B_1} \kappa _2 \left| \nabla v_2\right| ^2 \left| y\right| ^{2+\Upsilon _2-N} d{y} - \int _{B_1} \kappa _2 \, \ell ^-_{2} v_{2} \left| y\right| ^{2+ \Upsilon _2-N} dy \right) \\&= \left( \frac{2 \lambda }{\Lambda }\left[ \frac{\beta _1}{\sqrt{\alpha }_1}+ \frac{\beta _2}{\sqrt{\alpha }_2}\right] - 4- \Upsilon _1 - \Upsilon _2 \right) \left( \int _{B_1} \kappa _1 \left| \nabla v_1\right| ^2 \left| y\right| ^{2+ \Upsilon _1-N} d{y} \right) \left( \int _{B_1} \kappa _2 \left| \nabla v_2\right| ^2 \left| y\right| ^{2+ \Upsilon _2-N} d{y} \right) \\&\quad - \frac{2 \lambda \beta _1}{\Lambda \sqrt{\alpha }_1} \left( \int _{B_1} \kappa _1 \, \ell _{1}^- v_{1} \left| y\right| ^{2+ \Upsilon _1-N} dy \right) \left( \int _{B_1} \kappa _2 \left| \nabla v_2\right| ^2 \left| y\right| ^{2+ \Upsilon _2-N} d{y} \right) \\&\quad - \frac{2 \lambda \beta _2}{\Lambda \sqrt{\alpha }_2} \left( \int _{B_1} \kappa _1 \left| \nabla v_1\right| ^2 \left| y\right| ^{2+ \Upsilon _1-N} d{y} \right) \left( \int _{B_1} \kappa _2 \, \ell _{2}^- v_{2} \left| y\right| ^{2+ \Upsilon _2-N} dy \right) . \end{aligned}$$

The relation (41) is satisfied when

$$\begin{aligned} \frac{\beta _i}{\sqrt{\alpha _i}} = \frac{1}{2} \left\{ \left[ \left( N-2 \right) ^2 + \frac{4}{\alpha _i}\right] ^{\frac{1}{2}} - \left( N-2 \right) \right\} . \end{aligned}$$

If we define $\gamma _i > 0$ so that $\gamma _i \left( \gamma _i + N - 2 \right) = \frac{1}{\alpha _i}$ then $\frac{\beta _i}{\sqrt{\alpha _i}} = \gamma _i$ for $i = 1,2$.

As a function that acts on subsets of $S^{N-1}$, $\gamma$ was studied in [27] and it was shown that $\gamma \left( E \right) \ge \psi \left( \frac{\left| E\right| }{\left| S^{N-1}\right| } \right)$, where $\psi$ is the decreasing, convex function defined by

$$\begin{aligned} \psi \left( s \right) = \left\{ \begin{array}{ll} \frac{1}{2} \log \left( \frac{1}{4s} \right) + \frac{3}{2} &{}\quad {\text {if }} s< \frac{1}{4} \\ 2 \left( 1 - s \right) &{}\quad {\text {if }} \frac{1}{4}< s < 1. \end{array} \right. \end{aligned}$$

We use the notation $\gamma _i = \gamma \left( \Gamma _i \right)$ for $i = 1,2$. With $s_i = \frac{\left| \Gamma _i\right| }{\left| S^{N-1}\right| }$, it follows from convexity that

$$\begin{aligned} \gamma _1 + \gamma _2 \ge \psi \left( s_1 \right) + \psi \left( s_2 \right) \ge 2\psi \left( \frac{s_1 + s_2}{2} \right) \ge 2\psi \left( \frac{1}{2} \right) = 2. \end{aligned}$$

Moreover, since each $v_i \in H^{1,2}_0\left( \Gamma _i \right)$, then

$$\begin{aligned} \begin{aligned} \gamma _i&= \frac{N-2}{2} \left\{ \left[ 1 + \frac{4}{\alpha _i\left( N-2 \right) ^2}\right] ^{\frac{1}{2}} - 1\right\} \le \frac{N-2}{2} \frac{2}{\alpha _i\left( N-2 \right) ^2} \\&= \frac{1}{\alpha _i \left( N -2 \right) } \le \frac{1}{N-2} \frac{\int _{\Gamma _i} \left| \nabla _\theta v_i\right| ^2 }{\int _{\Gamma _i} \left| v_i\right| ^2} \\&\le \frac{1}{N-2} \frac{\int _{S_1} \left| \nabla v_i\right| ^2 }{\int _{S_1} \left| v_i\right| ^2} \le \frac{\Lambda }{\lambda \left( N-2 \right) } \frac{\int _{S_1} \kappa _i \left| \nabla v_i\right| ^2 }{\int _{S_1}\kappa _i \left| v_i\right| ^2 }. \end{aligned} \end{aligned}$$

Therefore,

$$\begin{aligned} \begin{aligned} \phi ^\prime \left( 1 \right)&\ge - \left[ 4 \left( \frac{\Lambda - \lambda }{\Lambda } \right) + \Upsilon _1 + \Upsilon _2\right] \phi \left( 1 \right) \\&\quad - \frac{2}{\left( N-2 \right) }\left( \int _{B_1} \kappa _1 \, \ell _{1}^- v_{1} \left| y\right| ^{2+ \Upsilon _1-N} dy \right) \left( \int _{B_1} \kappa _2 \left| \nabla v_2\right| ^2 \left| y\right| ^{2+ \Upsilon _2-N} d{y} \right) \frac{\int _{S_1} \kappa _1 \left| \nabla v_1\right| ^2 }{\int _{S_1}\kappa _1 \left| v_1\right| ^2 } \\&\quad - \frac{2}{\left( N-2 \right) } \left( \int _{B_1} \kappa _1 \left| \nabla v_1\right| ^2 \left| y\right| ^{2+ \Upsilon _1-N} d{y} \right) \left( \int _{B_1} \kappa _2 \, \ell _{2}^- v_{2} \left| y\right| ^{2+ \Upsilon _2-N} dy \right) \frac{\int _{S_1} \kappa _2 \left| \nabla v_2\right| ^2 }{\int _{S_1}\kappa _2 \left| v_2\right| ^2 }. \end{aligned} \end{aligned}$$

For values of $r \ne 1$, define $v_{i, r}\left( y \right) = r^{-1} v_i\left( r y \right)$ for $i = 1, 2$ and $\kappa _{i,r}(y)=\kappa _i(ry)$, so that for $0<s,r\le 1,$

$$\begin{aligned} \phi \left( s; v_{1, r}, v_{2, r}, \kappa _{1,r}, \kappa _{2,r} \right)&= \frac{1}{s^{4+ \Upsilon _1 + \Upsilon _2}}\left( \int _{B_s} \left| \nabla v_{1,r}\right| ^2 \left| y\right| ^{2 + \Upsilon _1 -N} \kappa _{1,r} \, d{y} \right) \left( \int _{B_s} \left| \nabla v_{2,r}\right| ^2 \left| y\right| ^{2+ \Upsilon _2-N} \kappa _{2,r} \, d{y} \right) \\&= \frac{1}{(sr)^{4+ \Upsilon _1 + \Upsilon _2}} \left( \int _{B_{rs}} \left| \nabla v_1\right| ^2 \left| y\right| ^{2 + \Upsilon _1 -N} \kappa _1 \, d{y} \right) \left( \int _{B_{rs}} \left| \nabla v_2\right| ^2 \left| y\right| ^{2+ \Upsilon _2-N} \kappa _2 \, d{y} \right) \\&= \phi \left( rs; v_1, v_2, \kappa _1, \kappa _2 \right) . \end{aligned}$$

Thus,

$$\begin{aligned} \phi '\left( s; v_{1, r}, v_{2, r}, \kappa _{1,r}, \kappa _{2,r} \right){} & {} = \frac{d}{ds}\phi \left( s; v_{1, r}, v_{2, r}, \kappa _{1,r}, \kappa _{2,r} \right) \\{} & {} = \frac{d}{ds} \phi \left( rs; v_1, v_2, \kappa _1, \kappa _2 \right) = r \phi '\left( rs; v_1, v_2, \kappa _1, \kappa _2 \right) \end{aligned}$$

and taking $s=1$ gives

$$\begin{aligned} \phi '(1; v_{1, r}, v_{2, r}, \kappa _{1,r}, \kappa _{2,r})=r\phi '(r;v_1, v_2, \kappa _1, \kappa _2). \end{aligned}$$

Let $\Gamma _{i, r}$ denote the support of $v_{i}$ on $S_r$ and set $s_{i, r} = \frac{\left| \Gamma _{i, r}\right| }{r^{N-1} \left| S^{N-1}\right| }$ for $i = 1, 2$. With $\ell _{i, r}\left( y \right) = r \ell _i\left( r y \right)$, we have $\displaystyle \text {div}(\kappa _{i,r} \nabla v_{i, r}) \ge \kappa _{i,r} \ell _{i, r}$. Applying the derivative estimates above to the pair $v_{1, r}$, $v_{2, r}$ then rescaling leads to

$$\begin{aligned} \phi ^\prime \left( r \right)&= \frac{1}{r} \phi '(1; v_{1, r}, v_{2, r}, \kappa _{1,r}, \kappa _{2,r}) \\&\ge - \frac{1}{r} \left[ 4 \left( \frac{\Lambda - \lambda }{\Lambda } \right) + \Upsilon _1 + \Upsilon _2\right] \phi (1; v_{1, r}, v_{2, r}, \kappa _{1,r}, \kappa _{2,r}) \\&\quad - \frac{2}{r\left( N-2 \right) }\left( \int _{B_1} \kappa _{1,r} \, \ell _{1,r}^- v_{1,r} \left| y\right| ^{2+ \Upsilon _1-N} dy \right) \\&\quad \times \left( \int _{B_1} \kappa _{2,r} \left| \nabla v_{2,r}\right| ^2 \left| y\right| ^{2+ \Upsilon _2-N} d{y} \right) \frac{\int _{S_1} \kappa _{1,r} \left| \nabla v_{1,r}\right| ^2 }{\int _{S_1}\kappa _{1,r} \left| v_{1,r}\right| ^2 } \\&\quad - \frac{2}{r\left( N-2 \right) } \left( \int _{B_1} \kappa _{1,r} \left| \nabla v_{1,r}\right| ^2 \left| y\right| ^{2+ \Upsilon _1-N} d{y} \right) \left( \int _{B_1} \kappa _{2,r} \, \ell _{2,r}^- v_{2,r} \left| y\right| ^{2+ \Upsilon _2-N} dy \right) \\&\quad \times \frac{\int _{S_1} \kappa _{2,r} \left| \nabla v_{2,r}\right| ^2 }{\int _{S_1}\kappa _{2,r} \left| v_{2,r}\right| ^2 }\\&\quad \ge - \frac{1}{r} \left[ 4 \left( \frac{\Lambda - \lambda }{\Lambda } \right) + \Upsilon _1 + \Upsilon _2\right] \phi \left( r; v \right) \\&\quad - \frac{2}{r^{3 + \Upsilon _1 + \Upsilon _2} \left( N-2 \right) } \left( \int _{B_r} \kappa _{1} \, \ell _{1}^- v_{1} \left| y\right| ^{2+ \Upsilon _1-N} dy \right) \left( \int _{B_r} \kappa _{2} \left| \nabla v_{2}\right| ^2 \left| y\right| ^{2+ \Upsilon _2-N} d{y} \right) \frac{\int _{S_r} \kappa _{1} \left| \nabla v_{1}\right| ^2 }{\int _{S_r}\kappa _{1} \left| v_{1}\right| ^2 } \\&\quad - \frac{2}{r^{3 + \Upsilon _1 + \Upsilon _2}\left( N-2 \right) } \left( \int _{B_r} \kappa _{1} \left| \nabla v_{1}\right| ^2 \left| y\right| ^{2+ \Upsilon _1-N} d{y} \right) \left( \int _{B_r} \kappa _{2} \, \ell _{2}^- v_{2} \left| y\right| ^{2+ \Upsilon _2-N} dy \right) \frac{\int _{S_r} \kappa _{2} \left| \nabla v_{2}\right| ^2 }{\int _{S_r}\kappa _{2} \left| v_{2}\right| ^2 }. \end{aligned}$$

That is, with $\mu = 4 \left( \frac{\Lambda - \lambda }{\Lambda } \right) + \Upsilon _1 + \Upsilon _2$, the conclusion described by (38) follows. $\square$

In what follows, we introduce our new parabolic ACF-type result for variable-coefficient operators. We prove this result using only Corollary 6.2 and the tools and ideas that have been developed so far in this paper. As in the previous section, we first discuss the kinds of solutions that we work with.

Let $u: \mathbb {R}^d \times \left( 0, T \right) \rightarrow \mathbb {R}$ have moderate *h*-growth at infinity, as described in Definition 3.8. For every $t \in \left( 0, T \right)$, assume first that *u* is sufficiently regular to define the functionals $\mathcal {D}\left( t; u, h \right)$ and $\mathcal {T}\left( t; u, h \right)$ from (16) as well as

$$\begin{aligned} \begin{aligned} \mathcal {J}^-(t)&= \mathcal {J}^-(t; u,h) = \int _{\mathbb {R}^d} J^-(x,t) \left| u\left( x,t \right) \right| \exp \left( -\frac{\left| h(x)\right| ^2}{4t} \right) dx, \end{aligned} \end{aligned}$$

where $J(x,t) = J(x, t; u,h)$ is as defined in (25) and all derivatives are interpreted in the weak sense. Then we say that such a function *u* belongs to the function class $\mathfrak {C}\left( \mathbb {R}^d \times \left( 0, T \right) , h \right)$ if *u* has moderate *h*-growth at infinity (so is consequently continuous), and for every $t_0 \in \left( 0, T \right)$, there exists $\varepsilon \in \left( 0, t_0 \right)$ so that

42 $$\begin{aligned} \mathcal {D} \in L^\infty \left[ t_0 - \varepsilon , t_0\right] , \quad \mathcal {T} \in L^\infty \left[ t_0 - \varepsilon , t_0\right] \end{aligned}$$

and

43 $$\begin{aligned} \mathcal {J}^- \in L^{1}\left( \left[ 0, t_0\right] , t^{- \frac{d}{2}} dt \right) . \end{aligned}$$

This is the class of functions that we consider in our result. As before, this may not be the weakest setting in which our proof holds.

### Theorem 6.4

For each $i = 1, 2$, we make the following assumptions: Let $\text {tr}\left( H_i \left\langle \nabla _z G_i(h_i),h_i \right\rangle \right) \in L^\infty (\mathbb {R}^d)$, where $h_i$, $H_i$, and $G_i$ are described by (4), (2), and (5). Define $\Upsilon _i:= \left\| \text {tr}(H_i \left\langle \nabla G_i(h_i), h_i \right\rangle \right\| _{L^\infty \left( \mathbb {R}^d \right) }$ and set $A_i = H_i^{-1}\left( H_i^{-1} \right) ^T: \mathbb {R}^d \rightarrow \mathbb {R}^{d \times d}$. Let $u_i \in \mathfrak {C}\left( \mathbb {R}^d \times \left( 0, T \right) , h_i \right)$ be a non-negative function with $u_i\left( 0,0 \right) = 0$ and $\displaystyle {{\,\textrm{div}\,}}\left( A_i \nabla u_i \right) + \partial _t u_i \ge 0$ in $\mathbb {R}^d \times \left( 0,T \right)$ in the sense of distributions. Assume also that $u_1 u_2 \equiv 0$. For every $t \in \left( 0, T \right)$, define $\Phi \left( t \right) = \Phi \left( t; u_1, u_2, h_1, h_2 \right)$ as

$$\begin{aligned} \Phi \left( t \right){} & {} = \frac{1}{t^{2}} \left( \int _0^t \int _{\mathbb {R}^d} \left( \frac{s}{t} \right) ^{\frac{\Upsilon _1}{2}} \langle A_1 \nabla u_1,\nabla u_1\rangle e^{-\frac{\left| h_1(x)\right| ^2}{4s}} dx \, ds \right) \\{} & {} \quad \times \left( \int _0^t \int _{\mathbb {R}^d} \left( \frac{s}{t} \right) ^{\frac{\Upsilon _2}{2}} \langle A_2 \nabla u_2,\nabla u_2\rangle e^{-\frac{\left| h_2(x)\right| ^2}{4s}} dx \, ds \right) . \end{aligned}$$

Set ${\widetilde{\Phi }}\left( t \right) = t^{\frac{\mu }{2}} \Phi \left( t \right)$, where $\mu = 4\left( \frac{\Lambda - \lambda }{\Lambda } \right) + \Upsilon _1 + \Upsilon _2$ with $\lambda ^{-1} = \max _{i=1,2}\left\{ \left\| \det H_i\right\| _{L^\infty }\right\}$ and $\Lambda = \max _{i = 1,2}\left\{ \left\| \det H_i^{-1}\right\| _{L^\infty }\right\}$. Then ${\widetilde{\Phi }}\left( t \right)$ is monotonically non-decreasing in *t*.

### Proof

Recall that $B_T^n \subset \mathbb {R}^{d \times n}$ is given by (15). For each $i = 1,2$, let $\kappa _{i,n}$ be as defined in Lemma 2.1. By definition, $\lambda \le \kappa _{i,n} \le \Lambda$ for every $n \in \mathbb {N}$. As

$$\begin{aligned} \left\| \nabla _y \log \kappa _{i, n} \cdot y\right\| _{L^\infty \left( B_{\sqrt{2dT}} \right) } \le \left\| \text {tr}(H_i \left\langle \nabla G_i(h_i), h_i \right\rangle )\right\| _{L^\infty \left( \mathbb {R}^d \right) } = \Upsilon _i, \end{aligned}$$

then $\nabla _y \log \kappa _{i, n} \cdot y \in L^\infty \left( B_n^T \right)$. Define each $\Upsilon _{i,n}:= \Upsilon _i$ to be independent of *n*.

Let $u_1$, $u_2$ be as in the statement of the theorem. For each $i = 1,2$ and $n \in \mathbb {N}_{\ge N}$, define $v_{i, n}: B_T^n \subset \mathbb {R}^{d \times n} \rightarrow \mathbb {R}$ so that

$$\begin{aligned} v_{i,n}\left( y \right) = u_i\left( F_{d,n}\left( y \right) \right) . \end{aligned}$$

Since each $u_i$ has moderate $h_i$-growth at infinity, then by Lemma 3.9, $v_{i,n} \in C^0\left( B_T^n \right) \cap W^{1,2}\left( B_T^n, \kappa _{i,n} \, dy \right)$. Moreover, each $v_{i,n}$ is non-negative and $v_{1,n} v_{2,n} \equiv 0$. Assuming without loss of generality that $g(0)=0$, we obtain $v_{i,n}\left( 0 \right) = u_i\left( 0,0 \right) = 0$.

An application of Lemma 2.2 shows that

$$\begin{aligned} \frac{\text {div} ( \kappa _{i,n}\nabla v_{i,n})}{\kappa _{i,n}}&= n \left\{ \text {div} (A_i \nabla u_i) + \frac{\partial u_i}{\partial t} + \frac{1}{dn}\left[ 2 \left\langle A_i \nabla \partial _t u_i , H_i^T h_i \right\rangle \right. \right. \\&\quad \left. \left. + \frac{\partial u_i}{\partial t} \text {tr}(H_i \left\langle \nabla _z G_i(h_i), h_i \right\rangle ) + 2t \frac{\partial ^2 u_i}{\partial t^2} \right] \right\} \\&\ge \frac{1}{d} \left[ 2 \left\langle A_i \nabla \partial _t u_i , H_i^T h_i \right\rangle + \frac{\partial u_i}{\partial t} \text {tr}(H_i \left\langle \nabla _z G_i(h_i), h_i \right\rangle ) + 2t \frac{\partial ^2 u_i}{\partial t^2} \right] =: J_i(x,t), \end{aligned}$$

where $J_i = J(u_i)$ is defined in (25) and does not depend on *n*. For every *n*, define $\ell _{i,n}: B_{T}^n \rightarrow \mathbb {R}$ so that

$$\begin{aligned} \ell _{i,n}\left( y \right) = J_i\left( F_{d,n}\left( y \right) \right) \end{aligned}$$

and then

$$\begin{aligned} {{\,\textrm{div}\,}}\left( \kappa _{i,n} \nabla v_{i,n} \right) \ge \kappa _{i,n} \ell _{i,n}. \end{aligned}$$

Since each $u_i \in \mathfrak {C}\left( \mathbb {R}^{d} \times \left( 0,T \right) , h_i \right)$, then (43) in combination with Corollary 3.7 shows that $\ell _{i, n}^- v_{i, n}$ is integrable with respect to $\kappa _n \, dy$ in each $B_t^n$. In other words, each $\ell _{i,n}^-$ is integrable with respect to $\kappa _{i,n} v_{i,n} \left| y\right| ^{2 + \Upsilon _i - dn} dy$ on each $B_t^n$.

Let $\Gamma _{i, n, t} = \text {supp} \ v_{i, n} \cap S_t^n$. For each *t*, the measure of $\text {supp}\ u_i\left( \cdot , t \right)$ vanishes if and only if the measure of $\Gamma _{i, n, t}$ vanishes for every *n*. We assume first that for every *t*, the measures of $\text {supp} \ u_1\left( \cdot , t \right)$ and $\text {supp} \ u_2\left( \cdot , t \right)$ are non-vanishing. Therefore, for every *i*, *n* and *t*, $\Gamma _{i, n, t}$ has non-zero measure. Thus, we may apply Corollary 6.2 to each pair $v_{1, n}$, $v_{2, n}$ on any ball $B_t^n$ for $t \in \left( 0, T \right)$.

Define $\Phi _n\left( t \right) = \frac{4}{\left( n \left| S^{d n - 1}\right| \right) ^2} \phi \left( \sqrt{2 d t}; v_{1, n}, v_{2, n}, \kappa _{1, n}, \kappa _{2, n} \right)$, where $\phi (r)$ is given in Corollary 6.2. By Lemmas 2.2 and 3.5

44 $$\begin{aligned} \begin{aligned}&\int _{B_t^n} \kappa _{i, n}(y) \left| \nabla v_{i,n}(y) \right| ^2 \left| y\right| ^{2 + \Upsilon _i -d n} dy\\&\quad = dn \left| S^{d n - 1 }\right| \int _0^t \int _{\mathbb {R}^d} \left( 2 d s \right) ^{\frac{\Upsilon _i}{2}} \left\langle A_i \nabla u_i, \nabla u_i \right\rangle K_n(h_i(x),s)dx ds \\&\quad \quad + 2 \left| S^{d n - 1 }\right| \int _0^t \int _{\mathbb {R}^d} \left( 2 d s \right) ^{\frac{\Upsilon _i}{2}} \frac{ \partial u_i}{ \partial s} \left[ \left\langle A_i \nabla u_{i}, H_i^T h_i \right\rangle + s \frac{ \partial u_i}{ \partial s} \right] K_n(h_i(x),s)dx ds. \end{aligned} \end{aligned}$$

Therefore,

$$\begin{aligned} \Phi _n\left( t \right)&= \frac{4 }{\left( n \left| S^{d n - 1}\right| \right) ^2} \frac{1}{\left( 2 d t \right) ^{2 + \frac{\Upsilon _1 + \Upsilon _2}{2}}} \left( \int _{B_t^n} \kappa _{1,n}(y) \left| \nabla v_{1, n}\left( y \right) \right| ^2 \left| y\right| ^{2 + \Upsilon _1 - d n} dy \right) \left( \int _{B_t^n} \kappa _{2,n}(y)\left| \nabla v_{2, n}\left( y \right) \right| ^2 \left| y\right| ^{2 + \Upsilon _2 - d n} dy \right) \\&= \frac{1}{t^{2}} \int _0^t \int _{\mathbb {R}^d} \left( \frac{s}{t} \right) ^{\frac{\Upsilon _1}{2}} \left[ \left\langle A_1\, \nabla u_1, \nabla u_1 \right\rangle + \frac{2}{nd} \frac{ \partial u_1}{ \partial s} \left( \left\langle A_1 \, \nabla u_{1}, H_1^T h_1 \right\rangle + s \frac{ \partial u_1}{ \partial s} \right) \right] K_{n}(h_1(x), s)\, dx ds \\&\quad \times \int _0^t \int _{\mathbb {R}^d} \left( \frac{s}{t} \right) ^{\frac{\Upsilon _2}{2}} \left[ \left\langle A_2\, \nabla u_2, \nabla u_2 \right\rangle + \frac{2}{nd} \frac{ \partial u_2}{ \partial s} \left( \left\langle A_2\, \nabla u_{2}, H_2^T h_2 \right\rangle + s \frac{ \partial u_2}{ \partial s} \right) \right] K_{n}(h_2(x), s) \, dx ds. \end{aligned}$$

It follows from (13), (14) and the Dominated Convergence Theorem that

$$\begin{aligned} \lim _{n \rightarrow \infty } \Phi _n\left( t \right)&= \frac{1}{t^{2}} \prod _{i = 1,2} \left( \int _0^t \int _{\mathbb {R}^d} \left( \frac{s}{t} \right) ^{\frac{\Upsilon _i}{2}} \left\langle A_i\, \nabla u_i, \nabla u_i \right\rangle K(h_i(x), s)\, dx ds \right) = \Phi \left( t \right) . \end{aligned}$$

Set $\mu = 4\left( \frac{\Lambda - \lambda }{\Lambda } \right) + \Upsilon _1 + \Upsilon _2$. If we then define

$$\begin{aligned} {\widetilde{\Phi }}_n(t) = \frac{4 \left( 2d \right) ^{- \frac{\mu }{2}}}{\left( n \left| S^{d n - 1}\right| \right) ^2} {\widetilde{\phi }}\left( \sqrt{2 d t}\right) = \frac{4 \left( 2d \right) ^{- \frac{\mu }{2}}}{\left( n \left| S^{d n - 1}\right| \right) ^2} \left( 2 d t \right) ^{\frac{\mu }{2}}\phi \left( \sqrt{2 d t}\right) , \end{aligned}$$

then by analogous arguments, we see that

45 $$\begin{aligned} \lim _{n \rightarrow \infty } {\widetilde{\Phi }}_n\left( t \right) = {\widetilde{\Phi }}\left( t \right) := t^{\frac{\mu }{2}} \Phi \left( t \right) . \end{aligned}$$

Moreover, an application of the chain rule shows that

$$\begin{aligned} \frac{d}{dt} {\widetilde{\Phi }}_n(t)&= \frac{4 \left( 2d \right) ^{- \frac{\mu }{2}}}{\left( n \left| S^{d n - 1}\right| \right) ^2} \widetilde{\phi }^\prime \left( \sqrt{2 d t}\right) \sqrt{\frac{d}{2t}} = \frac{2 \left( 2d \right) ^{\frac{1- \mu }{2}}}{\left( n \left| S^{d n - 1}\right| \right) ^2} \frac{1}{\sqrt{t}} {\widetilde{\phi }}^\prime \left( \sqrt{2 d t}\right) . \end{aligned}$$

By Corollary 6.2,Using the functionals that were introduced in 33 and 16, applications of Lemma 3.3 show thatandwhere we applied Lemma 2.2, Cauchy–Schwarz, then estimate (14). If we define

$$\begin{aligned} \mathcal {S}_n\left( t; u, \kappa \right) = \mathcal {D}\left( t; u, \kappa \right) + \frac{2t}{dn} \mathcal {T}\left( t; u, \kappa \right) , \end{aligned}$$

then we similarly deduce from (44) that

$$\begin{aligned} \begin{aligned} \int _{B_t^n} \kappa _{i, n}(y) \left| \nabla v_{i,n}(y) \right| ^2&\left| y\right| ^{2 + \Upsilon _i -d n} dy \le \frac{\left( 2 d \right) ^{\frac{\Upsilon _i}{2} + 1} \mathcal {C}_d n \left| S^{d n - 1 }\right| }{\left( 4 \pi \right) ^{\frac{d}{2}}} \int _0^t s^{\frac{\Upsilon _i - d}{2}} \mathcal {S}_n\left( s; u_i, \kappa _i \right) ds. \end{aligned} \end{aligned}$$

Finally, Lemma 3.5 and (14) show that

$$\begin{aligned}&\int _{B_n^t} \kappa _{i,n} \ell _{i,n}^- v_{i,n} \left| y\right| ^{2 + \Upsilon _i -n d} \, d{y}\\&\quad = d \left| S^{d n -1}\right| \int _0^t \int _{\mathbb {R}^d} \left( 2 d s \right) ^{\frac{\Upsilon _i}{2}} J_i^-(x, s) \, u_i\left( x, s \right) \, K_n(h_i(x),s)dx ds \\&\quad \le \frac{d \left( 2 d \right) ^{\frac{\Upsilon _i}{2}}\mathcal {C}_d \left| S^{d n -1}\right| }{\left( 4 \pi \right) ^{\frac{d}{2}}} \int _0^t \int _{\mathbb {R}^d} s^{\frac{\Upsilon _i - d}{2}} J_i^-(x, s) \, u_i\left( x, s \right) \, e^{-\frac{\left| h_i(x)\right| ^2}{4s}} dx ds \\&\quad = \frac{d \left( 2 d \right) ^{\frac{\Upsilon _i}{2}}\mathcal {C}_d \left| S^{d n -1}\right| }{\left( 4 \pi \right) ^{\frac{d}{2}}} \int _0^t s^{\frac{\Upsilon _i - d}{2}} \mathcal {J}^-\left( s; u_i, \kappa _i \right) ds. \end{aligned}$$

Then with $\alpha _{d,n}$ as defined in (51), we get

$$\begin{aligned} {\widetilde{\phi }}^\prime \left( \sqrt{2 d t}\right)&\ge -2 t^{- \frac{3}{2} + 2 \left( \frac{\Lambda - \lambda }{\Lambda } \right) } \frac{\mathcal {C}_d^3 \left( 2 d \right) ^{\frac{1 + \mu }{2}} \left( n \left| S^{d n - 1 }\right| \right) ^2}{\left( d n-2 \right) \left( 4 \pi \right) ^{d} \alpha _{d,n}} \frac{\mathcal {S}_n\left( t; u_1, \kappa _1 \right) }{\mathcal {H}_n\left( t; u_1, \kappa _1 \right) } \\&\quad \times \left( \int _0^t s^{\frac{\Upsilon _1 - d}{2}} \mathcal {J}^-\left( s; u_1, \kappa _1 \right) ds \right) \left( \int _0^t s^{\frac{\Upsilon _2 - d}{2}} \mathcal {S}_n\left( s; u_2, \kappa _2 \right) ds \right) \\&\quad - 2 t^{- \frac{3}{2} + 2 \left( \frac{\Lambda - \lambda }{\Lambda } \right) } \frac{\mathcal {C}_d^3 \left( 2 d \right) ^{\frac{1+\mu }{2}} \left( n \left| S^{d n - 1 }\right| \right) ^2}{\left( d n-2 \right) \left( 4 \pi \right) ^{d} \alpha _{d,n}} \frac{\mathcal {S}_n\left( t; u_2, \kappa _2 \right) }{\mathcal {H}_n\left( t; u_2, \kappa _2 \right) } \\&\quad \times \left( \int _0^t s^{\frac{\Upsilon _2 - d}{2}} \mathcal {J}^-\left( s; u_2, \kappa _2 \right) ds \right) \left( \int _0^t s^{\frac{\Upsilon _1 - d}{2}} \mathcal {S}_n\left( s; u_1, \kappa _1 \right) ds \right) . \end{aligned}$$

Using (52) then shows that

$$\begin{aligned} \frac{d}{dt} {\widetilde{\Phi }}_n(t)&= \frac{2 \left( 2d \right) ^{\frac{1- \mu }{2}}}{\left( n \left| S^{d n - 1}\right| \right) ^2} \frac{1}{\sqrt{t}} {\widetilde{\phi }}^\prime \left( \sqrt{2 d t}\right) \\&\ge - \frac{8 \mathcal {C}_d^3 t^{-\frac{2 \lambda }{\Lambda }}}{n\left( 1-\frac{2}{dn} \right) \left( 4 \pi \right) ^{d}\alpha _{d}} \frac{\mathcal {S}_n\left( t; u_1, \kappa _1 \right) }{\mathcal {H}_n\left( t; u_1, \kappa _1 \right) } \left( \int _0^t s^{\frac{\Upsilon _1 - d}{2}} \mathcal {J}^-\left( s; u_1, \kappa _1 \right) ds \right) \left( \int _0^t s^{\frac{\Upsilon _2 - d}{2}} \mathcal {S}_n\left( s; u_2, \kappa _2 \right) ds \right) \\&\quad - \frac{8 \mathcal {C}_d^3 t^{-\frac{2 \lambda }{\Lambda }}}{n\left( 1-\frac{2}{dn} \right) \left( 4 \pi \right) ^{d} \alpha _{d}} \frac{\mathcal {S}_n\left( t; u_2, \kappa _2 \right) }{\mathcal {H}_n\left( t; u_2, \kappa _2 \right) } \left( \int _0^t s^{\frac{\Upsilon _2 - d}{2}} \mathcal {J}^-\left( s; u_2, \kappa _2 \right) ds \right) \left( \int _0^t s^{\frac{\Upsilon _1 - d}{2}} \mathcal {S}_n\left( s; u_1, \kappa _1 \right) ds \right) \\&=: \widetilde{F}_n(t), \end{aligned}$$

As in the proof of Theorem 5.3, to show that $\widetilde{\Phi }$ is monotone non-decreasing, it suffices to show that given any $t_0 \in (0, T]$, there exists $\delta \in \left( 0, t_0 \right)$ so that $\widetilde{F}_n$ converges uniformly to 0 on $\left[ t_0 - \delta , t_0\right]$.

We first consider the terms in the denominator of ${\widetilde{F}}_n$. For brevity, we set $\mathcal {H}_{n}^{(i)}(t) = \mathcal {H}_{n}\left( t; u_i, \kappa _i \right)$. By repeating the arguments from (34) through (35), we deduce that there exists $N_i \in \mathbb {N}$ and $\delta _i \in \left( 0, t_0 \right)$ so that whenever $n \ge N_i$ and $t \in \left[ t_0 - \delta _i, t_0\right]$, it holds that

$$\begin{aligned} \mathcal {H}^{(i)}_n\left( t \right) \ge H_i e^{- \frac{dN_i \ln 2 }{2} }. \end{aligned}$$

Assumption (43) shows that

$$\begin{aligned} \int _0^t s^{\frac{\Upsilon _i - d}{2}} \mathcal {J}^-\left( s; u_i, \kappa _i \right) ds \le t^{\frac{\Upsilon _i}{2}} \left\| \mathcal {J}^-\left( \cdot ; u_i, \kappa _i \right) \right\| _{L^1\left( \left[ 0, t\right] , s^{- \frac{d}{2}} ds \right) }. \end{aligned}$$

Similarly, since $u_i \in \mathfrak {C}\left( \mathbb {R}^d \times \left( 0, T \right) , h_i \right)$ implies that $u_i$ has moderate $h_i$-growth at infinity, then both $\mathcal {D}\left( \cdot ; u_i, h_i \right)$ and $\mathcal {T}\left( \cdot ; u_i, h_i \right)$ belong to weighted $L^1\left[ 0, t\right]$ and then

$$\begin{aligned}&\int _0^t s^{\frac{\Upsilon _i - d}{2}} \mathcal {S}_n\left( s; u_i, \kappa _i \right) ds \\&\quad \le t^{\frac{\Upsilon _i}{2}}\left( \left\| \mathcal {D}\left( \cdot ; u_i, \kappa _i \right) \right\| _{L^1\left( \left[ 0, t\right] , s^{- \frac{d}{2}} ds \right) } + \frac{2t}{dn} \left\| \mathcal {T}\left( \cdot ; u_i, \kappa _i \right) \right\| _{L^1\left( \left[ 0, t\right] , s^{- \frac{d}{2}} ds \right) } \right) . \end{aligned}$$

Set $\delta = \min \left\{ \delta _1, \delta _2, \frac{t_0}{2}\right\}$, $N = \max \left\{ N_1, N_2\right\}$, and $H = \min \left\{ H_1, H_2\right\}$, then observe that whenever $t \in \left[ t_0 - \delta , t_0\right]$ and $n \ge N$,

$$\begin{aligned} \widetilde{F}_n(t)&\ge - \frac{9 \mathcal {C}_d^3 e^{ \frac{dN \ln 2 }{2}} t^{\frac{\mu }{2}-2}}{n H \left( 4 \pi \right) ^{d}\alpha _{d}} \left\| \mathcal {J}^-\left( \cdot ; u_1, \kappa _1 \right) \right\| _{L^1\left( \left[ 0, t\right] , s^{- \frac{d}{2}} ds \right) } \mathcal {S}_n\left( t; u_1, \kappa _1 \right) \\&\quad \times \left[ \left\| \mathcal {D}\left( \cdot ; u_2, \kappa _2 \right) \right\| _{L^1\left( \left[ 0, t\right] , s^{- \frac{d}{2}} ds \right) } + \frac{2t}{dn} \left\| \mathcal {T}\left( \cdot ; u_2, \kappa _2 \right) \right\| _{L^1\left( \left[ 0, t\right] , s^{- \frac{d}{2}} ds \right) } \right] \\&\quad - \frac{9 \mathcal {C}_d^3 e^{ \frac{dN \ln 2 }{2}} t^{\frac{\mu }{2}-2}}{n H \left( 4 \pi \right) ^{d}\alpha _{d}} \left\| \mathcal {J}^-\left( \cdot ; u_2, \kappa _2 \right) \right\| _{L^1\left( \left[ 0, t\right] , s^{- \frac{d}{2}} ds \right) } \mathcal {S}_n\left( t; u_2, \kappa _2 \right) \\&\quad \times \left[ \left\| \mathcal {D}\left( \cdot ; u_1, \kappa _1 \right) \right\| _{L^1\left( \left[ 0, t\right] , s^{- \frac{d}{2}} ds \right) } + \frac{2t}{dn} \left\| \mathcal {T}\left( \cdot ; u_1, \kappa _1 \right) \right\| _{L^1\left( \left[ 0, t\right] , s^{- \frac{d}{2}} ds \right) } \right] . \end{aligned}$$

Assuming that $\delta \le \varepsilon$, where $\varepsilon$ is from assumption (42), and using that $\displaystyle t \in \left[ \frac{t_0}{2}, t_0\right]$, it follows that

$$\begin{aligned} \inf _{t \in \left[ t_0 - \delta , t_0\right] } \widetilde{F}_n(t)&\ge - \frac{36 \mathcal {C}_d^3 e^{ \frac{dN \ln 2 }{2}} t_0^{\frac{\mu }{2} -2}}{n H \left( 4 \pi \right) ^{d}\alpha _{d}}\left\| \mathcal {J}^-\left( \cdot ; u_1, \kappa _1 \right) \right\| _{L^1\left( \left[ 0, t_0\right] , t^{- \frac{d}{2}} dt \right) }\\&\quad \times \left[ \left\| \mathcal {D}\left( \cdot ; u_2, \kappa _2 \right) \right\| _{L^1\left( \left[ 0, t_0\right] , t^{- \frac{d}{2}} dt \right) } + \frac{2t_0}{dn} \left\| \mathcal {T}\left( \cdot ; u_2, \kappa _2 \right) \right\| _{L^1\left( \left[ 0, t_0\right] , t^{- \frac{d}{2}} dt \right) } \right] \\&\quad \times \left[ \left\| \mathcal {D}\left( \cdot ; u_1, \kappa _1 \right) \right\| _{L^\infty \left[ t_0 - \delta , t_0\right] } + \frac{2t_0}{dn} \left\| \mathcal {T}\left( \cdot ; u_1, \kappa _1 \right) \right\| _{L^\infty \left[ t_0 - \delta , t_0\right] }\right] \\&- \frac{36 \mathcal {C}_d^3 e^{ \frac{dN \ln 2 }{2}} t_0^{\frac{\mu }{2}-2}}{n H \left( 4 \pi \right) ^{d}\alpha _{d}}\left\| \mathcal {T}^{-}\left( \cdot ; u_2, \kappa _2 \right) \right\| _{\left( \left[ 0, t\right] , t^{- \frac{d}{2}} dt \right) } \\&\quad \left[ \left\| \mathcal {D}\left( \cdot ; u_1, \kappa _1 \right) \right\| _{\left( \left[ 0, t_0\right] , t^{- \frac{d}{2}} dt \right) } + \frac{2t_0}{dn} \left\| \mathcal {T}\left( \cdot ; u_1, \kappa _1 \right) \right\| _{\left( \left[ 0, t_0\right] , t^{- \frac{d}{2}} dt \right) } \right] \\&\times \left[ \left\| \mathcal {D}\left( \cdot ; u_2, \kappa _2 \right) \right\| _{L^\infty \left[ t_0 - \delta , t_0\right] } + \frac{2t_0}{dn} \left\| \mathcal {T}\left( \cdot ; u_2, \kappa _2 \right) \right\| _{L^\infty \left[ t_0 - \delta , t_0\right] }\right] . \end{aligned}$$

In particular, this shows that ${\widetilde{F}}_n$ converges uniformly to 0, as required.

We have shown that the proof is complete under the assumption that the measures of $\text {supp} \, u_1\left( \cdot , t \right)$ and $\text {supp} \, u_2\left( \cdot , t \right)$ are non-vanishing for every *t*.

Now assume that there exists some values of *t* such that the measure of $\text {supp} \, u_1(\cdot , t)$ or the measure of $\text {supp} \, u_2(\cdot , t)$ vanishes. Let $\tau$ be the largest such *t*-value. Without loss of generality, we may assume that $|\text {supp} \, u_1(\cdot , \tau )| = 0$. Since $\Gamma _{1, n, \tau } = \text {supp} \ v_{1, n} \cap S_\tau ^n$, it follows that $|\Gamma _{1, n, \tau }| = 0$ for every *n* as well. Therefore, for every *n*, ${{\,\textrm{div}\,}}\left( \kappa _{1,n} \nabla v_{1,n} \right) \ge \kappa _{1,n} \ell _{1, n}$ in $D_{1, n, \tau }:= \text {supp} \, v_{1,n} \cap B_\tau ^n$ with $v_{1, n} = 0$ along $\partial D_{1, n, \tau }$. By the arguments used to reach estimate (40) applied to $v_{1,n}$ on $D_{1,n,\tau }$, we see that

$$\begin{aligned} \int _{D_{1, n, \tau }} \kappa _{1, n} |\nabla v_{1,n}|^2 |y|^{2 + \Upsilon _1 - d n} d{y} \le - \int _{D_{1, n, \tau }} \kappa _{1, n} \ell _{1,n} v_{1,n} |y|^{2 + \Upsilon _1 - d n} dy \end{aligned}$$

so that

$$\begin{aligned} \int _{B_\tau ^n} \kappa _{1, n} |\nabla v_{1,n}|^2 |y|^{2 + \Upsilon _1 - d n} d{y} \le - \int _{B_\tau ^n} \kappa _{1, n} \ell _{1,n} v_{1,n} |y|^{2 + \Upsilon _1 - d n} dy. \end{aligned}$$

As shown above,

$$\begin{aligned} \frac{\left( 2 d \right) ^{-\frac{\Upsilon _1}{2}}}{d n\left| S^{d n - 1 }\right| }&\int _{B_\tau ^n} \kappa _{1, n} |\nabla v_{1,n}|^2 |y|^{2 + \Upsilon _1 - d n} d{y} \\&= \int _0^t \int _{\mathbb {R}^d} s^{\frac{\Upsilon _1}{2}} \left[ \left\langle A_1 \nabla u_1, \nabla u_1 \right\rangle + \frac{2}{dn} \frac{ \partial u_1}{ \partial s} \left( \left\langle A_1 \nabla u_{1}, H_1^T h_1 \right\rangle + s \frac{ \partial u_1}{ \partial s} \right) \right] K_n(h_1(x),s)dx ds \end{aligned}$$

and

$$\begin{aligned}&\frac{\left( 2 d \right) ^{-\frac{\Upsilon _1}{2}}}{dn \left| S^{d n - 1 }\right| } \int _{B_\tau ^n} \kappa _{1, n} \ell _{1,n} v_{1,n} |y|^{2 + \Upsilon _1 - d n} dy \\&\quad = \frac{1}{n} \int _0^t \int _{\mathbb {R}^d} s^{\frac{\Upsilon _1}{2}} J_1^-(x, s) \, u_1\left( x, s \right) \, K_n(h_1(x),s)dx ds \end{aligned}$$

from which it follows that for every $n \in \mathbb {N}$,

$$\begin{aligned}&\int _0^t \int _{\mathbb {R}^d} s^{\frac{\Upsilon _1}{2}} \left\langle A_1 \nabla u_1, \nabla u_1 \right\rangle K_n(h_1(x),s)dx ds \\&\quad = \int _0^t \int _{\mathbb {R}^d} s^{\frac{\Upsilon _1}{2}} \left[ \left\langle A_1 \nabla u_1, \nabla u_1 \right\rangle + \frac{2}{dn} \frac{ \partial u_1}{ \partial s} \left( \left\langle A_1 \nabla u_{1}, H_1^T h_1 \right\rangle + s \frac{ \partial u_1}{ \partial s} \right) \right] K_n(h_1(x),s)dx ds \\&\qquad - \frac{2}{dn} \int _0^t \int _{\mathbb {R}^d} s^{\frac{\Upsilon _1}{2}} \frac{ \partial u_1}{ \partial s} \left( \left\langle A_1 \nabla u_{1}, H_1^T h_1 \right\rangle + s \frac{ \partial u_1}{ \partial s} \right) K_n(h_1(x),s)dx ds \\&\quad \le \frac{1}{n} \int _0^t \int _{\mathbb {R}^d} s^{\frac{\Upsilon _1}{2}} \left[ - J_1^-(x, s) \, u_1\left( x, s \right) - \frac{2}{d} \frac{ \partial u_1}{ \partial s} \left( \left\langle A_1 \nabla u_{1}, H_1^T h_1 \right\rangle + s \frac{ \partial u_1}{ \partial s} \right) \right] K_n(h_1(x),s)dx ds \\&\quad \le - \frac{2}{dn} \int _0^t \int _{\mathbb {R}^d} s^{\frac{\Upsilon _1}{2}} \left[ \frac{d}{2} J_1(x, s) \, u_1\left( x, s \right) + \frac{ \partial u_1}{ \partial s} \left( \left\langle A_1 \nabla u_{1}, H_1^T h_1 \right\rangle + s \frac{ \partial u_1}{ \partial s} \right) \right] K_n(h_1(x),s)dx ds, \end{aligned}$$

since $u_1 \ge 0$. With *J* as given in (25), we see that

$$\begin{aligned}&\frac{d}{2} J_1(x, s) \, u_1\left( x, s \right) + \frac{ \partial u_1}{ \partial s} \left( \left\langle A_1 \nabla u_{1}, H_1^T h_1 \right\rangle + s \frac{ \partial u_1}{ \partial s} \right) \\&\quad = \left[ \left\langle A_1 \nabla \frac{ \partial u_1}{ \partial s}, H_1^T h_1 \right\rangle + \frac{1}{2} \frac{ \partial u_1}{ \partial s} \text {tr}\left( H_1 \left\langle \nabla _z G_1(h_1), h_1 \right\rangle \right) + s \frac{ \partial ^2 u_1}{ \partial s^2} \right] \, u_1\left( x, s \right) \\&\qquad + \frac{ \partial u_1}{ \partial s} \left[ \left\langle A_1 \nabla u_{1}, H_1^T h_1 \right\rangle + s \frac{ \partial u_1}{ \partial s} \right] \\&\quad = \left\langle A_1 \nabla w_1, H_1^T h_1 \right\rangle + s \frac{ \partial w_1}{ \partial s} + \frac{w_1}{2} \text {tr}\left( H_1 \left\langle \nabla _z G_1(h_1), h_1 \right\rangle \right) , \end{aligned}$$

where we have introduced the notation $w_1 = u_1 \frac{ \partial u_1}{ \partial s}$. Therefore,

$$\begin{aligned}&\int _0^t \int _{\mathbb {R}^d} s^{\frac{\Upsilon _1}{2}} \left\langle A_1 \nabla u_1, \nabla u_1 \right\rangle K_n(h_1(x),s)dx ds \\&\quad \le - \frac{2 \mathcal {C}_d}{dn} \int _0^t \int _{\mathbb {R}^d} s^{\frac{\Upsilon _1}{2}} \left[ \left\langle A_1 \nabla w_1, H_1^T h_1 \right\rangle + s \frac{ \partial w_1}{ \partial s} + \frac{w_1}{2} \text {tr}\left( H_1 \left\langle \nabla _z G_1(h_1), h_1 \right\rangle \right) \right] K(h_1(x),s)dx ds. \end{aligned}$$

In particular,

$$\begin{aligned}&\int _0^t \int _{\mathbb {R}^d} s^{\frac{\Upsilon _1}{2}} \left\langle A_1 \nabla u_1, \nabla u_1 \right\rangle K_n(h_1(x),s)dx ds \\&\quad \le - \lim _{n \rightarrow \infty } \frac{2 \mathcal {C}_d}{dn} \int _0^t \int _{\mathbb {R}^d} s^{\frac{\Upsilon _1}{2}} \left[ \left\langle A_1 \nabla w_1, H_1^T h_1 \right\rangle + s \frac{ \partial w_1}{ \partial s} + \frac{w_1}{2} \text {tr}\left( H_1 \left\langle \nabla _z G_1(h_1), h_1 \right\rangle \right) \right] K(h_1(x),s)dx ds =0. \end{aligned}$$

Then $\Phi (t) = 0$ for every $t \le \tau$ showing that $\Phi (t)$ is monotonically non-decreasing on $(0, \tau ]$. Since $|\Gamma _{1, n, t}| \ne 0$ and $|\Gamma _{2, n, t}| \ne 0$ for every *n* and every $t > \tau$, then by the arguments from the first case, $\Phi (t,u)$ is monotonically non-decreasing whenever $t > \tau$, completing the proof. $\square$

## Data Availability

Data sharing not applicable to this article as no datasets were generated or analysed during the current study.

## Appendix A: Computational and technical proofs

Within this appendix, we have collected the proofs that are either purely computational or technical in nature. We begin with the proof of Lemma 2.2, which describes a collection of relationships between elliptic functions $v_n: B_{\sqrt{2 d T}} \subset \mathbb {R}^{d \times n} \rightarrow \mathbb {R}$ and parabolic functions $u: \mathbb {R}^d \times \left( 0, T \right) \rightarrow \mathbb {R}$ that are related through the transformation $F_{d,n}$. That is, Lemma 2.2 describes the relationships between derivatives of *u* and $v_n$ whenever $v_n(y) = u \left( F_{d,n}(y) \right) = u (x, t)$. The proof of this result relies on the chain rule.

### Proof of Lemma 2.2

Since $$\displaystyle \frac{ \partial z_k}{ \partial y_{i,j}} = \delta _{k, i}$$, then

$$\begin{aligned} \frac{ \partial v_n}{ \partial y_{i,j}}&= \frac{ \partial u}{ \partial x_1} \frac{ \partial g_1}{ \partial z_i} + \cdots + \frac{ \partial u}{ \partial x_d} \frac{ \partial g_d}{ \partial z_i} + \frac{ \partial u}{ \partial t} \frac{y_{i, j}}{d} = \left\langle \nabla _x u, \frac{ \partial g}{ \partial z_i} \right\rangle + \frac{ \partial u}{ \partial t} \frac{y_{i, j}}{d}, \end{aligned}$$

where $\displaystyle \frac{ \partial g}{ \partial z_i}$ is the $i^{th}$ column of the Jacobian matrix *G*. Then we have

$$\begin{aligned} y \cdot \nabla _y v_n&= \sum _{i=1}^d \sum _{j = 1}^n y_{i, j}\left[ \sum _{k=1}^d \frac{ \partial u}{ \partial x_k} \frac{ \partial g_k}{ \partial z_i} + \frac{ \partial u}{ \partial t} \frac{y_{i, j}}{d}\right] = \sum _{i, k=1}^d \frac{ \partial u}{ \partial x_k} \frac{ \partial g_k}{ \partial z_i} z_{i} + \frac{ \partial u}{ \partial t} \sum _{i=1}^d \sum _{j = 1}^n \frac{y_{i, j}^2}{d} \\&= \left\langle \nabla _x u, G(z) z \right\rangle + 2 t \frac{ \partial u}{ \partial t} = \left\langle A(x)\nabla _x u, H(x)^T h(x) \right\rangle + 2 t \frac{ \partial u}{ \partial t} \end{aligned}$$

and

$$\begin{aligned} \left| \nabla _y v_n\right| ^2&= \sum _{i=1}^d \sum _{j = 1}^n \left( \frac{ \partial v_n}{ \partial y_{i,j}} \right) ^2 = \sum _{i=1}^d \sum _{j = 1}^n\left[ \sum _{k=1}^d \frac{ \partial u}{ \partial x_k} \frac{ \partial g_k}{ \partial z_i} + \frac{ \partial u}{ \partial t} \frac{y_{i, j}}{d}\right] \left[ \sum _{\ell =1}^d \frac{ \partial u}{ \partial x_\ell } \frac{ \partial g_\ell }{ \partial z_i} + \frac{ \partial u}{ \partial t} \frac{y_{i, j}}{d}\right] \\&= n \sum _{i, k, \ell =1}^d \left( \frac{ \partial u}{ \partial x_k} \frac{ \partial g_k}{ \partial z_i} \right) \left( \frac{ \partial u}{ \partial x_\ell } \frac{ \partial g_\ell }{ \partial z_i} \right) + \frac{2}{d} \frac{ \partial u}{ \partial t} \sum _{i, k=1}^d \left( \frac{ \partial u}{ \partial x_k} \frac{ \partial g_k}{ \partial z_i} \right) z_{i} + \left( \frac{ \partial u}{ \partial t} \right) ^2 \sum _{i=1}^d \sum _{j = 1}^n \left( \frac{y_{i, j}}{d} \right) ^2 \\&= n \left| G(z)^T \nabla _x u\right| ^2 + \frac{2}{d} \frac{ \partial u}{ \partial t} \left[ \left\langle \nabla _x u, G(z) \, z \right\rangle + t \frac{ \partial u}{ \partial t} \right] . \end{aligned}$$

Now we look at the second-order derivatives. Since $\displaystyle \kappa _n(y) \frac{ \partial v_n}{ \partial y_{i,j}} = \sum _{k=1}^d \frac{ \partial u}{ \partial x_k} \gamma (z) \frac{ \partial g_k}{ \partial z_i} + \frac{ \partial u}{ \partial t} \gamma (z) \frac{y_{i, j}}{d}$, then

$$\begin{aligned} \frac{ \partial }{ \partial y_{i,j}}\left( \kappa _n(y)\frac{ \partial v_n}{ \partial y_{i,j}} \right)&= \frac{ \partial }{ \partial y_{i,j}}\left( \sum _{k=1}^d \frac{ \partial u}{ \partial x_k} \gamma (z) \frac{ \partial g_k}{ \partial z_i} \right) + \frac{ \partial }{ \partial y_{i,j}}\left( \frac{ \partial u}{ \partial t} \gamma (z) \frac{y_{i, j}}{d} \right) \\&= \sum _{k, \ell =1}^d \frac{ \partial ^2 u}{ \partial x_\ell \partial x_k} \gamma \frac{ \partial g_k}{ \partial z_i} \frac{ \partial g_\ell }{ \partial z_i} + 2\sum _{k=1}^d \frac{ \partial ^2 u}{ \partial t \partial x_k} \gamma \frac{ \partial g_k}{ \partial z_i} \frac{y_{ij}}{d} + \sum _{k=1}^d \frac{ \partial u}{ \partial x_k} \frac{ \partial \gamma }{ \partial z_{i}} \frac{ \partial g_k}{ \partial z_i} \\&\quad + \sum _{k=1}^d \frac{ \partial u}{ \partial x_k} \gamma \frac{ \partial ^2 g_k}{ \partial z_i^2} \\&\quad +\frac{ \partial ^2 u}{ \partial t^2} \gamma \left( \frac{y_{i, j}}{d} \right) ^2 + \frac{ \partial u}{ \partial t} \frac{ \partial \gamma }{ \partial z_i} \frac{y_{i, j}}{d} + \frac{ \partial u}{ \partial t} \gamma \frac{1}{d}, \end{aligned}$$

from which it follows that

46 $$\begin{aligned} \begin{aligned} \frac{1}{\kappa _n(y)}\frac{ \partial }{ \partial y_{i,j}}\left( \kappa _n(y)\frac{ \partial v_n}{ \partial y_{i,j}} \right)&= \left\langle D^2_x u \, \frac{ \partial g}{ \partial z_i}, \frac{ \partial g}{ \partial z_i} \right\rangle + \left\langle \nabla _x u, \frac{ \partial \log \gamma }{ \partial z_{i}} \frac{ \partial g}{ \partial z_i} + \frac{ \partial ^2 g}{ \partial z_{i}^2} \right\rangle + \frac{1}{d} \frac{ \partial u}{ \partial t} \\&\quad + 2 \left\langle \nabla _x \left( \frac{ \partial u}{ \partial t} \right) , \frac{ \partial g}{ \partial z_i} \frac{y_{i, j}}{d} \right\rangle + \frac{ \partial \log \gamma }{ \partial z_{i}} \frac{ \partial u}{ \partial t}\frac{y_{i, j}}{d} + \frac{ \partial ^2 u}{ \partial t^2} \left( \frac{y_{i, j}}{d} \right) ^2. \end{aligned}\nonumber \\ \end{aligned}$$

Because $$B(z) = G(z) G^T(z) = A(x)$$, then

47 $$\begin{aligned} \sum _{i =1}^d \left\langle D^2_x u \, \frac{ \partial g}{ \partial z_i}, \frac{ \partial g}{ \partial z_i} \right\rangle&= \sum _{i, k, \ell =1}^d \frac{ \partial ^2 u}{ \partial x_k \partial x_\ell } \frac{ \partial g_k}{ \partial z_i} \frac{ \partial g_\ell }{ \partial z_{i}} = \sum _{k, \ell =1}^d b_{k, \ell }(z) \frac{ \partial ^2 u}{ \partial x_k \partial x_\ell } = \sum _{k, \ell =1}^d a_{k, \ell }(x) \frac{ \partial ^2 u}{ \partial x_k \partial x_\ell }. \end{aligned}$$

Since $$\displaystyle a_{k, \ell }(x) = \sum _{i=1}^d \frac{ \partial g_k}{ \partial z_i}\left( h(x) \right) \frac{ \partial g_\ell }{ \partial z_i}\left( h(x) \right) $$, then

48 $$\begin{aligned} \begin{aligned} \frac{ \partial a_{k, \ell }}{ \partial x_k}&= \sum _{i=1}^d \frac{ \partial }{ \partial x_k}\left( \frac{ \partial g_k}{ \partial z_i}\left( h(x) \right) \right) \frac{ \partial g_\ell }{ \partial z_i}\left( h(x) \right) + \sum _{i=1}^d \frac{ \partial g_k}{ \partial z_i}\left( h(x) \right) \frac{ \partial }{ \partial x_k}\left( \frac{ \partial g_\ell }{ \partial z_i}\left( h(x) \right) \right) \\&= \sum _{i, m=1}^d \frac{ \partial ^2 g_k\left( z \right) }{ \partial z_i \partial z_m} \frac{ \partial g_\ell \left( z \right) }{ \partial z_i} \frac{ \partial h_m}{ \partial x_k}\left( g(z) \right) + \sum _{i, m=1}^d \frac{ \partial ^2 g_\ell \left( z \right) }{ \partial z_i \partial z_m} \frac{ \partial h_m}{ \partial x_k}\left( g(z) \right) \frac{ \partial g_k\left( z \right) }{ \partial z_i}. \end{aligned} \end{aligned}$$

Summing (48) over *k*, substituting (7) and using that $\sum _{k =1}^d \frac{ \partial h_m}{ \partial x_k} \frac{ \partial g_k}{ \partial z_i} = \delta _{m, i}$ since $HG = I$, shows that

49 $$\begin{aligned} \sum _{k =1}^d \frac{ \partial a_{k, \ell }}{ \partial x_k}&= \sum _{i, m, k=1}^d \frac{ \partial h_m}{ \partial x_k}\left( g(z) \right) \frac{ \partial ^2 g_k\left( z \right) }{ \partial z_i \partial z_m} \frac{ \partial g_\ell \left( z \right) }{ \partial z_i} + \sum _{i, m, k=1}^d \frac{ \partial ^2 g_\ell \left( z \right) }{ \partial z_i \partial z_m} \frac{ \partial h_m}{ \partial x_k}\left( g(z) \right) \frac{ \partial g_k\left( z \right) }{ \partial z_i} \nonumber \\&= \sum _{i=1}^d \frac{ \partial \log \gamma (z)}{ \partial z_i} \frac{ \partial g_\ell \left( z \right) }{ \partial z_i} + \sum _{i =1}^d \frac{ \partial ^2 g_\ell \left( z \right) }{ \partial z_i^2}. \end{aligned}$$

Therefore,

50 $$\begin{aligned} {{\,\textrm{div}\,}}_x \left( A \nabla _x u \right)&= \sum _{k = 1}^d \frac{ \partial }{ \partial x_k} \left( \sum _{\ell = 1}^d a_{k, \ell } \frac{ \partial u}{ \partial x_\ell } \right) = \sum _{k, \ell = 1}^d a_{k, \ell } \frac{ \partial ^2 u}{ \partial x_\ell \partial x_k} + \sum _{\ell = 1}^d \left( \sum _{k = 1}^d\frac{ \partial a_{k, \ell }}{ \partial x_k} \right) \frac{ \partial u}{ \partial x_\ell } \nonumber \\&=\sum _{i =1}^d \left\langle D^2_x u \, \frac{ \partial g}{ \partial z_i}, \frac{ \partial g}{ \partial z_i} \right\rangle + \left\langle \nabla _x u, \frac{ \partial \log \gamma }{ \partial z_{i}} \frac{ \partial g}{ \partial z_i} + \frac{ \partial ^2 g}{ \partial z_i ^2} \right\rangle , \end{aligned}$$

where we have used (47) and (49). Summing (46) over *i* and *j* and substituting (50) then shows that

$$\begin{aligned} \begin{aligned}&\frac{1}{\kappa _n(y)} {{\,\textrm{div}\,}}_y \left( \kappa _n(y)\nabla _y v_n \right) \\&\quad = \frac{1}{\kappa _n(y)} \sum _{i=1}^d \sum _{j=1}^n \frac{ \partial }{ \partial y_{i,j}} \left( \kappa _n(y)\frac{ \partial v_n}{ \partial y_{i,j}} \right) \\&\quad = \sum _{j=1}^n \sum _{i=1}^d \left[ \left\langle D^2_x u \, \frac{ \partial g}{ \partial z_i}, \frac{ \partial g}{ \partial z_i} \right\rangle + \left\langle \nabla _x u, \frac{ \partial \log \gamma }{ \partial z_{i}} \frac{ \partial g}{ \partial z_i} + \frac{ \partial ^2 g}{ \partial z_{i}^2} \right\rangle + \frac{1}{d} \frac{ \partial u}{ \partial t}\right] \\&\qquad + \sum _{i=1}^d \sum _{j=1}^n \left[ 2 \left\langle \nabla _x \left( \frac{ \partial u}{ \partial t} \right) , \frac{ \partial g}{ \partial z_i} \frac{y_{i, j}}{d} \right\rangle + \frac{ \partial \log \gamma }{ \partial z_{i}} \frac{ \partial u}{ \partial t}\frac{y_{i, j}}{d} + \frac{ \partial ^2 u}{ \partial t^2} \left( \frac{y_{i, j}}{d} \right) ^2 \right] \\&\quad = \sum _{j=1}^n \left[ {{\,\textrm{div}\,}}_x \left( A \nabla _x u \right) + \frac{ \partial u}{ \partial t}\right] + \frac{2}{d} \sum _{i, k = 1}^d \frac{ \partial ^2 u}{ \partial x_k \partial t} \frac{ \partial g_k}{ \partial z_i}(z) z_i \\&\qquad + \frac{1}{d} \frac{ \partial u}{ \partial t} \sum _{i =1}^d \frac{ \partial \log \gamma }{ \partial z_i}(z) z_i + \frac{2t}{d} \frac{ \partial ^2 u}{ \partial t^2} \\&\quad = n \left\{ {{\,\textrm{div}\,}}_x \left( A \nabla _x u \right) + \frac{ \partial u}{ \partial t} + \frac{1}{dn} \left[ 2 \left\langle \nabla _x \frac{ \partial u}{ \partial t}, G(z) z \right\rangle + \frac{ \partial u}{ \partial t} \left\langle \nabla \log \gamma (z), z \right\rangle + 2t \frac{ \partial ^2 u}{ \partial t^2} \right] \right\} , \end{aligned} \end{aligned}$$

and the result follows from changing the last set of terms to depend on *x*. $\square$

Here we prove Lemma 3.1 regarding the uniform convergence of our sequence of approximations to the Gaussian.

### Proof of Lemma 3.1

Let

51 $$\begin{aligned} \alpha _{d,n}&=\frac{|S^{d n -1-d}|}{|S^{d n-1}|}\left( \frac{2\pi }{dn}\right) ^{\frac{d}{2}} = \left( \frac{2 \pi }{d n} \right) ^{\frac{d}{2}} \frac{2 \pi ^{\frac{d n- d}{2}}}{\Gamma \left( \frac{d n- d}{2} \right) } \frac{\Gamma \left( \frac{d n}{2} \right) }{2 \pi ^{\frac{d n}{2}}} = \left( \frac{2}{d n} \right) ^{\frac{d}{2}} \frac{\Gamma \left( \frac{ d n}{2} \right) }{\Gamma \left( \frac{d n}{2} - \frac{d}{2} \right) } . \end{aligned}$$

If $$\frac{d}{2} \in \mathbb {Z}$$, then

$$\begin{aligned} \Gamma \left( \frac{d n}{2} \right)&= \left( \frac{d n}{2} - 1 \right) \left( \frac{d n}{2} - 2 \right) \ldots \left( \frac{d n}{2} - \frac{d}{2} \right) \Gamma \left( \frac{d n}{2} - \frac{d}{2} \right) \\&= \left( \frac{d n}{2} \right) ^{\frac{d}{2}} \left( 1 - \frac{2}{d n} \right) \left( 1 - \frac{4}{d n} \right) \ldots \left( 1 - \frac{d}{d n} \right) \Gamma \left( \frac{d n}{2} - \frac{d}{2} \right) \end{aligned}$$

and we see that

$$\begin{aligned} \alpha _{d,n} = \prod _{j=1}^{\frac{d}{2}} \left( 1 - \frac{2j}{d n} \right) . \end{aligned}$$

Stirling’s formula shows that

$$\begin{aligned} \Gamma (z) = \sqrt{\frac{2\pi }{z}} \left( \frac{z}{e} \right) ^z \left( 1 + \mathcal {O}\left( \frac{1}{z} \right) \right) \end{aligned}$$

so we see that

$$\begin{aligned} \alpha _{d,n}&= \left( \frac{2}{n d} \right) ^{\frac{d}{2}} \frac{\sqrt{\frac{2\pi }{\frac{d n}{2}}} \left( \frac{\frac{d n}{2}}{e} \right) ^{\frac{d n}{2}} \left( 1 + \mathcal {O}\left( \frac{2}{d n} \right) \right) }{\sqrt{\frac{2\pi }{{\frac{d n}{2} - \frac{d}{2}}}} \left( \frac{\frac{d n}{2} - \frac{d}{2}}{e} \right) ^{\frac{d n}{2} - \frac{d}{2}}\left( 1 + \mathcal {O}\left( \frac{2}{d n- d} \right) \right) } \\&= \left[ e\left( 1 - \frac{1}{n} \right) ^{n}\right] ^{-\frac{d}{2}} \left( 1 - \frac{1}{n} \right) ^{\frac{d+1}{2}} \left( 1 + \mathcal {O}\left( \frac{2}{d n} \right) \right) . \end{aligned}$$

In both cases, it holds that $\alpha _{d,n} \rightarrow 1$ as $n \rightarrow \infty$. Therefore, there exists $\alpha _d$ so that for every $n \in \mathbb {N}$,

52 $$\begin{aligned} \alpha _{d,n} \le \alpha _d. \end{aligned}$$

To analyze the remaining piece of $K_n(x,t)$, let $m=\frac{d n}{2}$, $\delta =\frac{d+2}{2}$, $z=\frac{|x|^2}{4t}$. Notice that $|x|^2<2dnt$ if and only if $0\le z<m$, and $\left( 1-\frac{|x|^2}{2dnt}\right) ^{\frac{d n-d-2}{2}}=\left( 1-\frac{z}{m}\right) ^{m-\delta }.$ Define

$$\begin{aligned} f_m(z)=\left( 1-\frac{z}{m}\right) ^{m-\delta }\chi _{\{0\le z< m\}}(z). \end{aligned}$$

We show $f_m$ converges uniformly to $f(z)=e^{-z}\chi _{\{z\ge 0\}}(z)$. First, notice that if $z>m$, then $|f(z)-f_m(z)|=e^{-z}<e^{-m}$.

If $$0\le z\le m$$, then

53 $$\begin{aligned} \begin{aligned} |f(z)-f_m(z)|&=\left| e^{-z}-\left( 1-\frac{z}{m}\right) ^m+\left( 1-\frac{z}{m}\right) ^m-\left( 1-\frac{z}{m}\right) ^{m-\delta }\right| \\&\le \left| e^{-z}-\left( 1-\frac{z}{m}\right) ^m\right| +\left| \left( 1-\frac{z}{m}\right) ^{m-\delta }-\left( 1-\frac{z}{m}\right) ^m\right| =: \text {(I)} +\text {(II)}. \end{aligned}\nonumber \\ \end{aligned}$$

We address (I) by showing there exists a constant *c* such that for all $m \ge 1$

$$\begin{aligned} \max _{0< z < m} \left| e^{-z} - \left( 1 - \frac{z}{m}\right) ^m \right| \le c \frac{(\log {m})^2}{m}.\end{aligned}$$

Indeed, we have

$$\begin{aligned} \log \left[ \left( 1 - \frac{z}{m}\right) ^m\right] = m \log \left( 1 - \frac{z}{m} \right) . \end{aligned}$$

For $0< z < m$, this means that $\log \left( 1-\frac{z}{m}\right) \in (0,1)$. Since $\log {x} < x-1$ for all $x > 0$, then

$$\begin{aligned} \log \left[ \left( 1 - \frac{z}{m}\right) ^m\right] \le - z. \end{aligned}$$

Consequently, $\left( 1-\frac{z}{m}\right) ^m$ is smaller than $e^{-z}$. It remains to obtain an upper bound on

$$\begin{aligned} \max _{0 \le z \le m} \left[ e^{-z} - \left( 1 - \frac{z}{m}\right) ^m\right] . \end{aligned}$$

Using a Taylor expansion, we have

$$\begin{aligned} \log {(1-x)} = -x - \frac{x^2}{2} + \mathcal {O}(x^3). \end{aligned}$$

Thus

$$\begin{aligned} \log \left[ \left( 1 - \frac{z}{m}\right) ^m\right] = -z - \frac{z^2}{2m} + \mathcal {O}\left( \frac{z^3}{m^2}\right) , \end{aligned}$$

and

$$\begin{aligned} \left( 1 - \frac{z}{m}\right) ^m = e^{-z} e^{-\frac{z^2}{2m}} e^{\mathcal {O}\left( z^3/m^2\right) }. \end{aligned}$$

We split the problem into two parts, by bounding

$$\begin{aligned} \max _{0 \le z \le \alpha } \left[ e^{-z} - \left( 1 - \frac{z}{m}\right) ^m\right] \end{aligned}$$

and

$$\begin{aligned} \max _{\alpha \le z \le m} \left[ e^{-z} - \left( 1 - \frac{z}{m}\right) ^m\right] \end{aligned}$$

for some $\alpha$ to be determined. For the second part, observe that

$$\begin{aligned} \max _{\alpha \le z \le m} \left[ e^{-z} - \left( 1 - \frac{z}{m}\right) ^m\right] \le \max _{\alpha \le z \le m} e^{-z} = e^{-\alpha }. \end{aligned}$$

As for the first bound, we note that

$$\begin{aligned} e^{-z} - \left( 1 - \frac{z}{m}\right) ^m = e^{-z} \left( 1 -e^{-\frac{z^2}{2m}} e^{\mathcal {O}\left( \frac{z^3}{m^2}\right) }\right) . \end{aligned}$$

If $\frac{z^2}{2\,m} \rightarrow 0$, then $\frac{z^3}{m^2} \rightarrow 0$ as well and we can bound this term using another Taylor approximation as

$$\begin{aligned} e^{-z} \left( 1 -e^{-\frac{z^2}{2m}} e^{\mathcal {O}\left( \frac{z^3}{m^2}\right) }\right) = \mathcal {O}\left( \frac{z^2}{m}\right) . \end{aligned}$$

By choosing $\alpha = \log {m}$, we obtain

$$\begin{aligned} \max _{\alpha \le z \le m} \left[ e^{-z} - \left( 1 - \frac{z}{m}\right) ^m \right] \le \max _{\alpha \le z \le m} e^{-z} = e^{-\alpha } \le \frac{1}{m} \end{aligned}$$

and

$$\begin{aligned} \max _{0 \le z \le \alpha } \left[ e^{-z} - \left( 1 - \frac{z}{m}\right) ^m\right] \le \mathcal {O}\left( \frac{(\log {m})^2}{m} \right) . \end{aligned}$$

This proves that $\displaystyle \text {(I)} \le c \frac{(\log {m})^2}{m}$.

To estimate (II), let $g(x)=(1-x)^{m-\delta }-(1-x)^m$, with $0\le x\le 1$. Notice that $g(0)=g(1)=0$ and $g(x)>0$ for $x\in (0,1)$. Moreover,

$$\begin{aligned} g'(x)=-(m-\delta )(1-x)^{m-\delta -1}+m(1-x)^{m-1}=(1-x)^{m-1-\delta }\left[ m(1-x)^{\delta }-(m-\delta )\right] . \end{aligned}$$

Therefore $g'(x)\ge 0$ if and only if $x\le 1-\left( 1-\frac{\delta }{m}\right) ^{1/\delta }.$ We conclude that *g* attains its maximum at $x_0=1-\left( 1-\frac{\delta }{m}\right) ^{1/\delta }$, and

$$\begin{aligned} g(x_0)=\frac{\delta }{m}\left( 1-\frac{\delta }{m}\right) ^{\frac{m}{\delta }-1}. \end{aligned}$$

Consequently, $g(x)\in \left[ 0,\frac{\delta }{m}\right]$ for all $x\in [0,1]$.

By combining the estimates obtained for (I) and (II) and (53), we conclude that for all $z \ge 0$,

$$\begin{aligned} |f(z)-f_m(z)|\le c\frac{\left( \log m\right) ^2}{m} +e^{-m}+\frac{\delta }{m}. \end{aligned}$$

In particular, this shows that $f_m \rightarrow f$ uniformly, and the conclusion of the lemma follows. $\square$

Finally, we give the computational proof of Lemma 3.2.

### Proof of Lemma 3.2

Let $u_K(x, t) = K(h(x), t) = \frac{1}{\left( 4 \pi t \right) ^{d/2}} \exp \left( - \frac{\left| h(x)\right| ^2}{4t} \right)$. Computations show that

$$\begin{aligned} \frac{ \partial u_K}{ \partial t }&= - \frac{d}{2t} u_K + \frac{\left| h(x)\right| ^2}{\left( 2t \right) ^2} u_K \end{aligned}$$

and

$$\begin{aligned} \frac{ \partial u_K}{ \partial {x_i}}&= - \frac{1}{2t} \sum _{j=1}^d \frac{ \partial h_j}{ \partial {x_i} }\, h_j \, u_K. \end{aligned}$$

Since $a_{k,i} = \sum _{\ell =1}^d \frac{ \partial g_k}{ \partial {z_\ell } }(h(x)) \frac{ \partial g_i}{ \partial {z_\ell } }(h\left( x \right) )$ and $\frac{ \partial g_i}{ \partial {z_\ell } }(h\left( x \right) ) \frac{ \partial h_j}{ \partial {x_i} }(x) = \delta _{\ell , j}$, then

$$\begin{aligned} \sum _{i = 1}^d a_{k,i} \frac{ \partial u_K}{ \partial {x_i}}&= - \frac{1}{2t} \sum _{i, j, \ell =1}^d \frac{ \partial g_k}{ \partial {z_\ell } }(h(x)) \frac{ \partial g_i}{ \partial {z_\ell } }(h\left( x \right) ) \frac{ \partial h_j}{ \partial {x_i} }(x) h_j(x) u_K \\&= - \frac{1}{2t} \sum _{ j=1}^d \frac{ \partial g_k}{ \partial {z_j} }(h(x)) h_j(x) u_K. \end{aligned}$$

Therefore

$$\begin{aligned} {{\,\textrm{div}\,}}\left( A \nabla u_K \right)&= \sum _{k = 1}^d \frac{ \partial }{ \partial x_k} \left[ \sum _{i = 1}^d a_{k,i} \frac{ \partial u_K}{ \partial {x_i}} \right] = - \frac{1}{2t} \sum _{k = 1}^d \frac{ \partial }{ \partial x_k} \left[ \sum _{ j=1}^d \frac{ \partial g_k}{ \partial {z_j} }(h(x)) h_j(x) u_K\right] \\&= - \frac{1}{2t} \sum _{ j,k=1}^d \frac{ \partial g_k}{ \partial {z_j} }(h(x)) h_j(x) \frac{ \partial u_K}{ \partial x_k} \\&\quad - \frac{1}{2t} \sum _{ j,k=1}^d \left( \frac{ \partial g_k}{ \partial {z_j} }(h(x)) \frac{ \partial h_j}{ \partial x_k} + \frac{ \partial ^2 g_k}{ \partial {z_j} \partial z_\ell }(h(x)) \frac{ \partial h_\ell }{ \partial x_k} h_j \right) u_K \\&= \frac{1}{\left( 2t \right) ^2} \sum _{ j, k, \ell =1}^d \frac{ \partial g_k}{ \partial {z_j} }(h(x)) h_j \frac{ \partial h_\ell }{ \partial {x_k} } h_\ell u_K \\&\quad - \frac{1}{2t} \sum _{ j,k=1}^d\left( \frac{ \partial g_k}{ \partial {z_j} }(h(x)) \frac{ \partial h_j}{ \partial x_k} + \frac{ \partial ^2 g_k}{ \partial {z_j} \partial z_\ell }(h(x)) \frac{ \partial h_\ell }{ \partial x_k} h_j \right) u_K \\&= \frac{\left| h(x)\right| ^2}{\left( 2t \right) ^2} u_K - \frac{d}{2t} u_K - \frac{1}{2t} \sum _{ j, k, \ell =1}^d \frac{ \partial ^2 g_k}{ \partial {z_j} \partial z_\ell }(h(x)) \frac{ \partial h_\ell }{ \partial x_k} h_j \, u_K \\&= \frac{ \partial u_K}{ \partial t } - \frac{1}{2t} \sum _{ j, k, \ell =1}^d \frac{ \partial ^2 g_k}{ \partial {z_j} \partial z_\ell }(h(x)) \frac{ \partial h_\ell }{ \partial x_k} h_j \, u_K, \end{aligned}$$

as claimed. $$\square $$
